# The genome of the biting midge *Culicoides sonorensis* and gene expression analyses of vector competence for bluetongue virus

**DOI:** 10.1186/s12864-018-5014-1

**Published:** 2018-08-22

**Authors:** Ramiro Morales-Hojas, Malcolm Hinsley, Irina M. Armean, Rhiannon Silk, Lara E. Harrup, Asier Gonzalez-Uriarte, Eva Veronesi, Lahcen Campbell, Dana Nayduch, Christopher Saski, Walter J. Tabachnick, Paul Kersey, Simon Carpenter, Mark Fife

**Affiliations:** 1 The Pirbright Institute, Ash Road, Woking, Surrey, GU24 0NF UK; 2 Rothamsted Insect Survey, Rothamsted Research, West Common, Harpenden, Hertfordshire, AL5 2JQ UK; 3 EMBL-European Bioinformatics Institute, Wellcome Trust Genome Campus, Hinxton, Cambridge, CB10 1SD UK; 4 Bioinformatics group, Rothamsted Research, West Common, Harpenden, Hertfordshire, AL5 2JQ UK; 5 National Centre for Vector Entomology, Institute of Parasitology, Vetsuisse Faculty, University of Zürich, Zürich, Switzerland; 6 USDA-ARS, Center for Grain and Animal Health Research, Arthropod Borne Animal Diseases Research Unit, 1515 College Avenue, Manhattan, KS 66502 USA; 7 Department of Genetics and Biochemistry, Clemson University Genomics Institute, BRC #310, 105 Collins Street, Clemson, SC 29634 USA; 8 Florida Medical Entomology Laboratory, Department of Entomology and Nematology, University of Florida, IFAS, 200 9th St., SE, Vero Beach, FL 32962 USA

**Keywords:** Culicoides, Biting midges, Bluetongue virus, Genome, Genomics, Transcriptomics, Vector competence

## Abstract

**Background:**

The new genomic technologies have provided novel insights into the genetics of interactions between vectors, viruses and hosts, which are leading to advances in the control of arboviruses of medical importance. However, the development of tools and resources available for vectors of non-zoonotic arboviruses remains neglected. Biting midges of the genus *Culicoides* transmit some of the most important arboviruses of wildlife and livestock worldwide, with a global impact on economic productivity, health and welfare. The absence of a suitable reference genome has hindered genomic analyses to date in this important genus of vectors. In the present study, the genome of *Culicoides sonorensis,* a vector of bluetongue virus (BTV) in the USA, has been sequenced to provide the first reference genome for these vectors. In this study, we also report the use of the reference genome to perform initial transcriptomic analyses of vector competence for BTV.

**Results:**

Our analyses reveal that the genome is 189 Mb, assembled in 7974 scaffolds. Its annotation using the transcriptomic data generated in this study and in a previous study has identified 15,612 genes. Gene expression analyses of *C. sonorensis* females infected with BTV performed in this study revealed 165 genes that were differentially expressed between vector competent and refractory females. Two candidate genes, *glutathione S-transferase* (*gst*) and the antiviral helicase *ski2*, previously recognized as involved in vector competence for BTV in *C. sonorensis* (*gst*) and repressing dsRNA virus propagation (*ski2*), were confirmed in this study.

**Conclusions:**

The reference genome of *C. sonorensis* has enabled preliminary analyses of the gene expression profiles of vector competent and refractory individuals. The genome and transcriptomes generated in this study provide suitable tools for future research on arbovirus transmission. These provide a valuable resource for these vector lineage, which diverged from other major Dipteran vector families over 200 million years ago. The genome will be a valuable source of comparative data for other important Dipteran vector families including mosquitoes (Culicidae) and sandflies (Psychodidae), and together with the transcriptomic data can yield potential targets for transgenic modification in vector control and functional studies.

**Electronic supplementary material:**

The online version of this article (10.1186/s12864-018-5014-1) contains supplementary material, which is available to authorized users.

## Background

Arboviruses (arthropod-borne viruses) are a taxonomically diverse group that include some of the most important emerging and re-emerging pathogens of wildlife, livestock and human beings worldwide [1–3]. Among arbovirus vectors, the majority of recent genomic studies have been carried out on the mosquitoes *Aedes* (*Stegomyia*) *aegypti* (L.) and *Aedes* (*Stegomyia*) *albopictus* (Skuse) due to their involvement in human to human transmission of a wide-range of arboviruses, their relative ease of colonization and recent technical advances in genome sequencing and annotation technologies [4]. While these studies have led to major advances in control of the arboviruses these species transmit and our understanding of what drives susceptibility to infection [5–7], tools and resources for use with many other vector groups remain neglected.

To date, no genomic analyses have been carried out for vectors of non-zoonotic arboviruses that are pathogenic to livestock and wildlife. Among the most important of these vector groups are *Culicoides* biting midges (Diptera: Ceratopogonidae), species of which transmit internationally important arboviruses of ruminants, equines and deer [8–10]. In recent years, unprecedented outbreaks of *Culicoides*-borne arboviruses such as bluetongue virus (BTV) have inflicted huge economic losses on the livestock sector of Europe (e.g. estimate of US$ 1.4 billion in France in 2007) through clinical disease and accompanying restrictions against the movement of livestock imposed to limit virus spread [11, 12]. Globally, BTV outbreaks initiate non-tariff trade barriers restricting the movement of livestock and livestock germplasm, and cause a decrease in ruminant productivity in regions as diverse as India and the USA [13]. Globally, the economic impact of bluetongue has been estimated to US$ 3 billion [12].

*Culicoides* are notoriously difficult to culture under laboratory conditions and their small size of approximately 1.5 mm body length renders them far from ideal subjects for transcriptomic and genome manipulation studies [14]. Only one of 14 confirmed vector species of *Culicoides* is currently colonized, *Culicoides sonorensis* Wirth and Jones [8, 15]. *Culicoides sonorensis* colonies have already provided valuable insights into the genetic basis of vector competence for BTV [16], a de novo transcriptome [17], and have been used to construct a physical map of the *C. sonorensis* genome which consists of four chromosomes [18, 19].

Susceptibility to infection and transmission of arboviruses by *Culicoides* is determined in part by the heritability of barriers to virus dissemination following ingestion of the bloodmeal [13, 20, 21]. Experimental evidence of a midgut infection barrier, a midgut escape barrier and a haemocoel dissemination barrier in *C. sonorensis* (formerly *C. variipennis* or *C. v. sonorensis* [22]) have been defined [23]. There is no evidence to date of salivary gland barriers preventing transmission in any species of *Culicoides* [24], as has been inferred in several species of mosquitoes [20]. Previous studies of the genetic basis for vector competence of *C. sonorensis* for BTV, using maternal inheritance properties, identified a 90 kd protein and used antibodies to this protein to isolate and characterize a cDNA clone encoding a glutathione S-transferase class delta enzyme [25]. Differential gene regulation in response to BTV infection, however, has not been investigated to date.

In this study, we sequence, de novo assemble, annotate and explore the first full genome of *C. sonorensis*. We then use transcriptomic analyses both to improve gene prediction within the genome build, and to elucidate differential gene expression associated with *Culicoides* competence for a BTV serotype 1 strain. The full genome sequence and transcriptome analyses are important resources for further studies of this phylogenetic group which is separated from the other major Dipteran vector families by at least 220 million years [26]. In addition to being a valuable resource for comparative study of vector phylogenomics, the provision of *Culicoides* genomes is of interest as a group that demonstrates several unique features, including a great capacity for long-distance dispersal by semi-passive flight and the ability to reach huge population density under suitable conditions. In addition, their close association with livestock raises questions concerning both host preference and vector competence for arboviruses. Many of these questions can be readily addressed by understanding genetic diversity within populations which will be enhanced by the provision of comparative genomic data, but also in the long term by yielding targets for transgenic modification. The present genome provides a resource to facilitate all these studies.

## Methods

### Samples

All *C. sonorensis* used in this study originated from the ‘AA’ colony which was originally established in 1955 at the Kerrville, Texas laboratory of the US Department of Agriculture [27]. Since 1969, this colony has been maintained at The Pirbright Institute without any additional outbreeding [28]. Within this period, two selection bottlenecks of ≤10 individuals were performed to increase susceptibility to BTV serotype 4 and African horse sickness virus (AHSV) serotype 9 infection (Mellor, Pers Comm). Following the second selection in the 1990’s [29], the colony strain was renamed as PIR-s-3. No attempt was made to further reduce heterozygosity prior to using individuals from the PIR-s-3 line in this study. All *C. sonorensis* used were 3–4 days old and had not been fed sucrose following emergence from pupae.

### DNA extraction

The genomes of *C. sonorensis* males and females were sequenced separately. Adults were separated by sex under a stereomicroscope and stored in 95% ethanol at room temperature prior to use. In order to obtain sufficient amount of DNA for genomic sequencing, DNA was extracted from 375 males in three pools of 100 and one pool of 75 individuals, and from 150 females in one pool of 100 and one pool of 50 individuals. Pooled samples were homogenised twice for 30 s in 75 μl of phosphate buffered saline using a Tissuelyser™ (Qiagen, UK) at a frequency of 25 Hz/s and a 3 mm stainless-steel ball bearing (DeJay Distribution Ltd., UK) within 2 ml screw-topped tubes. Genomic DNA extraction was conducted using gravity-flow anion-exchange tips (Qiagen’s Genomics Tip 20/G) with extraction of DNA up to 150 kb in size and a maximum of 20 μg of product. A user-developed protocol for mosquitoes and other insects was followed (see Additional file 1: Supplementary Methods). The DNA obtained from each extraction was pooled by sex and the concentration of the resulting samples was evaluated using a Qubit® 2.0 Fluorometer (ThermoFisher Scientific, UK) with the Qubit® DNA BR Assay Kit (ThermoFisher Scientific, UK). The integrity of the genomic DNA was visualized using a 5 μl sample on a 1% (weight/volume *w/v*) agarose gel.

### Genome sequencing and de novo assembly

8–9 μg of high molecular weight (HMW) genomic DNA were used to sequence the genome of male and female *C. sonorensis* separately. One paired-end (PE) libraries of insert sizes 200 bp a mate-pair (MP) library with an average fragment size of 4.4 kb were sequenced with 150 bp paired-end from both female and male pools were run on Illumina HiSeq 2000 and 2500 sequencing systems. Library construction and sequencing was performed at The Earlham Institute (Norwich, UK). Read pair quality was assessed with FastQC v0.10.1. Results suggested that 6 bp should be removed from the start of each read, and in the case of MP data the last 40–50 bp had unexpectedly high levels of k-mer duplication. Both PE and MP reads were filtered to limit their size to 100 bp after removal of the first 6 bp. MP data was processed with NextClip v1.0 [30] to select reads which contained both ends of the original fragment. This created a dataset of reads with variable length, which were subsequently limited to 100 bp.

**Fig. 1:**
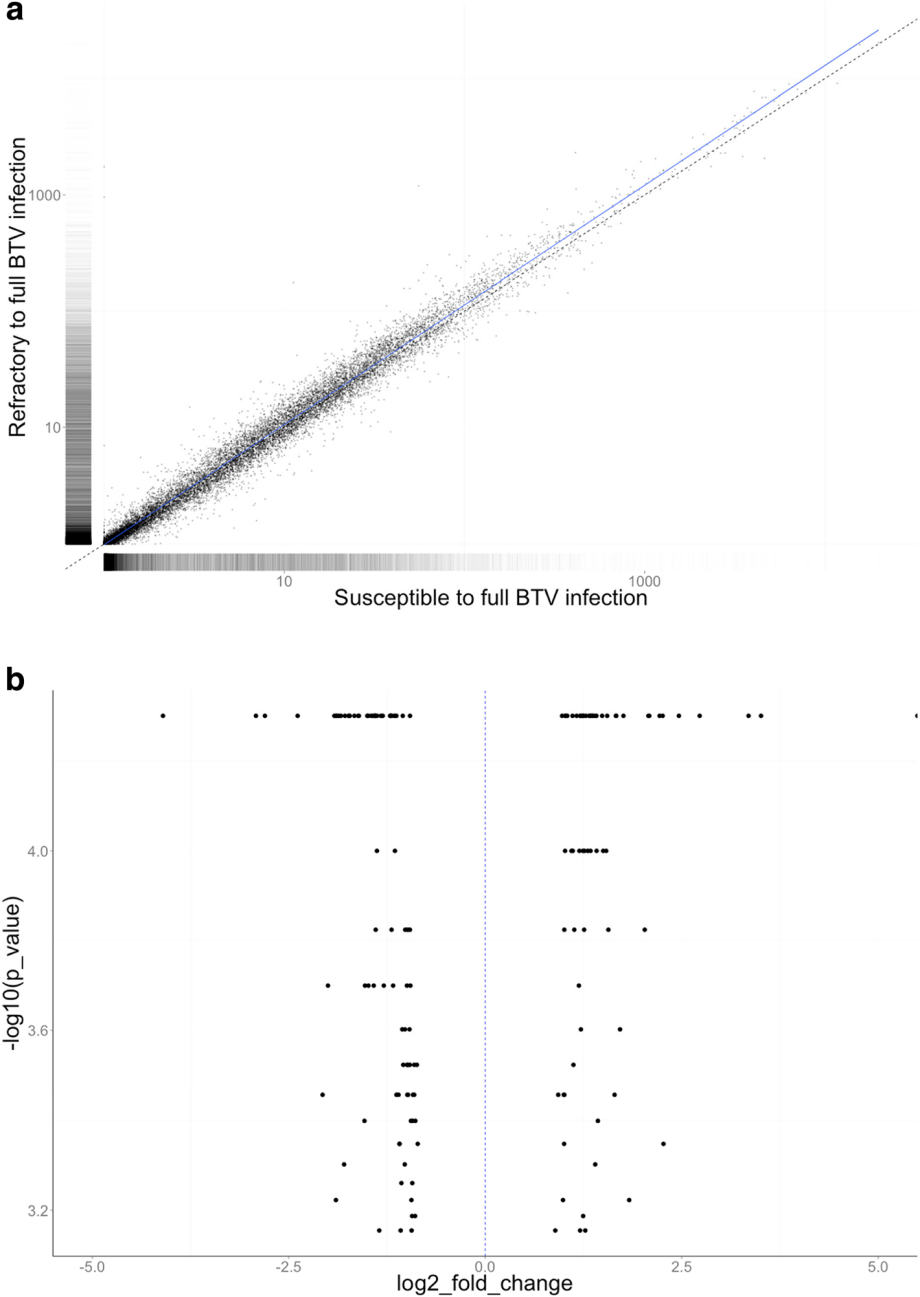
Differential gene expression analyses between females susceptible to full BTV infection and refractory females. **a** Scatterplot of the pairwise comparison of the gene expression levels between the two phenotypes. The average gene expression across all genes is displayed by the blue line. **b** Volcano plot displaying fold changes in expression of the 165 differentially expressed genes between *Culicoides sonorensis* that are susceptible to infection and those that are refractory to full infection with BTV

PE and MP libraries were initially assembled using Velvet v1.2.08 [31] with a k-mer length of 91. The N50 of the assembly was maximized using an expected coverage of 27, a coverage cut-off of six, and with scaffolding option set to ‘no’. This initial assembly was filtered to remove short (< 4 kb) and repeated contigs (> 90% sequence identity to a larger contig) using BLAT v34 and a custom script to count identical bases, considering any overlaps between matches. The assembly was then scaffolded using SSPACE v2.0 [32] and the 400 bp PE library, using three passes of decreasing stringency. Any remaining contigs smaller than 500 bp were removed (9913 total). The resulting assembly was then scaffolded a second time using the 4.4 kb MP library with SSPACE v2.0 (Fig. 1). This assembly was assessed using FRCbam v1.0 [33] and Reapr v1.0.15 [34], and the output from Reapr was processed with GapFiller v1.11 [35]. The final assembly was assessed using FRCbam v1.0 to confirm that an improvement in the reported error rate had occurred. Genome size was estimated using the number of total trimmed nucleotides which were used in the assembly divided by the maximum of the per-position coverage frequency distribution, after reads were mapped back to the assembly using BWA-MEM [36]. Kmer spectra were assessed using KAT (https://kat.readthedocs.io/en/latest). Redundant contigs were identified using Redundans [37].

Data (reads, assembly and annotation) have been deposited in the ENA database under the accession number PRJEB19938. The genome used in the subsequent analyses is the latest assembly without redundancy removal.

### Gene model prediction and annotation

Prediction and annotation of the genome assembly was conducted using MAKER v2.31.6 [38]. The genome annotation was carried out using the transcriptome data reported in this study and transcriptome data from previous studies on *C. sonorensis* [17, 39]. In addition, we used the genome annotations of ten species of *Anopheles*, *Ae. aegypti, Belgica antarctica* Jacobs*, Culex quinquefasciatus* Say*, Drosophila melanogaster* Mg., *Lutzomyia longipalpis* (Lutz & Neiva, 1912) and *Phlebotomus papatasi* (Scopoli) (see Additional file 1: Supplementary Methods for the species and genome assembly versions used). SNAP v2006-7-28 [40] and AUGUSTUS v2.5.5 [41] were used to predict ab initio gene models. SNAP v2006-7-28 was trained using 500 models and AUGUSTUS v2.5.5 using the best 1000 models extracted from the initial MAKER v2.31.6 output, as recommended in the software documentation. Coverage of conserved proteins was assessed using the CEGMA v2.4 [42] and BUSCO v3.0.2 [43] pipelines. Analysis of Gene Ontology annotation was performed with Blast2GO v3 [44]. The orthology of the annotated genes was established using the Ensembl Compara Gene Trees pipeline [45], using the genomes of *C. sonorensis* and 17 other arthropod species as input (see Additional file 1: Supplementary Methods). Genes were functionally annotated using InterProScan, which computationally identifies the presence of domain, motif or family signatures within a protein sequence and infers functional descriptors (taken from the Gene Ontology (GO)).

### Infection experiments with bluetongue virus

The virus used to infect *C. sonorensis* was a western topotype strain of BTV serotype 1 (GIB2007/01) [46]. Virus stock was mixed 1:1 with defibrinated horse blood (TCS Biosciences, Buckingham, UK) with 6.2 Log_10_ (TCID_50_)/ml being the final infectious dose. All blood-feeding of *C. sonorensis* was conducted using a Hemotek membrane-based system (Discovery Workshops, Accrington, UK). A total of 150 and 145 *C. sonorensis* in two replicates were fed on the blood:BTV-1 suspension and survived the extrinsic incubation period of 8 days at 25 °C. During this period of incubation, 10% (*w/v*) sucrose solution was offered via a cotton wool pad. Each individual was then decapitated using disposable needles and the head and remainder of the body of each were stored separately in RNAlater™ (Thermofisher Scientific, UK) at − 20 °C.

### RNA extraction

RNA extraction from *C. sonorensis* was performed following the TRIzol® (ThermoFisher Scientific, UK) protocol (see Additional file 1: Supplementary Methods). In brief, samples were homogenised in 100 μl of Schneider’s *Drosophila* media (Gibco™, Thermofisher Scientific, UK) and RNA was extracted using TRIzol® and chloroform, followed by a precipitation with isopropanol. The integrity of the RNA was analysed using a Bioanalyser 2100 and the concentration was estimated on a Qubit® 2.0 Fluorometer (ThermoFisher Scientific, UK) with the Qubit® DNA BR Assay Kit (ThermoFisher Scientific, UK).

RNA was extracted from individual heads of *C. sonorensis* fed with blood:BTV-1 as described for the bodies (Additional file 1: Supplementary Methods). The corresponding bodies (abdomen and thorax) of the BTV-1-positive and BTV-1-negative heads were pooled separately for RNA extraction. In addition, the RNA from 100 *C. sonorensis* fed on horse blood 3 days post-emergence and left without access to sucrose for 8 days, and from 100 *C. sonorensis* fed on a 10% (*w/v*) sucrose solution from 3 to 8 days following emergence, were also extracted and their transcriptomes sequenced as controls.

### Quantification of infection with BTV-1

We used RT-qPCR to detect and quantify the presence of disseminated BTV-1 viral RNA in the heads of blood:BTV-1 fed *C. sonorensis*. For this, the RNA was first reverse-transcribed into DNA using the ProtoScript II First Strand cDNA Synthesis kit (New England Biolabs) (see Additional file 1: Supplementary Methods for a detailed protocol). A reaction with no enzyme was included as a no-RT negative control. The resulting cDNA was used as template in RT-qPCRs for the detection of BTV using SYBR Green assays (Additional file 1: Supplementary Methods). The reactions were performed using the BTV specific primers BTVuni 291-311F 5’ GCTTTTGAGGTGTACGTGAAC 3′ and BTVuni 381-357R 5’ TCTCCCTTGAAACTCTATAATTACG 3′ [47]. No-template and no-RT reactions were included as negative controls. To verify that negative reactions were not due to a lack of cDNA in the sample, we also performed RT-qPCR reactions for each sample using *C. sonorensis* specific primers for the Vacuolar ATPase gene (Vac-ATPase forward 5’ GCTGCTGCTGCCATCATTTT 3′ and Vac-ATPase reverse 5’ CCGGTCGCATCACTGACATA 3′). All reactions were performed in duplicate. Samples with and without BTV-1 RNA were identified using the quantification cycle (C_q_) values of the reactions.

For the purposes of this study, we considered *vector competent* individuals to be those with disseminated infections that include replication in the head capsule, as this has been demonstrated to allow the isolation of BTV-1 [46]. We refer as *refractory individuals* to those without detectable BTV after a suitable incubation period (8 days), those with BTV midgut infections that had not disseminated within the insect, those where the process of dissemination had not been completed (i.e. those with haemocel dissemination barriers, midgut escape and infection barriers) and those still retaining inactivated BTV following the infective bloodmeal [24]. In the case of this study, we used a C_q_ value of 27 to differentiate between vector competent and vector refractory (see Results).

### Transcriptome sequencing and analyses

In total, eight transcriptomes were sequenced: two biological replicates each for i) BTV-competent; ii) BTV-refractory; iii) blood-fed; and, iv) sucrose-fed. Library construction and sequencing was performed at Edinburgh Genomics (The University of Edinburgh, Scotland). A total of 2.5 μg of RNA from each sample was used to construct TrueSeq libraries, which were sequenced using 50 base paired-end (PE) reads on an Illumina HiSeq 2500. Each library was run in two lanes.

The quality of the raw sequence data was analyzed in FastQC v0.11.2. Reads were aligned to the genome assembly (the version of the genome that has not been cleaned of the redundant scaffolds) with TopHat v2.0.6 [48] using the gene model annotations generated from the genome as reference. The resulting bam files were assembled with Cufflinks v2.2.1 [49] and merged into a single transcriptome with Cuffmerge v2.2.1. Quantification of gene and transcript expression and comparison of the expression levels was performed with Cuffquant v2.2.1 and Cuffdiff v2.2.1, respectively. The Cuffquant results for each transcriptome (two biological + two sequencing lane runs) were grouped per experimental conditions (blood-fed, sucrose-fed, vector competent and vector refractory) to compare the expression profiles between these different experimental conditions with Cuffdiff. Differential expression analyses were then explored with the R package CummeRbund v2.9.3 [50]. Differentially expressed (DE) genes with a significant change in expression level were identified using an *α* value of 0.05, which establishes the filtering value of the multiple-testing corrected *q*-values. Identification and functional classification of the DE genes was carried out using BLAST and InterProScan searches in Blast2Go version 4.0.2. Enrichment and gene set enrichment analyses were performed on the sets of DE genes between the different conditions using the interface provided in Blast2Go to the Fisher’s Exact Test implemented in FatiGO [51] and the Gene Set Enrichment Analysis (GSEA) package [52]. To perform the GSEA, all genes were ranked per the logarithmic fold-change, removing the infinite values. Transcriptome data have been deposited in the ENA database under the accession numbers ERR2171964-ERR2171979.

### Validation of differentially expressed genes

Quantitative reverse transcription PCR (RT-qPCR) was used to validate the change in expression of four differentially expressed (DE) genes between the vector competent and refractory pools of *C. sonorensis*. These DE genes were selected based on previous identification in vector competence studies of *C. sonorensis* or because they have a direct functional link with antiviral response. The genes were: a gene sharing 73% identity with the antiviral helicase *ski2* of *Cx. quinquefasciatus* (*ski2*; XP_001845019); a gene sharing 63% identity with *glutathione S-transferase* (*gst*; XP_001654620), a gene sharing 96% identity with *glutathione S-transferase-1* from *C. sonorensis* (*gst-1*; AAB94639) and a gene sharing 49% identity with a gene encoding a *Toll* protein in *Acyrthosiphon pisum* (Hempitera: Aphididae) (XP_001948700). The RNA used to validate the change in expression were the two biological replicas of vector competent and refractory *C. sonorensis* transcriptomes sequenced.

Expression levels were normalized against three reference genes whose stability was ranked using three approaches, BestKeeper [53], geNorm [54], and NormFinder [55] (Additional file 2: Table S3). Gene-specific primers and probes were designed using Primer3Plus [56] (Additional file 2: Table S4; Additional file 1: Supplementary Methods). Primers were compared against the *C. sonorensis* genome using BLAST to ensure specificity to the target region. Amplification efficiency was tested by RT-qPCR with hydrolysis probes by generating a standard curve using triplicate technical replicates of five-fold serial dilutions of RNA template from teneral female *C. sonorensis* specimens. All RNA templates were treated with Turbo DNA-Free™ Kit (ThermoFisher Scientific, UK) prior to RT-qPCR to remove any DNA from the sample following the manufacture recommended protocol. Starting template total RNA concentration was evaluated using a Qubit® 3.0 Fluorometer (ThermoFisher Scientific, UK) with the Qubit® RNA HS Assay Kit (ThermoFisher Scientific, UK).

cDNA synthesis and quantitative amplification was performed in one reaction using the Superscript® III One-Step RT-qPCR System with Platinum® Taq DNA polymerase (ThermoFisher Scientific, UK) (see Additional file 1: Supplementary Methods for a detailed protocol). Reactions were performed following the fast cycling programme as described in the manual (Additional file 1: Supplementary Methods). PCR amplification efficiency of each primer-probe-target combinations was calculated via the linear regression of C_q_ as the log2 of the relative RNA template concentration using the ggplot2 package [57] in R. Reactions which exhibited amplification efficiency between 90 and 110% with an R^2^ value of ≥0.98 were considered valid. Relative expression levels for the gene of interest between vector competent and refractory *C. sonorensis* were calculated using the ΔΔCq method [58].

### Molecular evolution analyses

Homologues of the *ski2* and *gst-1* genes in other insect species were identified using BLAST in Blast2Go. The top BLAST hits from *Ae. aegypti*, *Cx. quinquefasciatus* or *An. gambiae* were then used to identify the orthologues in other vector species in VectorBase [59]. We verified that the sequences used started with Methionine and ended with a stop codon, whenever possible. The protein sequences identified were aligned using the mode expresso of T-Coffee [60]. Poorly aligned regions (positions with score 0–6) were trimmed from the alignment and the resulting alignments were used in the evolutionary analyses (see Additional file 1: Supplementary Methods for more details and commands used to align the sequences; Additional file 3: Supplementary Alignment which include the sequences before and after alignment in fasta format). Genetic distances and the diversity were estimated using the software MEGA v7.0.26 [61].Phylogenetic analyses were performed in MrBayes v3.2 [62] using the protein alignment as input and the models of evolution that best fitted our alignments were identified using ProtTest 3.4.2 [63]. Two independent analyses of 2 million generations with four chains (one cold and three heated) were conducted. Trees and parameters were sampled every 50th generation and the first 10,000 were discarded. The remaining trees were used to estimate the consensus and Bayesian posterior probabilities of each branch. The s*ki2* from *Saccharomyces cerevisiae* Meyen ex E.C. Hansen and the *gst-1* from *Pediculus humanus* L. were used as outgroups.

## Results

### Genome sequencing, construction and annotation

Assembly of the Illumina reads resulted in a genome of 189,075,353 bp (189 Mb) assembled in 7974 scaffolds. In contrast, the total genome size estimated using the method of Schell et al. [36] (from the maximum depth of read coverage) was 304.74 Mb (Additional file 2: Table S11). The N50 of the genome assembly was 89,502 and the proportion of Ns was of 2.63% (Table 1). Analysis with RepeatModeler [64] identified a total of 793 repeat regions encompassing ~ 14% of the genome. When low-complexity regions are included, the total repeat content is 29.7% including 2 Mb of Type II transposons and 5 Mb of Type I transposons (mostly LINE elements). The GC content of the *C. sonorensis* genome is 28% in the complete assembly and 34% in the exons (Table 1). This is low when compared to the closest fully sequenced relative to *C. sonorensis* (*Belgica antarctica* (Diptera: Chironomidae): total GC content = 39%) and a range of other Diptera genomes (Table 1).

**Table 1:** A comparison of genome characteristics of the *C. sonorensis* genome with other selected Diptera species

Parameter	*Culicoides sonorensis*	*Aedes aegypti* L3	*Aedes aegypti* L5^a^	*Anopheles gambiae* AGAM P4^a^	*Drosophila melanogaster* BDGP6^a^	*Belgica antarctica* GCA_000775305.1
Genome size (Mb)	189	1384.1	1278.7	273.1	143.7	89.7
Number of contigs	15,810	36,205	2539	16,825	2442	22,492
Contig N50	30,774	82,500	11,758,062	85,555	19,488,218	13,551
Contig N90	5567	15.283	74,389	5600	666,663	1865
Number of scaffolds	7974	4757	2310	8	1870	4997
Scaffold N50	89,077	1,547,048	409,777,670	49,364,325	25,286,936	98,164
Scaffold N90	14,215	324,062	83,687	2684	23,513,712	18,418
Ns (%)	2.8	5.3	0.0	7.6	0.8	0.9
Maximum contig length	552,397	685,587	71,953,859	808,130	27,905,041	131,402
Repeats + low complexity regions	29.7%	73%	78%	25%	23%	4%
GC content	28.4%	38.3%	38.2%	44.3%	42.0%	38.9%
GC exons	34.3%	46.8%	46.2%	54.6%	49.1%	47.1%
Protein-coding genes	15,612	15,796	14,626	13,075	13,918	13,510
Mean protein-coding gene length	5040 bp	18,126 bp	46,920 bp	6442 bp	6960 bp	2555 bp
Mean exon length (protein coding genes)	480 bp	460 bp	494 bp	438 bp	538 bp	325 bp
Mean intron length (protein-coding genes)	632 bp	23,265 bp	10,456 bp	1008 bp	1147 bp	213 bp
Mean introns per transcript (in protein-coding genes)	6.7	5.1	7.9	5.5	6.9	5.3
Maximum splice isoforms/protein-coding gene	19	20	50	20	75	n.a.

^a^in the case of these species, the number of scaffolds shown correspond to the chromosomal arms plus minor scaffolds that may have been mapped but not assembled

Analyses on the completeness of the assembly identified 98.79% (100% including partial matches) of 248 Core Eukaryotic Genes Mapping Approach (CEGMA) genes and over 97% of the Benchmarking Universal Single-Copy Orthologs (BUSCO) gene set for Insecta (97.1%), Arthropoda (97.7%) and Metazoa (97.3%), with additional genes from these sets (0.7–0.9%) found in fragmentary form. Of these genes, 65.5% (Metazoa)-66.3% (Insecta, Arthropoda) were found in single copy, compared with 89.3% of Insecta BUSCO genes that were present in single copy in the *Aedes aegypti* assembly, and a higher number for some other assemblies (Additional file 2: Table S12). There is a duplication level of 30.8% within the *Culicoides* genome assembly, which is high in comparison to other genomes. This could be the result of genetic variation among/within the sequenced genomes from the pool of individuals and the representation within the assembly of alternative alleles.

Annotation of the Illumina assembled genome resulted in 15,612 protein-coding genes and 21,336 transcripts, which is comparable to the number of protein-coding genes in other species of Diptera (Table 1). The mean gene, intron and exon lengths in *C. sonorensis* are 5040 bp, 828 bp, and 480 bp, respectively. The average number of introns per gene in *C. sonorensis* is 6.7. A total of 2991 genes have been estimated to be alternatively spliced and the maximum number of splice variants per gene (19) is comparable to *Ae. aegypti* and *An. gambiae*.

To assess redundancy, we generated the K-mer spectra of the assembly and removed potentially redundant contigs using Redundans. This generated an alternative assembly of 156 Mb, with 3839 contigs, and an N50 scaffold length of 109,184 bp. Coverage of the BUSCO data sets was not changed, with values for Insecta, Arthropoda, Metazoa and Eukaryota within +/− 0.1% of the original assembly. The proportion of duplicate BUSCOs, however, was reduced from 30.8% (unreduced Illumina assembly) to 12.1–13.2% (reduced assembly) (Table 2), confirming the presence of BUSCO gene family members within the redundant contigs. Part of the redundancy occurs in repetitive elements of the genome, including satellite, simple and tandem repeats, transposons, LTRs and low complexity regions, which are reduced by approximately 20% after analyses. In addition, 20% of the genes are affected by redundancy, and the number of genes after the redundancy is collapsed becomes 12,453. However, of the 3159 gene models that are removed, 80% are still present in the Redundans-reduced assembly and thus are likely to represent miss-assemblies due to heterozygosity within the sequencing material. Of those genes reported in this study, 62 were affected by redundancy but only 2 did not have a high confidence BLAST hit in the reduced genome assembly.

**Table 2:** Comparison of the original, and redundancy-reduced, assemblies of *C. sonorensis*

	Original assembly		Assembly after removal of putative redundant contigs	
Assembly size (Mb)	189		156	
Number of scaffolds	7974		3839	
Scaffold N50	87,872		109,184	
BUSCO coverage %	Complete	Duplicate	Complete	Duplicate
Insecta	97.1	30.8	96.9	12.2
Arthropoda	97.7	31.4	97.5	12.5
Metazoa	97.3	31.8	92.2	12.1
Eukaryota	98.6	36.6	98.7	13.2

Homology to genes annotated in other species was detected for 93% of the genes annotated in *C. sonorensis*, with an average of 19 homologous genes and nine orthologues identified per gene; these numbers are comparable to the other species used in the analysis. Over 90% of the *C. sonorensis* genes were linked to their homologues via the ancestral nodes of Arthropoda (6609 genes), Neoptera (6361 genes) or Diptera (1225 genes).

InterProScan was used to detect protein domains and assign GO terms to genes. Overall, a similar number of domains and high-level GO terms were associated with at least one gene as in most other sequenced insects (Additional file 2: Table S13), although a larger number of specific GO terms tend to have been applied in genomes that have been manually annotated for gene function. In total, 46,329 GO term assignments were made. This compares to 45,600 assignments for *An. gambiae*, and 103,897 assignments for *D. melanogaster* (in which many terms have been manually assigned based on the literature, in addition to automatic assignments). Of 149 high-level categories in the GO (“GO Slim”), 137 were present in *C. sonorensis* as opposed to 140 categories represented in the genome of *An. gambiae* and 144 in *D. melanogaster*. GO terms assigned to more genes in *C. sonorensis* than either *An. gambiae* and *D. melanogaster* include: ion binding (2869 genes, as opposed to 2543 in *An. gambiae* and 2349 in *D. melanogaster*), oxidoreductase activity (703 genes, as opposed to 622 in *An. gambiae* and 632 in *D. melanogaster*), kinase activity (367 genes as opposed to 315 in *An. gambiae* and 363 in *D. melanogaster*), hydrolase activity acting on glycosyl bonds (112 genes as opposed to 105 in *An. gambiae* and 107 in *D. melanogaster*) and tRNA metabolic process (101 genes as opposed to 86 in *An. gambiae* and *D. melanogaster*). These results suggest an increase in the number of genes encoding these functions in *C. sonorensis* compared with other species, although the possibility that such genes are selectively over-represented in the current *C. sonorensis* assembly cannot be ruled out.

To identify tandemly arrayed genes, we looked for genes that contained common domain architectures (as defined by the set of InterPro-defined domains found within each gene) and were located within 100,000 nucleotides of each other. One thousand, two hundred and eighty one genes were found in such arrays. The longest such array contained 10 copies of a gene containing a chitin-binding domain. The longest array of a protein with this architecture comprises just two copies in *D. melanogaster*, and there are no such arrays in *Ae. aegypti* or *An. gambiae*, although there are various longer arrays of proteins containing this domain alongside others in these species (Additional file 2: Table S14). The second longest such array contains 9 copies of a gene encoding a protein with arrestin and immunoglobulin domains; the homologous proteins are found in 10 tandem copies in *Ae. aegypti* and *D. melanogaster*, and four tandem copies in *An. gambiae* (Additional file 2: Table S14).

### Transcriptome sequencing and analysis

Paired-end sequencing of transcriptomes resulted in an average of 50 million reads with a length of 50 bp for each sample sequenced. Phred scores exceeded 34 in all cases. The transcriptomes from the different conditions were merged with Cuffmerge prior to a comparative analysis of their gene expression profiles. The number of genes on the merged transcriptome is 17,263 and the number of transcripts is 35,813. Of the genes present in the transcriptome, 15,630 had already been annotated in the genome. One thousand, eight hundred and sixteen transcripts, mapping to 1635 loci, were novel. The proportion of gene loci with a single transcript is 62.7% (10,826), but 93.3% among the genes that had not been annotated on the genome. BLAST searches against NCBI’s Arthropod non-redundant (nr) protein database resulted in 92.4% of the isoforms (33,095) and 90% of the genes matching to a database entry (15,268 of the genes had one or more hits).

### Differential expression of genes between competent and refractory *Culicoides sonorensis*

The RT-qPCR for the detection of BTV RNA in individual heads identified 85 (56%) *C. sonorensis* in replicate one and 53 (37%) in replicate two that produced C_q_ values ≤27 (Additional file 4: Figure S1). We used the C_q_ value 27 as a cut off for differentiating between vector competent and refractory individuals as the distribution of values was bimodal and 27 was the local minimum (Additional file 4: Figure S1). Sixty-five (44%) *C. sonorensis* in replicate one and 92 (63%) in replicate two produced C_q_ values of > 27 or no C_q_ value. Replicate one had a significantly greater proportion of disseminated infections than replicate two (Fishers exact test; df = 1, *p* = 0.0007). Three and nine individuals from the first and second experiment replicates, respectively, were false negatives, that is, samples that were negative for BTV-1 but that also failed to amplify the *C. sonorensis* genes. This indicated that the RNA extraction failed and were discarded from further analyses. Transcriptome studies were therefore conducted using 147 and 136 female *C. sonorensis*, from the two biological replicates of the infection study, respectively.

Gene expression profiles showed that more genes had their expression upregulated in refractory *C. sonorensis* than in competent individuals (Fig. 1a). A total of 165 genes demonstrated significant differential expression (DE) (Fig. 1b; Additional file 2: Table S1) of which 123 (75%) had been annotated and 42 (25%) represented novel genes (Additional file 2: Table S2). A total of 94 (57%) were upregulated in refractory individuals, while 71 were downregulated (43%) (Additional file 2: Table S1). One hundred and twenty of the 165 genes showing DE had one or more BLAST hits in the non-redundant (nr) protein database from NCBI (Additional file 2: Table S2) and 88% of these corresponded closely to genes from other members of the suborder Nematocera. Of the 165 genes, 63 were associated with gene ontology (GO) terms (Additional file 4: Figure S2). However, enrichment analysis showed no significant results.

**Fig. 2:**
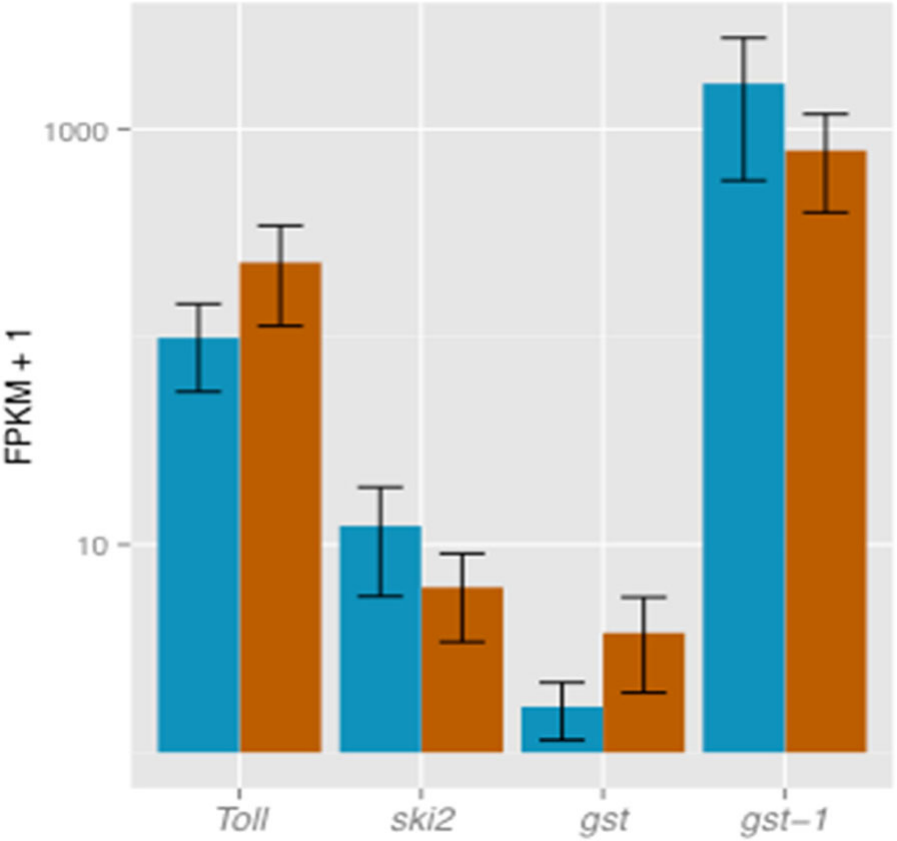
Expression bar plots for candidate genes identified as differentially expressed at significant level between vector competent (orange bars) and refractory (blue bars) samples to full BTV infection. *Toll* corresponds to the *Toll*-like gene, *ski2* is the antiviral helicase *ski2*, *gst* is *glutathione s transferase*, and *gst-1* is the *glutathione s transferase-1*

*Ski2* (XLOC_006435) and *glutathione S-transferase-1* (CSON011559) showed an increase in expression levels in the females that were resistant to full dissemination, the log2 fold change being − 1.10 (*P* = 0.00035; *q*-value = 0.00381) and − 1.07 (*P* = 0.00055, q-value = 0.00555), respectively (Fig. 2; Additional file 2: Table S1). In contrast, the *Toll*-like (XLOC_000522) gene and the *glutathione s transferase* (CSON010973) were expressed at higher levels in the samples that were susceptible to a fully disseminated BTV infection (log2 fold change 1.21, *P* = 0.00005 and *q*-value = 0.00072) and (log2 fold change 2.07, *P* = 0.00005 and *q*-value = 0.00072) (Fig. 2; Additional file 2: Table S1).

The direction and degree of change in expression levels of the four genes identified as potentially influencing *C. sonorensis* competence for BTV (*Ski2*, *gst-1*, *gst* and *Toll*-like) was confirmed using RT-qPCR and the same samples. The reference genes used were *CytB5*, R*pL13* and *RpS8*, which showed the highest level of stability of those tested (Additional file 2: Tables S3 and S4). The direction of up and down regulation of the genes and the log-2 fold change was similar in all cases as that detected in the RNAseq data (Additional file 2: Table S5).

### Molecular evolution of *ski2 antiviral helicase* and *glutathione s transferase-1*

**Fig. 3:**
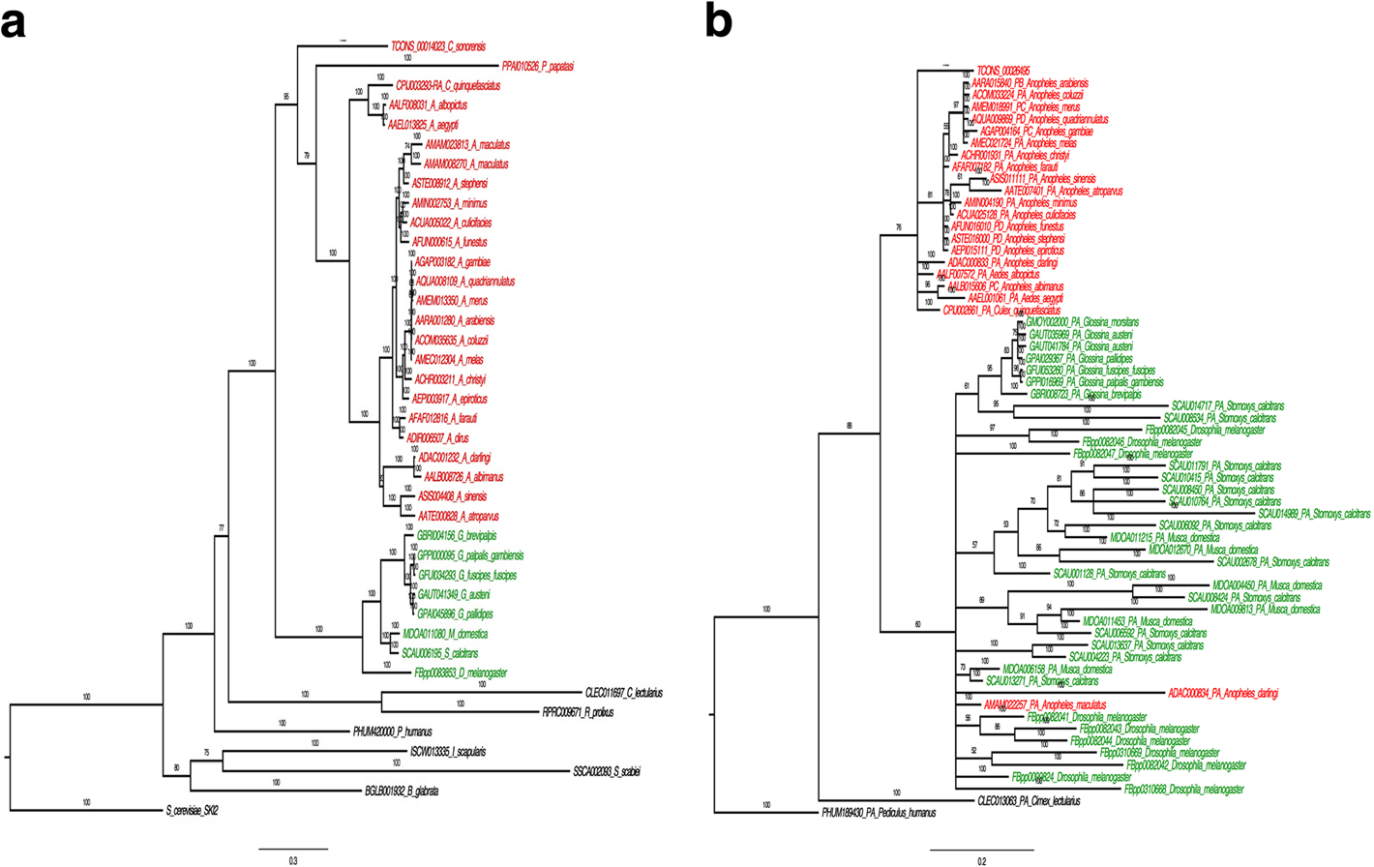
Bayesian phylogenies of *ski2* (**a**) and *gst-1* (**b**) genes. Tip labels correspond to the Ensembl Identifier followed by the species name (in the case of *C. sonorensis*, the number that precedes correspond to the RNA transcript); colouring corresponds to the Nematocera in red and Brachycera in green. Labels on branches show the posterior probability values

The top BLAST hit for the *C. sonorensis ski2* was the gene from *Cx. quinquefasciatus* (XP_001845019.1 = CPIJ003293). There are 37 genes identified as orthologous to *Cx. quinquefasciatus ski2* in 36 species in VectorBase. Thirty five are 1-to-1 orthologues and there are two orthologous genes in *An. maculatus* (Additional file 2: Table S6a), although these two orthologues align each to the 5′- and 3′-ends of the *Ski2* gene, suggesting that the *Ski2* gene model is split in two scaffolds in this species. The mean evolutionary diversity estimated using JTT model with gamma distribution value α = 0.5 (as estimated with ProtTest) and pairwise deletion across the sequences from Diptera species (no outgroups included) was 0.513, and pairwise distances between protein sequences ranged between 0.000 (*An. coluzzi* v. *An. gambiae*) and 1.198 (*G. fuscipes fuscipes* v. *An. maculatus*). The distance of the ski2 protein between *C. sonorensis* and mosquitoes was 0.752, with sandflies was 0.834, and with Brachycera had a mean distance of 0.928. The mean evolutionary distance within the Nematocera was 0.272, and 0.171 within the Brachycera. The evolutionary relationship of this protein is consistent with species-level phylogeny (Fig. 3a).

In the *gst-1* gene from *C. sonorensis*, the most similar BLAST hit is that of *C. variipennis* (synonym of *C. sonorensis*; AAB94639.1) [25]. The second closest was the *gst-1* copy of *An. gambiae* (AGAP004164), for which 61 orthologues have been identified in 31 other species in VectorBase (Additional file 2: Table S6b). The mean evolutionary distance between the gst-1 protein of the Diptera species, estimated with the same model parameters as for ski2 protein, was 0.647. The mean distance between the gst-1 protein of *C. sonorensis* and that of mosquitoes was 0.199, while its evolutionary mean distance to Brachycera was 0.866. The pairwise distances ranged between 0.000 (observed between several sequences) and 2.914 (between the copy of *M. domestica* MDOA012670 and *An. coluzzi* ACOM0333224). The mean evolutionary distance within the Nematocera was 0.141, and 0.722 within the Brachycera. The phylogeny of the gst-1 protein recovers the two main clades in Diptera, Brachycera and Nematocera, but there is little resolution within these clades (Fig. 3b).

### Immune response related genes in *C. sonorensis*

All *D. melanogaster* genes from FlyBase that had InterPro hits containing Toll, Imd and JAK/STAT associated to their names and all GO terms matching to the Toll, Imd and JAK/STAT pathway terms were used as reference to identify the homologues in *C. sonorensis* (Additional file 2: Tables S7, and S8; Additional file 4: Figure S3). Of the 42 genes identified in the Toll pathway of *D. melanogaster* 36 were also identified in *C. sonorensis*; eight of the 11 genes of the Imd signalling pathway in *D. melanogaster* were identified in the *C. sonorensis* genome and 10 of the 13 *D. melanogaster* JAK/STAT pathway genes were identified. There are four instances in which the same *C. sonorensis* genes were identified as different immune-related genes. Two *C. sonorensis* genes, CSON012766 and CSON015181, were predicted by the Compara workflow to be homologous to both *Dorsal* and *Dif*. These two genes had 99.6% similarity at protein sequence level and 96% similarity at DNA sequence level, and are located in different scaffolds. The similarity to *D. melanogaster Dorsal* was 65% and to *Dif* was 61%. Four *C. sonorensis* genes, CSON001282, CSON001790, CSON007335, CSON011712, were homologous to both *Toll* (FBgn0262473) and *Tehao* (FBgn0026760). Blastp searches identified all four genes as having leucine-rich repeats and Toll/Interleukin-1 receptor homology (TIR) domains, and the similarity with *Toll* is about 38–40% at the protein sequence level. Two *C. sonorensis* genes, CSON003218, CSON008584, are homologous to *Peptidoglycan recognition protein LC* (Fbgn0035976) and *Peptidoglycan recognition protein LF* (Fbgn0035977). Using blastp searches, the two genes showed partial similarity higher than 50% to several PGRPs at the protein level, not being able to differentiate between them using BLAST. Finally, one single *C. sonorensis* gene (CSON004570) is homologous to Unpaired 1, 2 and 3 (FBgn0004956, FBgn0030904, FBgn0053542). Using blastp, the *C. sonorensis* protein had partial identities of 25–30% to these three Unpaired proteins of *D. melanogaster*. Similar to the cases above, it is not possible to identify the exact identity of the gene without further analyses.

Additional searches for immune related genes in *C. sonorensis* were carried out using blastp with *Ae. aegypti* and *Cx. quinquefasciatus* gene sequences from the ImmunoDB database (http://cegg.unige.ch/Insecta/immunodb) as queries (Additional file 2: Tables S9 and S10). Four annotated genes in *C. sonorensis* were identified as potential anti-microbial peptides (AMPs) belonging to the gene subfamilies Attacin, Cecropin, Defensin and Diptericin (Additional file 2: Table S9a); however, the analyses did not identify more than a single gene within each of the AMP subfamilies. Eight potential Toll-like receptors were identified using blastp although there were three instances in which the highest identities were different depending on the species used to provide input data (Additional file 2: Table S10), reflecting the large difference in sequence between the homologous genes in *Ae. aegypti* and *Cx. quinquefasciatus*. All five genes belonging to the Toll pathway (Additional file 2: Table S10) and all genes of the JAK/STAT signal transduction pathway were identified in *C. sonorensis* (Additional file 2: Table S10); while of the Imd pathway, one gene was identified in five of the eight subfamilies (Additional file 2: Table S10).

The Compara approach reconciles the gene tree (derived from sequence similarity metrics) with the species tree to identify likely orthologous relationships; but requires an initial clustering step (which can lead to the inclusion of sequences into the wrong trees) [45]. The only instance in which the two approaches identify a different *C. sonorensis* gene and the orthologue of a gene in the above pathways is that of *Pelle* (which activates the Toll Receptor), for which BLAST identifies CSON012655 as its homologue, and Compara identifies CSON013584 and CSON013585. There were other cases in which BLAST identifies a potential homologue while Compara does not (e.g. TNF-receptor-associated factor 6, Traf6, or tube) and genes Imd and Fadd, neither of the two approaches identify a homologue in *C. sonorensis*.

It should be noted that the results presented in this study come from automated pipelines. Although some cases have been manually verified, there are still genes which have not been identified in this study and other that need improvement in their annotation. It is expected that the annotation and curation of the genome will advance as it is used by the research community.

## Discussion

This study has produced the first de novo genome assembly of the Dipteran family Ceratopogonidae, which includes important vectors of emerging and re-emerging veterinary arboviruses [8, 65]. The closest relative to this group for which a genome of comparable quality is available is the non-biting midge, *Polypedilum vanderplanki* Hinton (Family: Chironomidae) [66], which diverged from the Ceratopogonidae some 220 million years ago. This study provides a primary resource for comparative studies with other arbovirus vector species (e.g. mosquitoes [67]). It also will facilitate efforts to produce transgenic *Culicoides* to control *Culicoides*-borne pathogens, although the limitations on colony production for the other epidemiologically relevant species of *Culicoides* remains a major constraining factor [14, 68].

The genome assembly of *C. sonorensis* has a similar size to that of other Diptera species for which their genome is available, except for *Ae. aegypti* which has an unusually large genome. There is however some discrepancy between the estimated size using the method of Schell et al. [36], the size of the original assembly and the size once redundancy is removed. The difference in estimated and assembled sizes could be due to the high level of redundancy as this will spread the coverage across different regions of the genome assembly that would be otherwise collapsed after redundancy removal. Thus, the overestimate of the size can be ultimately linked to the heterogeneity of the starting DNA material used for sequencing the genome. Various measures (the high proportion of BUSCO genes present in multiple copy, and estimates of genome size and redundancy using read mapping and alignment approaches) indicate the presence of a high level of redundancy in the assembly (Table 2), which could result from genetic variation within and amongst individuals used to sequence the genome. However, the individuals come from a colony that has not had any outbreeding for over 40 years, which should have reduced the genetic variation among the individuals of the colony. When the redundancy is collapsed the number of scaffolds is reduced by half, the genome size is reduced by 40 Mb and the N50 increases, resulting in a less fragmented genome. However, the coverage of BUSCO genes was not affected but the number of duplicate gene copies was reduced by more than 50%. In addition, 20% of the genes were removed after the redundancy analyses, although 80% still had a high confidence hit gene in the collapsed genome, indicating that they had been duplicated during the assembly. Of those reported in the present study, 62 were affected by redundancy but 60 still had a homologous gene in the collapsed assembly. In our view, the redundancy analysis can be considered as an analysis of confidence in the gene models of the original genome assembly, and there is high confidence in 80% of the genes annotated while 20% of the gene models should be considered as low confidence as they could be the result of assembly artefacts, miss-predicted genes, or gene families with several copies difficult to distinguish. This suggests that a proportion of the redundancy was due to heterozygosity. In addition, another part of the redundancy corresponded to repetitive elements of the genome, and these were reduced by 20% in the collapsed genome. The low GC content of the genome, which is the lowest of any Diptera species sequenced so far, is likely to have had an impact in the assembly of particularly AT-rich regions. Thus, the discrepancy between the genome sizes before and after the redundancy is removed can be partly explained by the heterogeneity of the starting material for sequencing affecting the gene prediction, but also partially due to naturally occurring repeat regions. Whether these genes and repeat elements are real or an artefact of the assembly process can only be verified with further experimental work. Long read-sequencing approaches would likely help reduce this redundancy.

The original genome assembly is not wholly contiguous, however, other general features are consistent with similarly sized genomes from other species. Thus, features such as the repeat content, gene count and level of alternative splicing are within the ranges observed in *An. gambiae* and *D. melanogaster*. Coverage of well-conserved gene families is complete and comparable with other published assemblies (Additional file 2: Table S12). The gene set has been annotated with a comparable number of InterPro domains and high-level GO terms as *An. gambiae* and other Diptera, while the number of specific GO terms is comparable to other genomes that have not been extensively manually annotated with functional descriptions. The molecular functions of ion binding, oxidoreductase activity, kinase activity, hydrolase activity acting on glycosyl bonds, methyltransferase activity all highly represented, as is the tRNA metabolic process. In addition, the genes of the immune-related pathways have been identified and annotated, revealing complete or nearly-complete orthologous pathways. Overall, all these observations indicate that the present assembly and annotation of the *C. sonorensis* genome represents a reliable resource to use in genetic studies of biting midges. Nevertheless, it is still highly fragmented and the use of long-read sequencing technologies like PacBio would help improving the scaffolding. In addition, the use of this genomic resource by the community will help improve the annotation of the genes. This is the case for many of the genomes of vector species that have been neglected over the years.

A striking feature of the *C. sonorensis* genome is its GC content, which is among the lowest reported for Dipteran species (although close to identical to the non-biting midge *P. vanderplanki* [66] and other non-Dipteran arthropods like the pea aphid, *Acyrthosiphon pisum* Harris [69]). This is not just a feature of the repeated content, but extends into the protein-coding genes (which have a higher GC content than the non-coding regions, but less than in comparable species: Table 1). Recent comparative analyses of codon usage in Diptera and Hymenoptera showed an association between codon bias and high GC content in Diptera, but low GC content in Hymenoptera [70]. Thus, the low GC content observed in *C. sonorensis* could reflect a codon bias in comparison to other Diptera species. Functional investigations of GC content have also revealed that it has an impact on genome functioning and species ecology in microbes [71], vertebrates [72] and plants [73]. It is also known that the GC content has an impact in the efficiency of sequencing technologies [74]. Regions with low GC content have less coverage than more GC balanced ones. This characteristic of the *C. sonorensis* genome should be accounted for in future genomic studies, and appropriate experimental designs that take into account the low GC content should be used (e.g. [75]).

Studies investigating gene expression during infection with BTV identified four candidate genes whose regulation was correlated with vector competence, despite only using a single time-point at 8 days post-infection and pools of *C. sonorensis* exhibiting different degrees of virus dissemination. Interestingly, the elevated expression of *gst-1* in refractory *Culicoides* observed in this study is consistent with a previously proposed genetic mechanism for *C. sonorensis* vector competence for BTV [13, 15, 24]. These studies identified a locus controlling vector competence in the AA colony, the origin colony of PIR-s-3 colony used in the present study. The former study used a maternally inherited 90kd protein consistent with genetic studies that suggested maternal inheritance of the competence controlling factor identified in unfertilized eggs of refractory females to isolate a clone that upon sequencing was identified as *gst-1* [25]. Though the current study evaluated gene expression after exposure to BTV and the previous study compared genetically selected resistant and susceptible families using individuals from these families who were not exposed to BTV, both independently identified involvement of *gst-1*. Thus, the differential expression of the gene in the current study is confirmation of its involvement in BTV infection in AA colony, although its role requires functional validation.

From the evolutionary perspective, the phylogenies of *ski2* and *gst-1* reflect the accepted evolutionary history of the included Diptera species, although the phylogenetic tree of *ski2* shows more resolution within Nematocera and Brachycera than the *gst-1*. It is also interesting to note the two contrasting modes of evolution of gst-1 between the two main clades of Diptera. The gst-1 protein in the Brachycera clade shows multiple duplications in comparison with the Nematocera and the estimated diversity in the Brachycera is higher (0.722) than that in the Nematocera (0.141). Within the Nematocera the level of diversity of the gst-1 protein is lower than in ski2.

Future studies of vector competence in *C. sonorensis* must characterize genes that facilitate BTV competence and differentiate these from differentially expressed genes that are the consequence of BTV infection. To determine variation in the mechanism and factors controlling BTV-vector competence in the AA and PIR-s-3 colonies observed in the present study, it will be necessary to carry out studies of different populations of *C. sonorensis* and other *Culicoides* vectors and non-vector species. Once controlling genes are identified it will be essential to assess the effect of polymorphisms, the various functions of the identified genes in the absence of the pathogen to evaluate factors that influence their frequencies, and compare these processes between different species of *Culicoides* [23].

The present paper presents the genome of *C. sonorensis* and its use in transcriptomic analyses to provide information that will help elucidate the transmission of viruses by vectors and provide new avenues of research to understand vector competence for BTV. We present a list of candidate genes that will be further explored to show their involvement in the transmission of BTV by *C. sonorensis*. Furthermore, the results also provide evidence for the involvement of genes belonging similar functional families in response to virus infection in different species belonging to different families of Diptera. These results show the importance of comparative analyses to interrogate the evolution of host-pathogen interactions, and the *C. sonorensis* genome will facilitate such studies.

## Conclusions

Here we present the first annotated genome of the biting midge, *C. sonorensis*, a vector of economically important viruses of livestock, such as bluetongue virus (BTV) and Schmallenberg virus. This genome has been fundamental in gaining information about the genetics of BTV transmission. Thus, gene expression comparison between females susceptible to a full infection by BTV and those females refractory to full BTV infection, identified 165 genes that are candidates to be involved in vector competence in this species. Of these genes, the *gst-1* gene was identified previously to be involved in BTV transmission, while the antiviral helicase *ski2* involved in suppressing the replication of dsRNA viruses. Overall, the publication of the genome and transcriptomes will lead to advances in the control of economically relevant arboviruses of livestock. In addition, this genome fills a phylogenetic gap of more than 200 million years, and will be a useful source of comparative data for studies of vector competence across Diptera.

## Additional files

Additional file 1: **Supplementary Methods**. DNA extraction. Protocol followed to extract DNA from *C. sonorensis* samples. Genome annotation and comparative analysis. Details about the genomes of species used in the gene annotation and in the comparative analyses. RNA extraction. Protocol used to extract RNA from *C. sonorensis*. RT-qPCR for the detection of BTV-1 infection. Description of the method used to detect the presence of BTV-1 in *C. sonorensis* females after the experimental infections. RT-qPCR protocol for the validation of differentially expressed genes. Description of the protocol used to verify the change in expression of *Toll-like protein*, *glutathione-s-transferase*, *glutathione-s-transferase 1* and *ski2 antiviral helicase* genes observed in the transcriptomes of vector-competent and refractory samples. Alignment of gst-1 and ski2. The commands used to align the protein sequences of gst-1 and ski2. These alignments were then used to run the phylogenetic analyses. (ZIP 117 kb)

Additional file 2: **Table S1.** Differentially expressed genes between vector competent and vector refractory *C. sonorensis* females. **Table S2.** BLAST results for the differentially expressed genes between vector competent and refractory transcriptomes. **Table S3.** Ranking of the expression stability of the reference genes used in RT-qPCR. **Table S4.** Primers and probe used for RT-qPCR of *ski2*, *gst*, *gst-1* and *Toll*-like. **Table S5.** Difference of expression levels in four genes (*Toll*-like, *gst*, *gst-1* and *ski2*) between vector-competent and vector-refractory females assessed by RNAseq and RT-qPCR. **Table S6.** Orthology analysis of two of genes *Ski2* **(a)** and *gst-1* **(b)**. **Table S7.** Immune pathways genes identified in *C. sonorensis* using the Ensembl Compara pipeline. **Table S8.** Homologue immune genes associated with Toll **(a)**, Imd **(b)** and Jak/Stat **(c)**. **Table S9.** Immune related genes identified in *C. sonorensis* using blastp. **Table S10.** Details of the blastp results. **Table S11.** Read mapping and estimate of assembly size according to the method of Schell et al... [36]. **Table S12.** BUSCO analysis results. **Table S13.** Number of distinct InterPro and GO terms annotated *C. sonorensis* and six other Diptera species. **Table S14.** Tandemly repeated gene arrays in *C. sonorensis* versus other insect species.

Additional file 3: **Supplementary Alignments**. Zip file containing the *gst-1* and *ski2* CDS and protein sequences, the protein alignment obtained using T-Coffee as described in the Additional file 1: Supplementary Methods, and the protein alignment after removing variable positions (with a T-Coffee value < 6 as described in the Additional file 1: Supplementary Methods). (DOCX 267 kb)

Additional file 4: **Figure S1.** Distribution of C_q_ values from RT-qPCR used to detect BTV-1 virus infection in the two virus-feeding experiments (in blue and orange) of *C. sonorensis*. The vertical, dashed line corresponds to a C_q_ value of 27 used to differentiate vector competent from refractory females. **Figure S2.** Classification and functional distribution of the genes differentially expressed between vector competent and refractory *C. sonorensis* per the Gene Ontology level 6. Blue: Molecular Function; Green: Cellular Component; Pink: Biological Process. **Figure S3.** Number of genes in the Toll (a), Imd (b) and Jak/Stat (c) pathways that have been duplicated or lost in different species of Diptera as identified using the Ensembl Compara pipeline. (DOCX 158 kb)

## Abbreviations

AMPs: Antimicrobial peptides
BLAST: Basic local alignment search tool
bp: Base pair
BTV: Bluetongue virus
BUSCO: Benchmarking Universal Single-Copy Orthologs
CEGMA: Core Eukaryotic Genes Mapping Approach
C_q_: Quantification cycle
DE: Differentially expressed
GO: Gene ontology
GSEA: Gene set enrichment analysis
gst: Glutathione S-transferase
HMW: High molecular weight
Imd: Immunodeficiency
JAK/STAT: Janus kinanses/signal transducer and activator of transcription
kb: Kilobase pairs
MP: Mate-pair
PE: Paired-end
RT-qPCR: Quantitative reverse transcription Polymerase Chain Reaction
TCID_50_: 50% Tissue culture infective dose
*w/v*: Weight/volume
μl: Microlitre
