# Supplementary material for: The genome of the biting midge Culicoides sonorensis and gene expression analyses of vector competence for bluetongue virus
Source: BMC Genomics. 2018 Aug 22;19:624. doi: 10.1186/s12864-018-5014-1 (PMC6106943; doi:10.1186/s12864-018-5014-1)
Supplement: Supplementary file 1 — Supplementary Methods. DNA extraction. Protocol followed to extract DNA from C. sonorensis samples. Genome annotation and comparative analysis. Details about the genomes of species used in the gene annotation and in the comparative analyses. RNA extraction. Protocol used to extract RNA from C. sonorensis. RT-qPCR for the detection of BTV-1 infection. Description of the method used to detect the presence of BTV-1 in C. sonorensis females after the experimental infections. RT-qPCR protocol for the validation of differentially expressed genes. Description of the protocol used to verify the change in expression of Toll-like protein, glutathione-s-transferase, glutathione-s-transferase 1 and ski2 antiviral helicase genes observed in the transcriptomes of vector-competent and refractory samples. Alignment of gst-1 and ski2. The commands used to align the protein sequences of gst-1 and ski2. These alignments were then used to run the phylogenetic analyses. (ZIP 117 kb) [file 12864_2018_5014_MOESM1_ESM.docx]

**SUPPLEMENTARY METHODS**

*DNA extraction*

The method is an adaptation of the user-developed protocol for the DNA extraction of mosquitoes and other insects using the QIAGEN Genomic tip 20/G. This protocol can be found online:

<https://www.qiagen.com/gb/resources/resourcedetail?id=b45c3cc3-7f2b-4f4a-aa37-21d814ed3730&lang=en>

1. A pool of *C. sonorensis* were homogenised in 75 µl of PBS twice for 30 seconds at 25 Hz/s with 3 mm stainless-steel beads, in a 2 mm screw-top microcentrifuge tube.
2. Add 1.8 ml of Lysis Buffer (20 mM EDTA, 100 mM NaCl, 1% Triton X-100, 500 mM Guanidine-HCl, 10 mM Tris pH 7.9) to the sample.
3. Add RNase A (20 µg/ml) and incubate the sample at 37^o^C for 30 mins.
4. Add 100 µl of Proteinase K and incubate at 50^o^C for 2-4 hours (until tissue is dissolved) with agitation.
5. Centrifuge the sample 20 minutes at 12-15,000 x *g*.
6. In the meantime, equilibrate the QIAGEN Genomic-tip 20/G with 1 ml of Buffer QBT and allowing the Buffer to flow through by gravity.
7. Transfer the supernatant lysate of the sample (from step 5) to the QBT-equilibrated Genomic-tip 20/G, and let it flow through the resin by gravity flow.
8. Wash 4 times the Genomic-tip 20/G with 1 ml of Buffer QC, allowing the buffer to flow through the Genomic-tip by gravity.
9. Elute the DNA with 800 µl of pre-warmed (50^o^C) Buffer QF, allowing the buffer to flow through by gravity.
10. Precipitate the DNA with 0.7 volumes (560 µl) of Isopropanol at room temperature.
11. Centrifuge for 20 minutes at maximum speed (~15,000 x *g*) to pellet DNA. Remove supernatant.
12. Wash the pellet with cold 70% Ethanol and centrifuge at maximum speed for 5-10 minutes. Remove supernatant and let the pellet dry.
13. Resuspend the pellet in 25 µl of nuclease free water.

*Genome Annotation and Comparative Analysis*

The table below indicates the sequence assembly version of each genome used in the genome annotation and comparative analysis. Both the identifiers assigned by the International Nucleotide Sequence Database Consortium (INSDC) to the assembly upon data submission, and those identifiers used by FlyBase and VectorBase, are given.

| **Species and strain** | **VectorBase/ FlyBase assembly identifier** | **INSDC assembly accession** | **Used in gene annotation** | **Used in comparative analysis** |
| --- | --- | --- | --- | --- |
| *Aedes aegypti* | AeagL3 | GCA_000004015.3 | Y | Y |
| *Anopheles albimanus* Wiedemann | AalbS2 | GCA_000349125.1 | Y | N |
| *Anopheles arabiensis* Patton | AaraD1 | GCA_000349185.1 | Y | N |
| *Anopheles christyi* Newstead & Carter | Achr1 | GCA_000349165.1 | Y | N |
| *Anopheles dirus* Peyton & Harrison | AdirW1 | GCA_000349145.1 | Y | N |
| *Anopheles epiroticus* Linton & Harbach | AepiE1 | GCA_000349105.1 | Y | N |
| *Anopheles funestus* Giles | AfunF1 | GCA_000349085.1 | Y | N |
| *Anopheles gambiae* PEST | AgamP4 | GCA_000005575.1 | Y | Y |
| *Anopheles minimus* Theobald | AminM1 | GCA_000349025.1 | Y | N |
| *Anopheles quadriannulatus* Say | AquaS1 | GCA_000349065.1 | Y | N |
| *Anopheles stephensi* Liston | SDA-400 | GCA_000300775.1 | Y | N |
| *Ayrthosiphon pisum* | N/A | GCA_000142985.2 | N | Y |
| *Belgica antarctica* Jacobs | N/A | GCA_000775305.1 | Y | Y |
| *Culex quinquefasciatus* Say | CXpipJ2 | GCA_000209185.1 | Y | Y |
| *Drosophila melanogaster* Mg. | BDGP6 | N/A | Y | Y |
| *Glossina morsitans* | GmorY1 | GCA_001077435.1 | N | Y |
| *Ixodes scapularis* | IscaW1 | GCA_000208615.1 | N | Y |
| *Lucilia cuprina* | N/A | GCA_001187945.1 | N | Y |
| *Lutzomyia longipalpis* | LlonJ1 | GCA_000265325.1 | Y | Y |
| *Musca domestica* | MdomA1 | GCA_000371365.1 | N | Y |
| *Pediculus humanus* | PhumU2 | GCA_000006295.1 | N | Y |
| *Phlebotomus papatasi* Scopoli | PpapI1 | GCA_000262795.1 | Y | Y |
| *Rhodnius prolixus* | RrpoC3 | GCA_000181055.3 | N | Y |
| *Sarcoptes scabiei* | SscaA1 | GCA_000828355.1 | N | Y |
| *Stomoxys calcitrans* | ScalU1 | GCA_001015335.1 | N | Y |
| *Tetranychus urticae* | N/A | GCA_000239435.1 | N | Y |

*RNA extraction*

The following protocol was used to extract RNA from *C. sonorensis*:

1. Samples were placed in 100 µl of Schneider’s Drosophila media (Gibco™, Thermofisher Scientific, UK) and homogenised twice for 30 seconds at 25 Hz/s with 3 mm stainless-steel beads.
2. Add 300 µl of Trizol to each sample and incubate at room temperature for 5 minutes.
3. Add 80 µl of Chloroform, mix well by inverting the tubes 10-15 times and incubate at room temperature for 2-3 minutes.
4. Centrifuge samples at maximum speed (18000 x *g*) at 4 ^o^C.
5. Transfer the clear, upper layer to a new tube.
6. Add 100 µl of 100% Isopropanol. Mix well and incubate for 10 minutes at room temperature.
7. Centrifuge samples for 10 minutes at maximum speed at 4 ^o^C. Discard supernatant.
8. Add 500 µl of 75% ethanol. Centrifuge for 5 minutes at maximum speed and discard supernatant.
9. Dry pellet for 5-10 minutes and resuspend in 30 µl of nuclease free water, and incubate the sample at 55 ^o^C for 10 minutes.

*RT-qPCR for the detection of BTV-1 infection*

We used the ProtoScript II First Strand cDNA Synthesis kit (New England Biolabs) to reverse transcribe the RNA into DNA following the manufacturer protocol:

1. Prepare a reaction including: 5 µl of RNA sample, 2 µl of Oligo d(T)_18_ primer and 1 µl of nuclease free water (to a final reaction volume of 8 µl).
2. Incubate the reaction at 65 ^o^C for 5 mminutes.
3. Add 10 µl of ProtoScript II 2X reaction mix and 2 µl of 10X ProtoScript enzyme mix to the reaction (final volume of 20 µl).
4. Incubate 1 hour at 42 ^o^C and five minutes at 80 ^o^C.

The resulting cDNA was then used as template in a real-time qPCR that used SYBR Green assays. The reactions were prepared as following:

| cDNA | 5 µl |
| --- | --- |
| Master Mix with SYBR Green dye (PrimerDesign) | 10 µl |
| Primer BTVuni 291-311F 5’ GCTTTTGAGGTGTACGTGAAC 3’ | 0.5 µl (20 pmol) |
| BTVuni 381-357R 5’ TCTCCCTTGAAACTCTATAATTACG 3’ | 0.5 µl (20 pmol) |
| Nuclease free water | 4 µl |

Incubate the reactions 10 minutes at 95 ^o^C, followed by 50 cycles of 15 seconds at 95 ^o^C, 60 s at 60 ^o^C.

*RT-qPCR protocol for the validation of differentially expressed genes*

RT-qPCR was used to verify the change in expression of four genes of interest, *Toll-like protein*, *glutathione-s-transferase*, *glutathione-s-transferase 1* and *ski2 antiviral helicase*, identified as differentially expressed in the transcriptome analyses. The reactions were performed using the same two biological replicates of the vector competent and refractory *C. sonorensis* transcriptomes that were sequenced.

Specific primers for these genes were designed using Primer3Plus, ensuring that they met the following conditions when possible:

1. Amplicon of 80-120 bp.
2. A Tm of approximately 60 ^o^C.
3. GC content of approximately 40%
4. No 3’ GC clamp.
5. No hairpin loops and primer dimers.

The sequences of the primers and probes designed are:

| Symbol | Gene Name | Accession No. | Primer | | Amplicon Size (bp) | Amplification Efficiency % (R^2^) |
| --- | --- | --- | --- | --- | --- | --- |
|  |  |  | ID | Sequence (‘5-3’) |  |  |
| *TP* | *Toll Protein* | XLOC_000522 | F: | GGCTGACATTGAGGGTGATTTC | 99 | 94.5 (0.99) |
|  |  |  | R: | CGTCCCAAATGAACATGCTTG |  |  |
|  |  |  | P: | TGAACCCTCGCATTGGAAAACCTCTCG |  |  |
| *GST* | *Glutathione-s-Transferase* | XLOC_011745 | F: | CTCAACACAGCGTTCCAAC | 81 | 92.7 (0.98) |
|  |  |  | R: | TGAGGCTCCATTTGGAACTG |  |  |
|  |  |  | P: | CTGACCGAGTCCAAGGCCATTTTGGGA |  |  |
| *GST-1* | *Glutathione s Transferase-1* | XLOC_012462 | F: | TGCCGATTACTGGTATCCTCAA | 127 | 93.8 (0.98) |
|  |  |  | R: | CCCATTGCTTCTTCCATTTTCT |  |  |
|  |  |  | P: | TTTGGCAAACAACCCGCAAATCCA |  |  |
| *SKI-2* | *Antiviral Helicase* | XLOC_006435 | F: | TGAGACCACCCAAAATGCAG | 101 | 96.8 (0.99) |
|  |  |  | R: | AAATGACCCCATCGCAATCG |  |  |
|  |  |  | P: | TGCGTGGTCCTGAAACAGTCCACAACA |  |  |

RT-qPCR reactions were carried out using the Superscript® III One-Step RT-qPCR System with Platinum^®^ Taq DNA polymerase (ThermoFisher Scientific, UK). Reactions were performed in a total volume of 25 µl consisting of:

| Nuclease free water | 5.5 µl |
| --- | --- |
| 2x reaction mix | 12.5 µl |
| SuperScript III/PlatinumTaq Mix | 0.5 µl |
| ROX reference dye (1:10 dilution) | 0.5 µl |
| 10 µM Primer forward | 0.5 µl |
| 10 µM Primer reverse | 0.5 µl |
| Hydrolysis probe | 1 µl |
| RNA template | 4 µl |

Reaction cycles consisted of:

| 1 cycle | cDNA synthesis | 50 ^o^C | 5 minutes |
| --- | --- | --- | --- |
| 1 cycle | Denaturation step | 94 ^o^C | 2 minutes |
| 40 cycles | Denaturation | 94 ^o^C | 3 seconds |
|  | Amplification | 56 ^o^C | 30 seconds |

*Alignment of gst-1 and ski2*

RNA sequences were translated into the corresponding amino acid sequences using BioEdit. These protein sequences were then aligned using the command version of T-Coffee. The commands used were:

# Command to align the sequences

t_coffee -seq protein_sequences.fas -mode expresso -output fasta_aln

# Command to evaluate the alignment positions

t_coffee -infile protein_alignment.fasta -evaluate -output score_ascii, aln, score_html

# Command to remove less conserved positions with a score <6

t_coffee -other_pg seq_reformat -in protein_alignment.fasta -struc_in protein_aln.score_ascii -struc_in_f number_aln -action +keep '[7-9]' > protein_alignment_conserved.fasta

# Finally, I removed the gap only columns

t_coffee -other_pg seq_reformat -in protein_alignment_conserved.fasta -action +rm_gap 100 > protein_alignment_conserved.fasta
