# Supplementary material for: The genome of the biting midge Culicoides sonorensis and gene expression analyses of vector competence for bluetongue virus
Source: BMC Genomics. 2018 Aug 22;19:624. doi: 10.1186/s12864-018-5014-1 (PMC6106943; doi:10.1186/s12864-018-5014-1)
Supplement: Supplementary file 2 — Table S1. Differentially expressed genes between vector competent and vector refractory C. sonorensis females. Table S2. BLAST results for the differentially expressed genes between vector competent and refractory transcriptomes. Table S3. Ranking of the expression stability of the reference genes used in RT-qPCR. Table S4. Primers and probe used for RT-qPCR of ski2, gst, gst-1 and Toll-like. Table S5. Difference of expression levels in four genes (Toll-like, gst, gst-1 and ski2) between vector-competent and vector-refractory females assessed by RNAseq and RT-qPCR. Table S6. Orthology analysis of two of genes Ski2 (a) and gst-1 (b). Table S7. Immune pathways genes identified in C. sonorensis using the Ensembl Compara pipeline. Table S8. Homologue immune genes associated with Toll (a), Imd (b) and Jak/Stat (c). Table S9. Immune related genes identified in C. sonorensis using blastp. Table S10. Details of the blastp results. Table S11. Read mapping and estimate of assembly size according to the method of Schell et al... [36]. Table S12. BUSCO analysis results. Table S13. Number of distinct InterPro and GO terms annotated C. sonorensis and six other Diptera species. Table S14. Tandemly repeated gene arrays in C. sonorensis versus other insect species. [file 12864_2018_5014_MOESM2_ESM.pdf]

Table S1. Differentially expressed genes between vector competent and vector refractory *C. sonorensis* females identified using CummeRbund. The table shows the fpkm values for each phenotype, the log2 fold change of expression levels and the significance level of the change. Genes that are not annotated in the *C. sonorensis* genome are denoted with the name XLOC_XXXXXX. Genes belonging to an enriched GO term are shown in bold.

Table S2. BLAST results for the differentially expressed genes between vector competent and refractory transcriptomes showing the top hit as determined by BLAST.

Table S3. Ranking of the expression stability of the reference genes tested for use in the quantitative reverse transcription PCR (RT-qPCR). Three different tests were used to rank the expression stability of the genes, BestKeeper, gNorm and NormFinder. *r*- correlation coefficient, Cq – quantification cycle, SE – standard error, SD – standard deviation.

Table S4. Primers forward (F), reverse (R) and hydrolysis probe (P) sequences used for RT-qPCR analysis of the expression levels of *ski2*, *gst*, *gst-1* and *Toll*-like.

Table S5. Difference of expression levels in four genes (*Toll*-like, *gst*, *gst-1* and *ski2*) between vector-competent and vector-refractory females assessed by RNAseq and RT-qPCR. * denote a difference in expression between refractory and competent females with a $P < 0.05$ (RT-qPCR significance estimated using a Welch Two Sample t-test with d.f. = 1).

Table S6. Orthology analysis of two of genes *Ski2* **(a)** and *gst-1* **(b)**.

Table S7. Immune pathways genes identified in *C. sonorensis* using the Ensembl Compara pipeline and the *D. melanogaster* genes as reference. **a)** Toll pathway; **b)** Imd pathway; **c)** Jak/Stat pathway.

Table S8. Homologue immune genes in several invertebrate species identified using *D. melanogaster* genes as reference all with InterPro and GO terms associated with Toll **(a)**, Imd **(b)** and Jak/Stat **(c)**, in the Ensembl Compara pipeline.

Table S9. Immune related genes identified in *C. sonorensis* using blastp top hits with the gene copies from *Ae. aegypti* and *Cx. quinquefasciatus* available in the ImmunoDB database (http://cegg.unige.ch/Insecta/immunodb) as query. a) Anti-Microbial Peptides; b) Toll receptors; c) Toll path; d) jak/Stat path; e). Imd path. * - Top blastp hits different found when *Ae. aegypti* and *Cx. quinquefasciatus* were used as query.

Table S10. Details of the blastp results using *Ae. aegypti* (a) and *Cx. quinquefasciatus* (b) as query to identify the *C. sonorensis* genes of the immune pathways.

Table S11. Read mapping and estimate of assembly size, by read mapping using BWA, according to the method of Schell et al. [36].

Table S12. BUSCO analysis using the Insecta data set (version 3.0.2) against the assembled genome sequence of six species, including *Culicoides sonorensis*.

Table S13. Number of distinct InterPro and GO terms annotated to annotated proteins of *C. sonorensis* and six other Diptera species.

Table S14. Tandemly repeated gene arrays in *C. sonorensis* versus other insect species. Longest gene arrays in *C. sonorensis* with matching InterPro domain architecture and maximum 100,000 nucleotide between successive members. The longest array with the same domain signature from 3 other insect species is given for comparison.

Table S1. Differentially expressed genes between vector competent and vector refractory *C. sonorensis* females identified using CummeRbund. The table shows the fpkm values for each phenotype, the log2 fold change of expression levels and the significance level of the change. Genes that are not annotated in the *C. sonorensis* genome are denoted with the name XLOC_XXXXXX. Genes belonging to an enriched GO term are shown in bold.

Gene Name	Scaffold	Gene Start	Gene Stop	FPKM refractory	FPKM competent	log2 fold change	Test stat	p value	q value
CSON000187	1020	56965	62253	95.7715	246.505	1.36395	2.47943	5.00E-05	0.0007223
CSON000338	105	98591	100543	1.70631	3.93991	1.20729	1.86748	7.00E-04	0.00675514
CSON000317	1052	51749	54289	7.02465	17.7608	1.3382	2.49969	5.00E-05	0.0007223
XLOC_000522	1077	33560	35356	97.7819	226.246	1.21025	2.4628	5.00E-05	0.0007223
CSON000775	1121	59621	60212	73.4031	29.2175	-1.32901	-2.35313	5.00E-05	0.0007223
XLOC_000906	1126	32712	33483	258.593	509.34	0.977945	2.03373	5.00E-05	0.0007223
CSON000815	1131	68219	72505	1.42713	6.82676	2.25809	3.21323	5.00E-05	0.0007223
CSON000864	1140	65981	67669	50.3918	26.179	-0.944779	-1.92118	4.00E-04	0.00427634
CSON000869	1144	17705	21551	447.734	159.538	-1.48874	-2.54122	5.00E-05	0.0007223
XLOC_001126	116	100463	100979	8.12222	3.18883	-1.34885	-1.77189	7.00E-04	0.00675514
CSON001067	116	79874	99233	198.167	68.2954	-1.53686	-1.82685	4.00E-04	0.00427634
XLOC_001160	116	69296	69913	80.9861	22.2166	-1.86604	-3.24669	5.00E-05	0.0007223
CSON001013	1166	15031	19340	0.450688	1.12644	1.32157	2.01935	5.00E-05	0.0007223
CSON001024	1167	18005	20284	12.4169	29.4365	1.2453	2.42018	5.00E-05	0.0007223
CSON001105	1173	61664	66373	1.65707	3.96647	1.25922	1.93816	0.00015	0.00188533
CSON001385	1201	62781	63686	113.959	56.2785	-1.01786	-1.96573	0.00015	0.00188533
CSON001416	1210	46229	46753	226.945	460.41	1.02058	2.03601	5.00E-05	0.0007223
XLOC_001620	1230	3874	4690	165.101	74.4443	-1.14911	-2.14791	5.00E-05	0.0007223
CSON001531	1243	13127	13740	8.12842	37.8511	2.21929	3.3278	5.00E-05	0.0007223
CSON001649	1261	35264	40952	23.7174	63.3795	1.41807	2.20552	1.00E-04	0.00132614
CSON001655	1263	30790	31318	142.483	373.534	1.39045	2.72745	5.00E-05	0.0007223
XLOC_002074	1310	18586	18934	175.487	10.2261	-4.10104	-6.29543	5.00E-05	0.0007223

CSON002003	1324	11971	14487	98.0295	43.0229	-1.18811	-2.13703	5.00E-05	0.0007223
CSON002029	1329	24857	28424	0.211386	1.40134	2.72886	2.77239	5.00E-05	0.0007223
CSON002051	1336	51374	52065	60.1969	148.755	1.30518	2.05441	1.00E-04	0.00132614
CSON002063	1338	65780	68748	43.0846	23.7657	-0.85829	-1.7182	0.00045	0.00470463
XLOC_002281	1345	193	730	189.823	73.1192	-1.37633	-2.57974	5.00E-05	0.0007223
CSON002102	1349	43394	44629	77.796	41.1419	-0.919086	-1.74282	0.00035	0.00381891
XLOC_002494	1384	42903	43297	1.2211	5.88208	2.26815	2.35326	0.00045	0.00470463
CSON002476	1411	22374	24874	53.7599	25.5064	-1.07567	-1.78101	7.00E-04	0.00675514
CSON002750	1464	6279	7832	18.1208	8.51545	-1.08949	-1.87778	0.00045	0.00470463
XLOC_002993	1468	3009	3851	12.2336	4.63622	-1.39983	-2.30499	5.00E-05	0.0007223
CSON002905	1498	8640	13502	99.8978	202.059	1.01625	1.89819	1.00E-04	0.00132614
XLOC_003130	1498	87846	89699	3.78694	1.09018	-1.79647	-2.1578	5.00E-04	0.00512541
CSON003327	15	401972	405247	5.2211	12.4366	1.25216	2.15266	5.00E-05	0.0007223
CSON003671	16	23199	25412	3.16834	1.20189	-1.39842	-1.96605	5.00E-05	0.0007223
XLOC_003777	1639	28994	29752	0.976353	4.15013	2.08768	2.36307	5.00E-05	0.0007223
XLOC_003963	17	18054	19231	0.473089	1.93203	2.02993	2.1683	0.00015	0.00188533
CSON003756	1716	7125	7847	265.795	100.728	-1.39985	-2.70392	5.00E-05	0.0007223
CSON003799	1726	1904	3437	18.4123	6.92785	-1.41019	-2.51201	5.00E-05	0.0007223
CSON004017	176	138362	139806	113.586	62.1894	-0.869047	-1.75964	3.00E-04	0.00336372
CSON003990	1764	45413	56759	304.009	81.3857	-1.90127	-2.36281	5.00E-05	0.0007223
CSON004009	1769	43108	52782	55.9668	115.43	1.04437	2.13678	5.00E-05	0.0007223
CSON004091	1781	40392	49726	4.73052	9.00849	0.929288	1.68473	0.00035	0.00381891
CSON004099	1784	18998	21333	257.39	126.598	-1.02371	-1.86272	5.00E-04	0.00512541
XLOC_004410	1793	4447	5105	129.462	68.3006	-0.92256	-1.82888	4.00E-04	0.00427634
XLOC_004465	1809	37312	37974	3.06949	10.3872	1.75874	2.37093	5.00E-05	0.0007223
XLOC_004498	1813	19613	20169	9.98673	25.7683	1.36751	2.24691	5.00E-05	0.0007223
XLOC_004499	1814	21775	22656	0.588509	6.00433	3.35087	3.9488	5.00E-05	0.0007223

CSON004363	185	33155	33934	4.50091	0.595649	-2.91768	-2.87605	5.00E-05	0.0007223
CSON004589	192	197900	200721	70.401	36.273	-0.956699	-1.86906	5.00E-05	0.0007223
CSON004693	195	140963	142582	4.61331	11.0792	1.26398	2.05572	1.00E-04	0.00132614
CSON004795	197	185015	186393	36.5385	15.8668	-1.2034	-2.16754	5.00E-05	0.0007223
CSON004801	197	199150	200552	57.5093	29.1181	-0.981881	-1.87357	3.00E-04	0.00336372
CSON004881	1998	34965	35839	16.419	6.14239	-1.41849	-1.97817	2.00E-04	0.00239421
CSON004935	200	206400	206867	56.2492	29.5679	-0.927803	-1.67033	0.00055	0.0055551
XLOC_005422	2031	17882	18879	2.00328	5.67126	1.5013	2.24469	1.00E-04	0.00132614
CSON005106	2051	3628	6110	1.23595	3.12648	1.33892	2.04306	5.00E-05	0.0007223
CSON005127	2059	4528	6474	19.1704	5.74318	-1.73896	-2.81326	5.00E-05	0.0007223
CSON005199	2087	27350	31880	155.944	45.2282	-1.78573	-3.15147	5.00E-05	0.0007223
CSON005197	2087	10702	14012	297.427	105.087	-1.50095	-2.69665	5.00E-05	0.0007223
XLOC_005617	2087	20899	22245	110.156	35.9376	-1.61598	-3.07156	5.00E-05	0.0007223
XLOC_005619	2087	23007	23346	8.54346	2.13286	-2.00203	-2.16266	2.00E-04	0.00239421
CSON005326	2121	27568	29902	7.75424	15.5477	1.00364	1.77582	0.00045	0.00470463
CSON005608	219	100110	101543	23.2423	7.62527	-1.60789	-3.10356	5.00E-05	0.0007223
XLOC_006034	219	87547	89510	6.65684	15.7725	1.2445	2.12597	1.00E-04	0.00132614
CSON005662	2203	6744	8009	31.7812	14.335	-1.14863	-2.0339	1.00E-04	0.00132614
CSON005924	2283	19588	23330	2.07841	4.80776	1.20989	1.95428	5.00E-05	0.0007223
XLOC_006435	2288	11614	20912	11.227	5.22611	-1.10316	-1.91407	0.00035	0.00381891
CSON005962	2291	91	3498	10.091	32.0699	1.66815	3.12074	5.00E-05	0.0007223
CSON006137	2335	26291	27842	27.7014	70.5047	1.34776	2.26021	5.00E-05	0.0007223
CSON006239	235	114393	116471	0.405211	1.20099	1.56748	1.95044	0.00015	0.00188533
CSON006343	2397	4787	12689	12.8251	25.6412	0.999492	1.90126	0.00035	0.00381891
CSON006427	2418	9366	10870	34.7099	17.8025	-0.96327	-1.82833	0.00025	0.00288827
CSON006459	2436	23578	24927	9.82289	4.60844	-1.09187	-1.773	0.00045	0.00470463
CSON006540	244	96230	98507	29.7092	14.9239	-0.993288	-1.81837	0.00015	0.00188533

CSON006666	2503	4064	4919	40.2831	20.2699	-0.990838	-1.79855	0.00035	0.00381891
XLOC_007274	2545	21613	24429	2.89377	8.48676	1.55226	2.66561	5.00E-05	0.0007223
XLOC_007339	2574	21587	22109	13.8662	1.98576	-2.80381	-3.61956	5.00E-05	0.0007223
CSON007080	2660	1	1971	81.5527	39.227	-1.05588	-1.95475	0.00025	0.00288827
CSON007149	2692	18525	22579	14.151	7.09892	-0.99523	-1.83292	3.00E-04	0.00336372
XLOC_008396	2934	3826	4844	23.6625	11.4867	-1.04265	-1.82006	3.00E-04	0.00336372
CSON007839	2956	17408	17986	23.4494	6.34698	-1.88541	-2.86823	5.00E-05	0.0007223
CSON007842	2959	12246	12917	0	0.923198	Inf	NA	5.00E-05	0.0007223
CSON007985	300	69361	70256	234.896	94.4757	-1.31401	-2.50657	5.00E-05	0.0007223
CSON008025	3023	3869	9063	0.373474	4.25367	3.50963	3.97632	5.00E-05	0.0007223
CSON008079	304	70719	72259	13.3453	27.2844	1.03175	2.08503	5.00E-05	0.0007223
CSON008127	3065	10647	16367	2.28047	4.96718	1.12309	1.82794	3.00E-04	0.00336372
CSON008129	3067	18228	18866	15.0304	5.78178	-1.3783	-2.08217	1.00E-04	0.00132614
CSON008188	3099	1251	10628	0.429014	1.52743	1.83201	1.82384	6.00E-04	0.005979
XLOC_008922	3112	13857	14666	3.66233	0.981	-1.90044	-2.01081	6.00E-04	0.005979
CSON008795	3374	3752	4383	9.07578	2.86251	-1.66474	-2.32731	5.00E-05	0.0007223
XLOC_009596	3424	1	1971	40.7008	10.7574	-1.91972	-2.46404	5.00E-05	0.0007223
CSON009017	349	49192	51495	1.2807	0.244776	-2.3874	-2.76451	5.00E-05	0.0007223
CSON009096	3548	2772	3638	17.5404	7.87752	-1.15487	-2.04969	5.00E-05	0.0007223
CSON009103	3558	6091	7057	89.6882	45.4448	-0.980805	-1.9482	0.00015	0.00188533
CSON009221	362	46351	47006	9.76258	3.71458	-1.39406	-1.92677	0.00015	0.00188533
XLOC_009940	3628	859	1599	78.7691	27.9312	-1.49575	-2.96971	5.00E-05	0.0007223
CSON009366	3710	2907	6631	0.982249	2.24479	1.19242	1.9481	2.00E-04	0.00239421
XLOC_010162	3732	4880	5807	182.281	54.7696	-1.73472	-3.16375	5.00E-05	0.0007223
CSON009491	377	1	1971	6.82438	17.5471	1.36246	2.39917	5.00E-05	0.0007223
XLOC_010389	3826	1814	2282	244.61	89.5316	-1.45001	-2.72499	5.00E-05	0.0007223
CSON009775	391	157877	159235	1.79163	5.20891	1.53971	2.06361	1.00E-04	0.00132614

CSON009861	3959	5414	8173	98.8141	221.341	1.16349	2.29728	5.00E-05	0.0007223
CSON009907	3982	4099	6158	54.2875	23.6148	-1.20093	-2.2384	5.00E-05	0.0007223
CSON010114	40	233585	234257	81.6124	33.0626	-1.30359	-2.00081	5.00E-05	0.0007223
CSON010472	4263	4995	6247	7.8979	3.61012	-1.12942	-1.79226	0.00035	0.00381891
CSON010672	43	11481	14179	1.96423	4.77369	1.28114	2.03159	5.00E-05	0.0007223
CSON010561	4329	1	1971	34.261	18.508	-0.888419	-1.72483	4.00E-04	0.00427634
CSON010570	433	1156	7392	91.6531	170.03	0.891535	1.70449	7.00E-04	0.00675514
CSON010582	4342	5379	6064	54.294	26.7436	-1.0216	-1.83417	0.00025	0.00288827
CSON010621	4373	205	861	21.8658	8.93811	-1.29064	-1.98284	2.00E-04	0.00239421
CSON010831	449	62665	65789	23.8139	10.3189	-1.20651	-2.16333	5.00E-05	0.0007223
CSON010973	45	405891	410616	0.653425	2.75559	2.07627	2.81474	5.00E-05	0.0007223
XLOC_011853	459	129049	129769	37.2031	16.284	-1.19197	-2.10935	0.00015	0.00188533
CSON011113	4708	1113	3138	3.23942	6.43428	0.990046	1.6794	6.00E-04	0.005979
CSON011327	490	33519	34587	18.234	9.23575	-0.981328	-1.82721	0.00035	0.00381891
CSON011322	4907	1	1971	45.7696	19.9332	-1.19921	-2.05637	5.00E-05	0.0007223
XLOC_012379	5	544225	544994	20.716	41.649	1.00754	1.82364	0.00035	0.00381891
CSON011559	504	97958	104687	1648.64	787.321	-1.06625	-1.78998	0.00055	0.0055551
XLOC_012476	5049	1	1971	12.9362	30.5799	1.24117	2.53637	5.00E-05	0.0007223
CSON011720	521	9003	15835	3.12411	0.744022	-2.07003	-2.16228	0.00035	0.00381891
CSON011951	5448	530	2002	4.84366	12.9238	1.41586	2.67524	5.00E-05	0.0007223
CSON011956	545	118392	123690	1.60271	3.76688	1.23286	2.04662	5.00E-05	0.0007223
CSON012191	5601	533	1558	1.62093	4.38093	1.43442	1.97125	4.00E-04	0.00427634
XLOC_013324	577	114028	114538	6.02354	1.68568	-1.83729	-2.21531	5.00E-05	0.0007223
XLOC_013345	579	58356	59843	1.36252	3.23309	1.24664	1.7859	0.00065	0.00637417
CSON012392	581	58	807	80.6613	43.4763	-0.891649	-1.72165	0.00065	0.00637417
XLOC_013421	589	63243	63757	9.19563	29.1125	1.66262	2.39617	5.00E-05	0.0007223
XLOC_013523	598	252	798	139.028	60.0964	-1.21002	-2.38446	5.00E-05	0.0007223

CSON012597	602	29312	30535	29.1673	59.0625	1.01789	1.96588	5.00E-05	0.0007223
XLOC_013832	618	1924	2497	39.3724	20.572	-0.936501	-1.7414	7.00E-04	0.00675514
XLOC_013834	618	4353	4925	23.8689	6.42005	-1.89447	-3.10882	5.00E-05	0.0007223
CSON012843	62	101498	102803	3.82904	9.69778	1.34067	2.14509	1.00E-04	0.00132614
CSON012899	631	8632	9938	16.2488	34.7763	1.09777	1.94163	1.00E-04	0.00132614
CSON012905	6339	1916	2533	20.3128	9.0154	-1.17193	-1.8997	2.00E-04	0.00239421
CSON012932	634	57069	60555	274.48	143.211	-0.93856	-1.7408	6.00E-04	0.005979
CSON012968	637	53357	54442	42.5389	91.8825	1.11101	2.19093	5.00E-05	0.0007223
CSON013373	673	204294	206282	13.2467	6.05273	-1.12998	-2.04886	5.00E-05	0.0007223
CSON013428	676	39469	46437	22.4464	11.2553	-0.995877	-1.829	2.00E-04	0.00239421
XLOC_014619	6808	651	836	67.777	24.1952	-1.48607	-1.96861	2.00E-04	0.00239421
CSON013527	682	36237	39706	0.696962	2.28902	1.71558	1.94929	0.00025	0.00288827
CSON014437	7	499145	500351	437.617	226.191	-0.952129	-1.90444	2.00E-04	0.00239421
CSON013840	7218	5	686	11.9905	25.96	1.1144	1.92927	1.00E-04	0.00132614
CSON013982	746	14422	16497	127.153	65.4298	-0.958543	-1.88756	3.00E-04	0.00336372
CSON014001	749	118435	119765	27.0904	13.0923	-1.04906	-1.98064	5.00E-05	0.0007223
XLOC_015460	787	60873	63062	0.996112	2.40919	1.27417	1.70969	7.00E-04	0.00675514
CSON014343	793	17744	18476	438.048	132.611	-1.72389	-3.21218	5.00E-05	0.0007223
CSON014342	793	15338	16691	1123.47	415.153	-1.43625	-2.58394	5.00E-05	0.0007223
XLOC_015579	802	14	1749	0.79423	2.48667	1.64659	2.10301	0.00035	0.00381891
CSON014544	82	12575	17266	22.291	8.3824	-1.41103	-2.488	5.00E-05	0.0007223
CSON014796	86	43596	45426	41.9855	15.8691	-1.40367	-2.13754	5.00E-05	0.0007223
CSON014785	86	236765	237789	753.039	286.757	-1.39289	-2.52016	5.00E-05	0.0007223
XLOC_015917	86	50857	51803	9.07849	19.9167	1.13346	1.9909	0.00015	0.00188533
CSON014747	866	44446	44957	170.432	89.4205	-0.930516	-1.78972	0.00065	0.00637417
CSON014863	880	76150	78714	35.6126	19.0485	-0.902713	-1.74631	3.00E-04	0.00336372
XLOC_016036	880	66840	69032	0.225636	1.24541	2.46455	2.72009	5.00E-05	0.0007223

CSON015088	929	68	34564	2.3335	5.35455	1.19827	2.02686	1.00E-04	0.00132614
CSON015183	93	294174	295795	83.5612	43.0265	-0.957607	-1.92989	0.00015	0.00188533
XLOC_016385	939	28792	29210	7.46809	2.58586	-1.5301	-1.86189	2.00E-04	0.00239421
CSON015393	978	20183	21423	3.3351	9.35691	1.4883	2.33107	5.00E-05	0.0007223
CSON015491	98	125029	125784	326.428	174.555	-0.903083	-1.74333	0.00035	0.00381891
CSON015470	986	56873	60387	1.04051	2.42003	1.21773	1.86553	0.00025	0.00288827
CSON015614	99	36162	36654	5.89291	15.5546	1.40029	1.81653	5.00E-04	0.00512541
XLOC_016778	993	18437	19620	16.1313	32.4132	1.00672	1.89318	0.00015	0.00188533

Table S2. BLAST results for the differentially expressed genes between vector competent and refractory transcriptomes showing the top hit as determined by BLAST.

Gene Name	Sequence description	Hit ACC	Species	E-Value	Similarity (%)
CSON000187	AAEL006095-PA	XP_001657431	*Aedes aegypti*	0	74
CSON000338	golgi-associated plant pathogenesis-related protein 1	KDR20657	*Zootermopsis nevadensis*	1.88E-99	63
CSON000317	No hit	No hit			
XLOC_000522	protein toll	XP_001948700	*Acyrthosiphon pisum*	9.86E-16	49
CSON000775	u6 snrna-associated sm-like protein lsm7	XP_969723	*Tribolium castaneum*	5.68E-58	88
XLOC_000906	unknown salivary protein	AAU06489	*Culicoides sonorensis*	9.16E-14	100
CSON000815	hypothetical protein FF38_05739	KNC28514	*Lucilia cuprina*	7.05E-15	48
CSON000864	angiotensin-converting enzyme	KFB40455	*Anopheles sinensis*	0	85
CSON000869	histidine protein methyltransferase 1 homolog isoform x1	XP_013164242	*Papilio xuthus*	5.35E-58	59
XLOC_001126	No hit	No hit			
CSON001067	histone h2b-like	AAEL015674	*Aedes aegypti*	7.43E-57	100
XLOC_001160	histone type 2	XP_001865500	*Culex quinquefasciatus*	4.75E-81	100
CSON001013	dep domain-containing protein 5 isoform x7	XP_011291541	*Musca domestica*	0	62
CSON001024	Tenascin - partial	XP_012251779	*Athalia rosae*	2.45E-32	44
CSON001105	Hypothetical protein	ETN58203	*Anopheles darlingi*	7.50E-150	64
CSON001385	No hit	No hit			
CSON001416	No hit	No hit			
XLOC_001620	histone h2a	ABV60391	*Artemia franciscana*	1.25E-51	75
CSON001531	No hit	No hit			
CSON001649	filaggrin-2 isoform x3	XP_001655555	*Aedes aegypti*	6.70E-18	58
CSON001655	No hit	No hit			
XLOC_002074	No hit	No hit			
CSON002003	proteasome subunit alpha type 6	XP_001654091	*Aedes aegypti*	6.88E-112	93
CSON002029	vesicular mannose-binding lectin	EHJ65368	*Danaus plexippus*	3.75E-16	47

CSON002051	achain b-glucan binding domain of drosophila gnbp3	3IE4_A	*Drosophila melanogaster*	8.51E-39	78
CSON002063	barrier-to-autointegration factor isoform x7	XP_314872	*Anopheles gambiae*	3.82E-50	97
XLOC_002281	histone h2a	XP_002045914	*Drosophila sechellia*	4.50E-68	100
CSON002102	single-stranded dna-binding mitochondrial	XP_013099105	*Stomoxys calcitrans*	3.04E-63	89
XLOC_002494	No hit	No hit			
CSON002476	prenylated rab acceptor protein 1	XP_562528	*Anopheles gambiae*	9.60E-58	82
CSON002750	dna polymerase epsilon subunit 4	XP_011206610	*Bactrocera dorsalis*	7.33E-30	70
XLOC_002993	No hit	No hit			
CSON002905	AAEL006095-PA	XP_001657431	*Aedes aegypti*	0	74
XLOC_003130	agap002182-pa-like protein	XP_001660605	*Aedes aegypti*	5.69E-11	67
CSON003327	agap011604-pa-like protein	XP_001867876	*Culex quinquefasciatus*	1.83E-27	71
CSON003671	tigger transposable element-derived protein 6-like partial	KMQ88832	*Lasius niger*	2.00E-73	56
XLOC_003777	No hit	No hit			
XLOC_003963	No hit	No hit			
CSON003756	histidine triad nucleotide-binding protein 1	XP_013098805	*Stomoxys calcitrans*	2.10E-71	75
CSON003799	lymphokine-activated killer t-cell-originated protein kinase	XP_011180005	*Bactrocera cucurbitae*	4.94E-99	68
CSON004017	lrr47 protein	XP_001863468	*Culex quinquefasciatus*	2.95E-96	62
CSON003990	No hit	No hit			
CSON004009	apolipoprotein d	XP_001663762	*Aedes aegypti*	8.65E-102	77
CSON004091	pickpocket protein 28	XP_001844855	*Culex quinquefasciatus*	0	70
CSON004099	isocitrate dehydrogenase	XP_001845030	*Culex quinquefasciatus*	0	84
XLOC_004410	secreted salivary protein	AAU06546	*Culicoides sonorensis*	1.97E-112	100
XLOC_004465	No hit	No hit			
XLOC_004498	No hit	No hit			
XLOC_004499	No hit	No hit			
CSON004363	No hit	No hit			
CSON004589	pre-mrna-splicing factor cwc25 homolog	XP_001848085	*Culex quinquefasciatus*	7.01E-64	72

CSON004693	polyphosphate kinase 2 family	XP_001866786	*Culex quinquefasciatus*	6.44E-15	61
CSON004795	structure-specific endonuclease subunit slx1	XP_012253571	*Athalia rosae*	3.91E-71	65
CSON004801	fk506-binding protein 2	ETN61115	*Anopheles darlingi*	5.64E-62	93
CSON004881	AAEL001403-PA	XP_001659187	*Aedes aegypti*	3.83E-52	91
CSON004935	signal recognition particle protein 19	ETN62677	*Anopheles darlingi*	9.31E-58	75
XLOC_005422	leucine-rich repeat neuronal protein partial	KFM78261	*Stegodyphus mimosarum*	2.75E-13	58
CSON005106	AAEL007906-PA	XP_001652996	*Aedes aegypti*	4.19E-61	69
CSON005127	n-acetylneuraminate lyase-like	XP_011259060	*Camponotus floridanus*	2.40E-47	65
CSON005199	5 -nucleotidase domain-containing protein 3 isoform x1	XP_012138434	*Megachile rotundata*	0	84
CSON005197	branched-chain-amino-acid cytosolic-like	KNC23357	*Lucilia cuprina*	2.15E-139	72
XLOC_005617	histone type 2	XP_001865500	*Culex quinquefasciatus*	2.27E-89	100
XLOC_005619	histone h2a	XP_001600014	*Nasonia vitripennis*	5.51E-32	100
CSON005326	No hit	No hit			
CSON005608	agap002151-pa-like protein / protein rcc2 homolog	XP_001653306	*Aedes aegypti*	0	83
XLOC_006034	No hit	No hit			
CSON005662	aldo-keto reductase	XP_001648454	*Aedes aegypti*	9.57E-159	83
CSON005924	isoform a	XP_001650083	*Aedes aegypti*	4.10E-70	54
XLOC_006435	antiviral helicase ski2	XP_001845019	*Culex quinquefasciatus*	0	73
CSON005962	cell wall cysteine-rich protein	XP_001688376	*Anopheles gambiae*	9.75E-29	35
CSON006137	unknown	AAV84224	*Culicoides sonorensis*	2.41E-11	51
CSON006239	glucosylceramidase	XP_001846785	*Culex quinquefasciatus*	0	69
CSON006343	GK20873	XP_002061352	*Drosophila willistoni*	6.05E-132	60
CSON006427	origin recognition complex subunit 4	XP_001846465	*Culex quinquefasciatus*	1.39E-163	71
CSON006459	AAEL001172-PA	XP_001658185	*Aedes aegypti*	1.09E-74	62
CSON006540	agap012477-pa-like protein	XP_001653834	*Anopheles gambiae*	5.06E-160	71
CSON006666	small nuclear ribonucleoprotein sm d3	XP_001847040	*Culex quinquefasciatus*	8.86E-63	98
XLOC_007274	gamma-glutamyltranspeptidase 1-like isoform x2	KNC28669	*Lucilia cuprina*	1.30E-23	40

XLOC_007339	No hit	No hit			
CSON007080	gilt-like protein	XP_975539	*Tribolium castaneum*	3.95E-45	62
CSON007149	AAEL012981-PA	XP_001663173	*Aedes aegypti*	4.31E-104	58
XLOC_008396	No hit	No hit			
CSON007839	dna polymerase epsilon subunit 3	KFB48038	*Anopheles sinensis*	4.71E-38	82
CSON007842	chromobox protein homolog 1-like isoform x2	EHJ79111	*Danaus plexippus*	5.85E-68	77
CSON007985	No hit	No hit			
CSON008025	vesicular integral-membrane protein vip36	XP_002069798	*Drosophila willistoni*	1.22E-13	46
CSON008079	deltamethrin resistance-associated nyd-op7	AAU06481	*Culicoides sonorensis*	0	100
CSON008127	tubulin beta chain	XP_001844634	*Culex quinquefasciatus*	0	98
CSON008129	scavenger receptor cysteine-rich protein	XP_967476	*Tribolium castaneum*	5.11E-22	53
CSON008188	No hit	No hit			
XLOC_008922	No hit	No hit			
CSON008795	probable dna replication complex gins protein psf2	XP_005191077	*Musca domestica*	4.49E-72	77
XLOC_009596	histone -like	XP_003746459	*Metaseiulus occidentalis*	3.03E-11	86
CSON009017	serine threonine-protein kinase rio2	XP_001851134	*Culex quinquefasciatus*	2.87E-61	66
CSON009096	No hit	No hit			
CSON009103	No hit	No hit			
CSON009221	peptidoglycan recognition protein 2 short class	KFB50649	*Anopheles sinensis*	1.63E-85	79
XLOC_009940	No hit	No hit			
CSON009366	collagen alpha-2 chain	XP_001846673	*Culex quinquefasciatus*	0	67
XLOC_010162	attacin-like partial	XP_013147919	*Papilio polytes*	1.18E-23	63
CSON009491	outer dense fiber protein 3	XP_001648052	*Aedes aegypti*	1.51E-47	76
XLOC_010389	No hit	No hit			
CSON009775	chitin deacetylase 9 precursor	NP_001103904	*Tribolium castaneum*	7.04E-132	63
CSON009861	agap001826-pa-like protein	XP_001849310	*Culex quinquefasciatus*	8.40E-174	62
CSON009907	malate dehydrogenase	ETN62258	*Anopheles darlingi*	3.25E-163	88

CSON010114	transcriptional regulator cuda	XP_002003207	*Drosophila mojavensis*	2.94E-13	62
CSON010472	protein o-fucosyltransferase 1	XP_001651293	*Aedes aegypti*	0	85
CSON010672	agap002583-pa-like protein	XP_001648701	*Aedes aegypti*	6.30E-69	61
CSON010561	ruvb-like helicase 1	XP_001649604	*Aedes aegypti*	0	94
CSON010570	uridine phosphorylase	XP_001651486	*Aedes aegypti*	0	89
CSON010582	conserved membrane protein	ABF18205	*Aedes aegypti*	4.74E-97	89
CSON010621	cdgsh iron-sulfur domain-containing protein mitochondrial	XP_001648564	*Aedes aegypti*	1.49E-47	70
CSON010831	agap008489-pa-like protein	XP_001654982	*Aedes aegypti*	4.99E-132	71
CSON010973	glutathione-s-transferase gst	XP_001654620	*Aedes aegypti*	1.43E-55	63
XLOC_011853	No hit	No hit			
CSON011113	aromatic amino acid decarboxylase	KFB39133	*Anopheles sinensis*	0	81
CSON011327	protein lin-52 homolog	XP_001845066	*Aedes aegypti*	5.37E-34	70
CSON011322	d-tyrosyl-trna deacylase 1	XP_011199324	*Bactrocera dorsalis*	7.69E-62	83
XLOC_012379	No hit	No hit			
CSON011559	glutathione s transferase-1	AAB94639	*Culicoides sonorensis*	3.42E-149	96
XLOC_012476	reverse transcriptase	XP_008197387	*Tribolium castaneum*	0	54
CSON011720	AAEL001676-PA	XP_001659971	*Aedes aegypti*	1.93E-46	84
CSON011951	reverse transcriptase	ACJ71597	*Rhynchosciara americana*	1.03E-71	56
CSON011956	polyprotein	CTR11689	*Calliphora vicina*	1.93E-95	54
CSON012191	agap001099-pa-like protein	XP_001658884	*Aedes aegypti*	7.44E-31	79
XLOC_013324	No hit	No hit			
XLOC_013345	serine protease	XP_011293194	*Musca domestica*	4.21E-49	49
CSON012392	exosome complex exonuclease rrp41	KFB49939	*Anopheles sinensis*	1.06E-143	90
XLOC_013421	No hit	No hit			
XLOC_013523	No hit	No hit			
CSON012597	membrane-associated lps-inducible tnf alpha factor protein	ABV44730	*Phlebotomus papatasi*	1.95E-27	75
XLOC_013832	No hit	No hit			

XLOC_013834	No hit	No hit			
CSON012843	anopheles gambiae pest agap012648-pa	XP_001869099	*Culex quinquefasciatus*	1.41E-103	70
CSON012899	No hit	No hit			
CSON012905	proteasome subunit beta type 3	ABF18140	*Aedes aegypti*	6.77E-128	91
CSON012932	serine protease	KFB43072	*Anopheles sinensis*	6.85E-54	65
CSON012968	No hit	No hit			
CSON013373	nedd8-activating enzyme e1 regulatory subunit	XP_001866881	*Culex quinquefasciatus*	0	85
CSON013428	angiotensin-converting enzyme-like	KFB42468	*Anopheles sinensis*	1.59E-131	60
XLOC_014619	No hit	No hit			
CSON013527	No hit	No hit			
CSON014437	coiled-coil domain-containing protein 58	KNC34973	*Lucilia cuprina*	7.50E-51	76
CSON013840	midline fasciclin	XP_001847648	*Culex quinquefasciatus*	3.92E-44	63
CSON013982	splicing arginine serine-rich 2	EFN61874	*Camponotus floridanus*	1.78E-57	96
CSON014001	serine protease mitochondrial	XP_001651966	*Aedes aegypti*	4.38E-173	84
XLOC_015460	No hit	No hit			
CSON014343	heat shock protein 27-like	AEJ88463	*Bactrocera dorsalis*	1.83E-24	53
CSON014342	heat shock protein 27-like	XP_013117383	*Stomoxys calcitrans*	6.09E-38	71
XLOC_015579	No hit	No hit			
CSON014544	scavenger receptor cysteine-rich protein	XP_001866937	*Culex quinquefasciatus*	1.33E-15	47
CSON014796	dna-directed rna polymerase i subunit d	XP_001842649	*Culex quinquefasciatus*	3.76E-22	76
CSON014785	proliferating cell nuclear antigen	XP_001662644	*Aedes aegypti*	5.69E-175	95
XLOC_015917	No hit	No hit			
CSON014747	snrnp sm protein	XP_001842863	*Culex quinquefasciatus*	3.80E-32	96
CSON014863	sulfide quinone reductase	XP_312834	*Anopheles gambiae*	0	80
XLOC_016036	leucine-rich repeat-containing protein 70-like	AAV84224	*Culicoides sonorensis*	2.38E-21	51
CSON015088	tubulin beta-3 chain	XP_314483	*Anopheles gambiae*	0	98
CSON015183	agap009512-pa-like protein	XP_001652834	*Anopheles gambiae*	3.68E-124	61

XLOC_016385	No hit	No hit			
CSON015393	GL10147	XP_002025910	*Drosophila persimilis*	1.11E-58	52
CSON015491	agap001973-pa-like protein	KFB37456	*Anopheles sinensis*	1.91E-135	88
CSON015470	No hit	No hit			
CSON015614	No hit	No hit			
XLOC_016778	No hit	No hit			

Table S3. Ranking of the expression stability of the reference genes tested for use in the quantitative reverse transcription PCR (RT-qPCR). Three different tests were used to rank the expression stability of the genes, BestKeeper, gNorm and NormFinder. *r*- correlation coefficient, Cq – quantification cycle, SE – standard error, SD – standard deviation.

Reference Gene	Cq (SE)	BestKeeper		geNorm		NormFinder			
						Across Samples		Grouped by BTV Status	
		Rank	*r* (SD)	Rank	M Index	Rank	Stability	Rank	Stability
CytB5	24.6 (0.28)	3	1.0 (0.49)	1	0.105	4	0.151	2	0.063
RpL13	23.4 (0.23)	2	1.0 (0.36)	3	0.148	1	0.023	1	0.060
RpL21	22.2 (0.18)	1	1.0 (0.29)	5	0.177	2	0.023	4	0.076
RpS8	22.0 (0.27)	5	0.98 (0.58)	2	0.105	5	0.185	5	0.086
V-ATPase	26.2 (0.34)	4	0.99 (0.53)	4	0.153	3	0.044	3	0.078

Table S4. Primers forward (F), reverse (R) and hydrolysis probe (P) sequences used for RT-qPCR analysis of the expression levels of *ski2*, *gst*, *gst-1* and *Toll*-like.

Gene	Accession No.		Primer (5'-3')	Amplicon Size (bp)	Amplification Efficiency % (R^2)
Toll-like	XLOC_000522	F	GGCTGACATTGAGGGTGATTTC	99	94.5 (0.99)
		R	CGTCCCAAATGAACATGCTTG		
		P	TGAACCCTCGCATTGGAAAACCTCTCG		
gst	CSON010973	F	CTCAACACAGCGTTCCAAC	81	92.7 (0.98)
		R	TGAGGCTCCATTTGGAACTG		
		P	CTGACCGAGTCCAAGGCCATTTTGGGA		
gst-1	CSON011559	F	TGCCGATTACTGGTATCCTCAA	127	93.8 (0.98)
		R	CCCATTGCTTCTTCCATTTTCT		
		P	TTTGGCAAACAACCCGCAAATCCA		
ski2	XLOC_006435	F	TGAGACCACCCAAAATGCAG	101	96.8 (0.99)
		R	AAATGACCCCATCGCAATCG		
		P	TGCGTGGTCCTGAAACAGTCCACAACA		

Table S5. Difference of expression levels in four genes (*Toll*-like, *gst*, *gst-1* and *ski2*) between vector-competent and vector-refractory females assessed by RNAseq and RT-qPCR. * denote a difference in expression between refractory and competent females with a $P < 0.05$ (RT-qPCR significance estimated using a Welch Two Sample t-test with d.f. = 1).

**Gene**	**Log-2 Fold Change Refractory vs Competent**	
	**RNASeq**	**RT-qPCR [mean (+/- SE)]**
Toll-like	1.21*	0.89 (0.36)
gst	2.08*	2.02 (0.17)
gst-1	-1.07*	-0.93 (0.15)
ski2	-1.10*	-0.9 (-0.19)

Table S6. Orthology analysis of two of genes *Ski2* **(a)** and *gst-1* **(b)**.

a)

Class	Order	suborder	Family	Species	1 to 1	1 to many	many to many
Insecta	Diptera	Brachycera	Muscidae	*Musca domestica*	1	0	0
Insecta	Diptera	Brachycera	Muscidae	*Stomoxys calcitrans*	1	0	0
Insecta	Diptera	Brachycera	Drosophilidae	*Drosophila melanogaster*	1	0	0
Insecta	Diptera	Brachycera	Glossinidae	*Glossina austeni*	1	0	0
Insecta	Diptera	Brachycera	Glossinidae	*Glossina brevipalpis*	1	0	0
Insecta	Diptera	Brachycera	Glossinidae	*Glossina fuscipes*	1	0	0
Insecta	Diptera	Brachycera	Glossinidae	*Glossina pallidipes*	1	0	0
Insecta	Diptera	Brachycera	Glossinidae	*Glossina palpalis*	1	0	0
Insecta	Diptera	Nematocera	Culicidae	*Aedes aegypti*	1	0	0
Insecta	Diptera	Nematocera	Culicidae	*Aedes albopictus*	1	0	0
Insecta	Diptera	Nematocera	Culicidae	*Anopheles albimanus*	1	0	0
Insecta	Diptera	Nematocera	Culicidae	*Anopheles arabiensis*	1	0	0
Insecta	Diptera	Nematocera	Culicidae	*Anopheles atroparvus*	1	0	0
Insecta	Diptera	Nematocera	Culicidae	*Anopheles christyi*	1	0	0
Insecta	Diptera	Nematocera	Culicidae	*Anopheles coluzzii*	1	0	0
Insecta	Diptera	Nematocera	Culicidae	*Anopheles culicifacies*	1	0	0
Insecta	Diptera	Nematocera	Culicidae	*Anopheles darlingi*	1	0	0
Insecta	Diptera	Nematocera	Culicidae	*Anopheles dirus*	1	0	0
Insecta	Diptera	Nematocera	Culicidae	*Anopheles epiroticus*	1	0	0
Insecta	Diptera	Nematocera	Culicidae	*Anopheles farauti*	1	0	0
Insecta	Diptera	Nematocera	Culicidae	*Anopheles funestus*	1	0	0
Insecta	Diptera	Nematocera	Culicidae	*Anopheles gambiae*	1	0	0
Insecta	Diptera	Nematocera	Culicidae	*Anopheles maculatus*	0	2	0
Insecta	Diptera	Nematocera	Culicidae	*Anopheles melas*	1	0	0
Insecta	Diptera	Nematocera	Culicidae	*Anopheles merus*	1	0	0

Insecta	Diptera	Nematocera	Culicidae	*Anopheles minimus*	1	0	0
Insecta	Diptera	Nematocera	Culicidae	*Anopheles quadriannulatus*	1	0	0
Insecta	Diptera	Nematocera	Culicidae	*Anopheles sinensis*	1	0	0
Insecta	Diptera	Nematocera	Culicidae	*Anopheles stephensi*	1	0	0
Insecta	Diptera	Nematocera	Psychodidae	*Phlebotomus papatasi*	1	0	0
Insecta	Hemiptera		Cimicidae	*Cimex lectularius*	1	0	0
Insecta	Phthiraptera		Pediculidae	*Pediculus humanus*	1	0	0
Insecta	Hemiptera		Reduviidae	*Rhodnius prolixus*	1	0	0
Gastropoda			Planorbidae	*Biomphalaria glabrata*	1	0	0
Arachnida	Ixodida		Ixodidae	*Ixodes_scapularis*	1	0	0
Arachnida	Astigmata		Sarcoptidae	*Sarcoptes scabiei*	1	0	0

b)

**Class**	**Order**	**suborder**	**Family**	**Species**	**1 to 1**	**1 to many**	**many to many**
Insecta	Diptera	Brachycera	Drosophilidae	*Drosophila melanogaster*	0	0	10
Insecta	Diptera	Brachycera	Glossinidae	*Glossina austeni*	0	0	2
Insecta	Diptera	Brachycera	Glossinidae	*Glossina brevipalpis*	1	0	0
Insecta	Diptera	Brachycera	Glossinidae	*Glossina fuscipes*	1	0	0
Insecta	Diptera	Brachycera	Glossinidae	*Glossina morsitans*	1	0	0
Insecta	Diptera	Brachycera	Glossinidae	*Glossina pallidipes*	1	0	0
Insecta	Diptera	Brachycera	Glossinidae	*Glossina palpalis*	1	0	0
Insecta	Diptera	Brachycera	Muscidae	*Musca domestica*	0	0	6
Insecta	Diptera	Brachycera	Muscidae	*Stomoxys calcitrans*	0	0	16
Insecta	Diptera	Nematocera	Culicidae	*Aedes aegypti*	1	0	0
Insecta	Diptera	Nematocera	Culicidae	*Aedes albopictus*	1	0	0
Insecta	Diptera	Nematocera	Culicidae	*Anopheles albimanus*	1	0	0
Insecta	Diptera	Nematocera	Culicidae	*Anopheles arabiensis*	1	0	0
Insecta	Diptera	Nematocera	Culicidae	*Anopheles atroparvus*	1	0	0

Insecta	Diptera	Nematocera	Culicidae	*Anopheles christyi*	1	0	0
Insecta	Diptera	Nematocera	Culicidae	*Anopheles coluzzii*	1	0	0
Insecta	Diptera	Nematocera	Culicidae	*Anopheles culicifacies*	1	0	0
Insecta	Diptera	Nematocera	Culicidae	*Anopheles darlingi*	0	2	0
Insecta	Diptera	Nematocera	Culicidae	*Anopheles epiroticus*	1	0	0
Insecta	Diptera	Nematocera	Culicidae	*Anopheles farauti*	1	0	0
Insecta	Diptera	Nematocera	Culicidae	*Anopheles funestus*	1	0	0
Insecta	Diptera	Nematocera	Culicidae	*Anopheles maculatus*	1	0	0
Insecta	Diptera	Nematocera	Culicidae	*Anopheles melas*	1	0	0
Insecta	Diptera	Nematocera	Culicidae	*Anopheles merus*	1	0	0
Insecta	Diptera	Nematocera	Culicidae	*Anopheles minimus*	1	0	0
Insecta	Diptera	Nematocera	Culicidae	*Anopheles quadriannulatus*	1	0	0
Insecta	Diptera	Nematocera	Culicidae	*Anopheles sinensis*	1	0	0
Insecta	Diptera	Nematocera	Culicidae	*Anopheles stephensi*	1	0	0
Insecta	Diptera	Nematocera	Culicidae	*Culex quinquefasciatus*	1	0	0
Insecta	Hemiptera		Cimicidae	*Cimex lectularius*	0	1	0
Insecta	Phthiraptera		Pediculidae	*Pediculus humanus*	0	1	0

Table S7. Immune pathways genes identified in *C. sonorensis* using the Ensembl Compara pipeline and the *D. melanogaster* genes as reference. **a)** Toll pathway; **b)** Imd pathway; **c)** Jak/Stat pathway.

a)

FlyBase gene ID	FlyBase Gene name	*C. sonorensis* gene
FBgn0000533	easter	CSON000242, CSON007646, CSON015050
FBgn0022787	Helicase 89B	CSON011086
FBgn0010441	pelle	CSON013584, CSON013585
FBgn0035056	spatzle 6	CSON000834
FBgn0261526	Neurotrophin 1	CSON002435, CSON010037
FBgn0000250	cactus	CSON002111
FBgn0025574	Pellino	CSON005146
FBgn0033402	Myd88	CSON001312, CSON010245
FBgn0028984	Serpin 88Ea	CSON001726, CSON013093
FBgn0028990	Serpin 27A	CSON002129, CSON002306, CSON002307, CSON004516, CSON006573, CSON007761, CSON009137, CSON009361, CSON010698
FBgn0011274	Dif	CSON012766, CSON015181
FBgn0260632	dorsal	CSON012766, CSON015181
FBgn0003089	pipe	CSON000657, CSON007508
FBgn0039102	Spatzle-Processing Enzyme	n.a.
FBgn0010602	lesswright	CSON002096
FBgn0036448	myopic	CSON009406
FBgn0004003	windbeutel	CSON012906, CSON015574
FBgn0031450	Hepatocyte growth factor regulated tyrosine kinase substrate	CSON009740
FBgn0032362	spatzle 4	CSON011800
FBgn0010269	Downstream of raf1	CSON001704, CSON008017
FBgn0014018	Relish	CSON014412
FBgn0031959	spatzle 3	CSON007138
FBgn0030774	spheroide	CSON004605, CSON005566, CSON008302, CSON009338, CSON009345, CSON009526, CSON012078, CSON013394, CSON015385
FBgn0039494	Gram-positive Specific Serine protease	CSON003275
FBgn0028436	ECSIT	n.a.
FBgn0026760	Tehao	CSON001282, CSON001790, CSON007335, CSON011712
FBgn0262473	Toll	CSON001282, CSON001790, CSON007335, CSON011712
FBgn0041205	kenny	CSON000146

FBgn0002926	nudel	CSON004712, CSON008920, CSON014457
FBgn0001990	weckle	CSON001842, CSON005192
FBgn0261988	G protein-coupled receptor kinase 2	CSON003998
FBgn0030051	Serine Protease Immune Response Integrator	n.a.
FBgn0030926	persephone	CSON008961, CSON008962, CSON009227, CSON010323, CSON011404, CSON015270, CSON015271
FBgn0003495	spatzle	CSON002624, CSON002625, CSON005603, CSON005604, CSON011604
FBgn0002930	necrotic	CSON010106
FBgn0003450	snake	CSON005854, CSON011813
FBgn0035379	spatzle 5	CSON014426
FBgn0030310	Peptidoglycan recognition protein SA	n.a.
FBgn0265464	TNF-receptor-associated factor 6	n.a.
FBgn0003882	tube	n.a.
FBgn0000808	gastrulation-defective	CSON009297
FBgn0024222	immune response deficient 5	CSON001226

b)

**FlyBase gene ID**	**FlyBase Gene name**	***C. sonorensis* gene**
FBgn0051410	Niemann-Pick type C-2e	CSON000565, CSON001914, CSON003895, CSON003925, CSON004032, CSON007608, CSON008026, CSON008571, CSON013231
FBgn0038928	Fas-associated death domain ortholog	n.a.
FBgn0086358	TAK1-associated binding protein 2	CSON004331, CSON012364
FBgn0031381	Niemann-Pick type C-2a	CSON004270, CSON004915
FBgn0035976	Peptidoglycan recognition protein LC	CSON003218, CSON008584
FBgn0035977	Peptidoglycan recognition protein LF	CSON003218, CSON008584
FBgn0015247	Death-associated inhibitor of apoptosis 2	CSON007331, CSON010539
FBgn0013983	immune deficiency	n.a.
FBgn0020381	Death related ced-3/Nedd2-like caspase	CSON010543, CSON010544, CSON012690
FBgn0030695	Peptidoglycan recognition protein LE	n.a.
FBgn0014018	Relish	CSON014412

c)

**FlyBase gene ID**	**FlyBase Gene name**	***C. sonorensis* gene**
FBgn0004107	Cyclin-dependent kinase 2	CSON006415
FBgn0016917	Signal-transducer and activator of transcription protein at 92E	CSON006720, CSON012361, CSON013044
FBgn0010315	Cyclin D	n.a.
FBgn0016131	Cyclin-dependent kinase 4	n.a.
FBgn0004956	unpaired 1	CSON004570
FBgn0053542	unpaired 3	CSON004570
FBgn0043903	domeless	CSON004107, CSON004944, CSON004949
FBgn0027363	Signal transducing adaptor molecule	CSON006278
FBgn0041184	Suppressor of cytokine signaling at 36E	CSON012903
FBgn0010382	Cyclin E	CSON012774
FBgn0262114	Ran-binding protein M	n.a.
FBgn0004864	hopscotch	CSON002696, CSON003569, CSON007500, CSON014132
FBgn0030904	unpaired 2	CSON004570

Table S8. Homologue immune genes in several invertebrate species identified using *D. melanogaster* genes as reference all with InterPro and GO terms associated with Toll **(a)**, Imd **(b)** and Jak/Stat **(c)**, in the Ensembl Compara pipeline.

a)

**FlyBase gene ID**	**FlyBase Gene name**	**Compara family**
FBgn0000533	easter	aedes_aegypti,AAEL005064 anopheles_gambiae,AGAP004148 belgica_antarctica,IU25_11170 culex_quinquefasciatus,CPIJ009625 *culicoides_sonorensis,CSON000242 culicoides_sonorensis,CSON007646 culicoides_sonorensis,CSON015050* drosophila_melanogaster,FBgn0000533 glossina_morsitans,GMOY002535 lucilia_cuprina,FF38_04952 lutzomyia_longipalpis,LLOJ001834 musca_domestica,MDOA011344 phlebotomus_papatasi,PPAI007024 stomoxys_calcitrans,SCAU003952
FBgn0022787	Helicase 89B	acyrthosiphon_pisum,ACYPI068681 aedes_aegypti,AAEL013189 anopheles_gambiae,AGAP001820 belgica_antarctica,IU25_01389 culex_quinquefasciatus,CPIJ003582 culicoides_sonorensis,CSON011086 drosophila_melanogaster,FBgn0022787 glossina_morsitans,GMOY011475 lucilia_cuprina,FF38_04397 lutzomyia_longipalpis,LLOJ009858 musca_domestica,MDOA006474 pediculus_humanus,PHUM422980 phlebotomus_papatasi,PPAI005580 rhodnius_prolixus,RPRC007719 sarcoptes_scabiei,SSCA001379 stomoxys_calcitrans,SCAU009956 tetranychus_urticae,tetur08g05200
FBgn0010441	pelle	*acyrthosiphon_pisum,ACYPI000858 acyrthosiphon_pisum,ACYPI006580* acyrthosiphon_pisum,ACYPI009928 aedes_aegypti,AAEL006571 aedes_aegypti,AAEL007642 anopheles_gambiae,AGAP002966 anopheles_gambiae,AGAP003062 belgica_antarctica,IU25_03336 *culex_quinquefasciatus,CPIJ013746 culex_quinquefasciatus,CPIJ015474 culicoides_sonorensis,CSON013584 culicoides_sonorensis,CSON013585* drosophila_melanogaster,FBgn0010441 glossina_morsitans,GMOY000628 lucilia_cuprina,FF38_00242 *lucilia_cuprina,FF38_10038* lutzomyia_longipalpis,LLOJ001101 musca_domestica,MDOA009915 pediculus_humanus,PHUM194370 *pediculus_humanus,PHUM518290* phlebotomus_papatasi,PPAI007366 *rhodnius_prolixus,RPRC005685 rhodnius_prolixus,RPRC005871* sarcoptes_scabiei,SSCA009509 stomoxys_calcitrans,SCAU006171 *tetranychus_urticae,tetur01g00840 tetranychus_urticae,tetur03g06520*
FBgn0035056	spatzle 6	acyrthosiphon_pisum,ACYPI001990 aedes_aegypti,AAEL012164 anopheles_gambiae,AGAP005126 belgica_antarctica,IU25_05244 culex_quinquefasciatus,CPIJ002281 culicoides_sonorensis,CSON000834 drosophila_melanogaster,FBgn0035056 glossina_morsitans,GMOY010883 lucilia_cuprina,FF38_11512 lutzomyia_longipalpis,LLOJ002272 musca_domestica,MDOA007158 pediculus_humanus,PHUM202070 phlebotomus_papatasi,PPAI009854 rhodnius_prolixus,RPRC000952 *sarcoptes_scabiei,SSCA002719 sarcoptes_scabiei,SSCA004561* stomoxys_calcitrans,SCAU013453 tetranychus_urticae,tetur11g00320
FBgn0261526	Neurotrophin 1	acyrthosiphon_pisum,ACYPI003414 aedes_aegypti,AAEL001435 anopheles_gambiae,AGAP006483 belgica_antarctica,IU25_01464 *culex_quinquefasciatus,CPIJ000272 culex_quinquefasciatus,CPIJ000273 culicoides_sonorensis,CSON002435 culicoides_sonorensis,CSON010037* drosophila_melanogaster,FBgn0261526 glossina_morsitans,GMOY002930 lucilia_cuprina,FF38_05105 *lutzomyia_longipalpis,LLOJ007065 lutzomyia_longipalpis,LLOJ007459* musca_domestica,MDOA007929 pediculus_humanus,PHUM057390 phlebotomus_papatasi,PPAI003677

		rhodnius_prolixus,RPRC013110 sarcoptes_scabiei,SSCA007644 stomoxys_calcitrans,SCAU014679 tetranychus_urticae,tetur11g04440
FBgn0000250	cactus	acyrthosiphon_pisum,ACYPI006820 aedes_aegypti,AAEL000709 anopheles_gambiae,AGAP007938 belgica_antarctica,IU25_11072 culex_quinquefasciatus,CPIJ004774 culicoides_sonorensis,CSON002111 drosophila_melanogaster,FBgn0000250 *glossina_morsitans,GMOY007166 glossina_morsitans,GMOY008201 glossina_morsitans,GMOY008203* lucilia_cuprina,FF38_08033 lutzomyia_longipalpis,LLOJ004612 musca_domestica,MDOA013202 pediculus_humanus,PHUM345810 *phlebotomus_papatasi,PPAI010893 phlebotomus_papatasi,PPAI010894* rhodnius_prolixus,RPRC017349 sarcoptes_scabiei,SSCA002758 stomoxys_calcitrans,SCAU015686 tetranychus_urticae,tetur14g01540
FBgn0025574	Pellino	acyrthosiphon_pisum,ACYPI083717 aedes_aegypti,AAEL002420 anopheles_gambiae,AGAP004232 belgica_antarctica,IU25_11616 culex_quinquefasciatus,CPIJ008269 culicoides_sonorensis,CSON005146 drosophila_melanogaster,FBgn0025574 glossina_morsitans,GMOY007135 lucilia_cuprina,FF38_11637 lutzomyia_longipalpis,LLOJ004972 musca_domestica,MDOA003335 pediculus_humanus,PHUM235930 phlebotomus_papatasi,PPAI007913 rhodnius_prolixus,RPRC011811 sarcoptes_scabiei,SSCA010501 stomoxys_calcitrans,SCAU003666 tetranychus_urticae,tetur07g04160
FBgn0033402	Myd88	acyrthosiphon_pisum,ACYPI001638 aedes_aegypti,AAEL007768 anopheles_gambiae,AGAP005252 belgica_antarctica,IU25_11425 *culex_quinquefasciatus,CPIJ008547 culex_quinquefasciatus,CPIJ018307 culicoides_sonorensis,CSON001312 culicoides_sonorensis,CSON010245* drosophila_melanogaster,FBgn0033402 glossina_morsitans,GMOY005784 *lucilia_cuprina,FF38_02263 lucilia_cuprina,FF38_05343* lutzomyia_longipalpis,LLOJ009628 musca_domestica,MDOA005925 pediculus_humanus,PHUM536290 phlebotomus_papatasi,PPAI006982 rhodnius_prolixus,RPRC009785 sarcoptes_scabiei,SSCA003014 stomoxys_calcitrans,SCAU010762 tetranychus_urticae,tetur11g01630
FBgn0028984	Serpin 88Ea	*acyrthosiphon_pisum,ACYPI004516 acyrthosiphon_pisum,ACYPI30247* aedes_aegypti,AAEL008364 *anopheles_gambiae,AGAP003139 anopheles_gambiae,AGAP012938* belgica_antarctica,IU25_05425 culex_quinquefasciatus,CPIJ011775 *culicoides_sonorensis,CSON001726 culicoides_sonorensis,CSON013093 drosophila_melanogaster,FBgn0028984 drosophila_melanogaster,FBgn0038299 glossina_morsitans,GMOY000990 glossina_morsitans,GMOY013035* lucilia_cuprina,FF38_13018 lutzomyia_longipalpis,LLOJ001775 *musca_domestica,MDOA000087 musca_domestica,MDOA007964* pediculus_humanus,PHUM075870 phlebotomus_papatasi,PPAI005147 rhodnius_prolixus,RPRC002795 sarcoptes_scabiei,SSCA004162 stomoxys_calcitrans,SCAU006100 *tetranychus_urticae,tetur05g01350 tetranychus_urticae,tetur35g00950*
FBgn0028990	Serpin 27A	*acyrthosiphon_pisum,ACYPI000180 acyrthosiphon_pisum,ACYPI001790 acyrthosiphon_pisum,ACYPI008897 aedes_aegypti,AAEL003653 aedes_aegypti,AAEL003686 aedes_aegypti,AAEL003697 aedes_aegypti,AAEL005665 aedes_aegypti,AAEL005670 aedes_aegypti,AAEL005673 aedes_aegypti,AAEL010769 aedes_aegypti,AAEL011777 aedes_aegypti,AAEL013936 aedes_aegypti,AAEL014078 aedes_aegypti,AAEL014079 aedes_aegypti,AAEL014138 aedes_aegypti,AAEL014141 aedes_aegypti,AAEL014980 anopheles_gambiae,AGAP001375 anopheles_gambiae,AGAP001376 anopheles_gambiae,AGAP001377 anopheles_gambiae,AGAP003194 anopheles_gambiae,AGAP006909 anopheles_gambiae,AGAP006910 anopheles_gambiae,AGAP006911 anopheles_gambiae,AGAP009212 anopheles_gambiae,AGAP009213 anopheles_gambiae,AGAP009221 anopheles_gambiae,AGAP009670*

		belgica_antarctica,IU25_01636 belgica_antarctica,IU25_01637 belgica_antarctica,IU25_01638 belgica_antarctica,IU25_04210 belgica_antarctica,IU25_05339 belgica_antarctica,IU25_06305 culex_quinquefasciatus,CPIJ009131 culex_quinquefasciatus,CPIJ009132 culex_quinquefasciatus,CPIJ009134 culex_quinquefasciatus,CPIJ009135 culex_quinquefasciatus,CPIJ011718 culex_quinquefasciatus,CPIJ011719 culex_quinquefasciatus,CPIJ012013 culex_quinquefasciatus,CPIJ012016 culex_quinquefasciatus,CPIJ013205 culex_quinquefasciatus,CPIJ016296 culex_quinquefasciatus,CPIJ016297 culex_quinquefasciatus,CPIJ016298 culex_quinquefasciatus,CPIJ016300 culex_quinquefasciatus,CPIJ016301 culex_quinquefasciatus,CPIJ016697 culex_quinquefasciatus,CPIJ016698 culex_quinquefasciatus,CPIJ016699 culicoides_sonorensis,CSON002129 culicoides_sonorensis,CSON002306 culicoides_sonorensis,CSON002307 culicoides_sonorensis,CSON004516 culicoides_sonorensis,CSON006573 culicoides_sonorensis,CSON007761 culicoides_sonorensis,CSON009137 culicoides_sonorensis,CSON009361 culicoides_sonorensis,CSON010698 drosophila_melanogaster,FBgn0028990 drosophila_melanogaster,FBgn0031973 drosophila_melanogaster,FBgn0036969 drosophila_melanogaster,FBgn0036970 drosophila_melanogaster,FBgn0039795 drosophila_melanogaster,FBgn0262057 glossina_morsitans,GMOY000930 glossina_morsitans,GMOY002262 glossina_morsitans,GMOY003382 glossina_morsitans,GMOY008942 lucilia_cuprina,FF38_00567 lucilia_cuprina,FF38_01866 lucilia_cuprina,FF38_02248 lucilia_cuprina,FF38_10172 lutzomyia_longipalpis,LLOJ001538 lutzomyia_longipalpis,LLOJ003123 lutzomyia_longipalpis,LLOJ006402 lutzomyia_longipalpis,LLOJ006403 lutzomyia_longipalpis,LLOJ006962 lutzomyia_longipalpis,LLOJ008279 lutzomyia_longipalpis,LLOJ008570 musca_domestica,MDOA003000 musca_domestica,MDOA006189 musca_domestica,MDOA006216 musca_domestica,MDOA007461 musca_domestica,MDOA011845 pediculus_humanus,PHUM106570 pediculus_humanus,PHUM106690 pediculus_humanus,PHUM291170 pediculus_humanus,PHUM291180 pediculus_humanus,PHUM291190 pediculus_humanus,PHUM291200 pediculus_humanus,PHUM432060 pediculus_humanus,PHUM492620 phlebotomus_papatasi,PPAI003938 phlebotomus_papatasi,PPAI007901 phlebotomus_papatasi,PPAI008529 rhodnius_prolixus,RPRC004932 rhodnius_prolixus,RPRC012557 sarcoptes_scabiei,SSCA006474 *stomoxys_calcitrans,SCAU001616 stomoxys_calcitrans,SCAU003847 stomoxys_calcitrans,SCAU003866 stomoxys_calcitrans,SCAU007132 stomoxys_calcitrans,SCAU014806 stomoxys_calcitrans,SCAU015747* tetranychus_urticae,tetur02g03240
FBgn0011274	Dorsal-related immunity factor	*acyrthosiphon_pisum,ACYPI003588 acyrthosiphon_pisum,ACYPI005133 aedes_aegypti,AAEL006930 aedes_aegypti,AAEL007696* anopheles_gambiae,AGAP009515 belgica_antarctica,IU25_05800 *culex_quinquefasciatus,CPIJ002469 culex_quinquefasciatus,CPIJ015741 culex_quinquefasciatus,CPIJ019376 culicoides_sonorensis,CSON012766 culicoides_sonorensis,CSON015181 drosophila_melanogaster,FBgn0011274 drosophila_melanogaster,FBgn0260632 glossina_morsitans,GMOY004477 glossina_morsitans,GMOY004479 glossina_morsitans,GMOY008557 glossina_morsitans,GMOY011793 glossina_morsitans,GMOY011794 glossina_morsitans,GMOY011795 glossina_morsitans,GMOY011796 lucilia_cuprina,FF38_08307 lucilia_cuprina,FF38_08314 musca_domestica,MDOA008285 musca_domestica,MDOA012390* pediculus_humanus,PHUM534140 phlebotomus_papatasi,PPAI001149 *rhodnius_prolixus,RPRC001614 rhodnius_prolixus,RPRC003790*

		sarcoptes_scabiei,SSCA002663 *stomoxys_calcitrans,SCAU008769 stomoxys_calcitrans,SCAU010056* tetranychus_urticae,tetur11g04270
FBgn0260632	dorsal	acyrthosiphon_pisum,ACYPI003588 acyrthosiphon_pisum,ACYPI005133 *aedes_aegypti,AAEL006930 aedes_aegypti,AAEL007696* anopheles_gambiae,AGAP009515 belgica_antarctica,IU25_05800 *culex_quinquefasciatus,CPIJ002469 culex_quinquefasciatus,CPIJ015741 culex_quinquefasciatus,CPIJ019376 culicoides_sonorensis,CSON012766 culicoides_sonorensis,CSON015181 drosophila_melanogaster,FBgn0011274 drosophila_melanogaster,FBgn0260632 glossina_morsitans,GMOY004477 glossina_morsitans,GMOY004479 glossina_morsitans,GMOY008557 glossina_morsitans,GMOY011793 glossina_morsitans,GMOY011794 glossina_morsitans,GMOY011795 glossina_morsitans,GMOY011796 lucilia_cuprina,FF38_08307 lucilia_cuprina,FF38_08314 musca_domestica,MDOA008285 musca_domestica,MDOA012390* pediculus_humanus,PHUM534140 phlebotomus_papatasi,PPAI001149 *rhodnius_prolixus,RPRC001614 rhodnius_prolixus,RPRC003790* sarcoptes_scabiei,SSCA002663 *stomoxys_calcitrans,SCAU008769 stomoxys_calcitrans,SCAU010056* tetranychus_urticae,tetur11g04270
FBgn0003089	pipe	acyrthosiphon_pisum,ACYPI008454 aedes_aegypti,AAEL006219 anopheles_gambiae,AGAP006058 belgica_antarctica,IU25_11723 culex_quinquefasciatus,CPIJ000310 *culicoides_sonorensis,CSON000657 culicoides_sonorensis,CSON007508* drosophila_melanogaster,FBgn0003089 glossina_morsitans,GMOY011209 lucilia_cuprina,FF38_09288 lutzomyia_longipalpis,LLOJ001433 *musca_domestica,MDOA001206 musca_domestica,MDOA009543* pediculus_humanus,PHUM457510 phlebotomus_papatasi,PPAI002814 rhodnius_prolixus,RPRC005278 *stomoxys_calcitrans,SCAU002981 stomoxys_calcitrans,SCAU004252*
FBgn0039102	Spatzle-Processing Enzyme	drosophila_melanogaster,FBgn0039102 lucilia_cuprina,FF38_03611 *musca_domestica,MDOA000602 musca_domestica,MDOA001121 musca_domestica,MDOA006410 musca_domestica,MDOA006757 musca_domestica,MDOA012499* stomoxys_calcitrans,SCAU005252 stomoxys_calcitrans,SCAU016275
FBgn0010602	lesswright	*acyrthosiphon_pisum,ACYPI000084 acyrthosiphon_pisum,ACYPI003179 acyrthosiphon_pisum,ACYPI007149 acyrthosiphon_pisum,ACYPI072914 acyrthosiphon_pisum,ACYPI35813 aedes_aegypti,AAEL007477 aedes_aegypti,AAEL009770* anopheles_gambiae,AGAP011076 anopheles_gambiae,AGAP012107 belgica_antarctica,IU25_05384 culex_quinquefasciatus,CPIJ005149 culicoides_sonorensis,CSON002096 drosophila_melanogaster,FBgn0010602 glossina_morsitans,GMOY007017 lucilia_cuprina,FF38_06939 lutzomyia_longipalpis,LLOJ004518 musca_domestica,MDOA014107 pediculus_humanus,PHUM274670 rhodnius_prolixus,RPRC006315 sarcoptes_scabiei,SSCA005483 stomoxys_calcitrans,SCAU014606 tetranychus_urticae,tetur14g01920
FBgn0036448	myopic	acyrthosiphon_pisum,ACYPI49993 aedes_aegypti,AAEL018205 anopheles_gambiae,AGAP011586 belgica_antarctica,IU25_01460 culex_quinquefasciatus,CPIJ011610 culicoides_sonorensis,CSON009406 drosophila_melanogaster,FBgn0036448 glossina_morsitans,GMOY011192 lucilia_cuprina,FF38_03257 lutzomyia_longipalpis,LLOJ004722 musca_domestica,MDOA008898 pediculus_humanus,PHUM135870 rhodnius_prolixus,RPRC001976 sarcoptes_scabiei,SSCA006415 stomoxys_calcitrans,SCAU007847 tetranychus_urticae,tetur36g01240
FBgn0004003	windbeutel	acyrthosiphon_pisum,ACYPI000995 aedes_aegypti,AAEL014052 anopheles_gambiae,AGAP000395 belgica_antarctica,IU25_03275 culex_quinquefasciatus,CPIJ009432 *culicoides_sonorensis,CSON012906 culicoides_sonorensis,CSON015574* drosophila_melanogaster,FBgn0004003 glossina_morsitans,GMOY009097 lucilia_cuprina,FF38_09332 lutzomyia_longipalpis,LLOJ009844 musca_domestica,MDOA007436

		pediculus_humanus,PHUM260550 phlebotomus_papatasi,PPAI010839 rhodnius_prolixus,RPRC007848 stomoxys_calcitrans,SCAU006471 tetranychus_urticae,tetur01g00720
FBgn0031450	Hepatocyte growth factor regulated tyrosine kinase substrate	*acyrthosiphon_pisum,ACYPI006279 acyrthosiphon_pisum,ACYPI087535* aedes_aegypti,AAEL005339 anopheles_gambiae,AGAP008887 culex_quinquefasciatus,CPIJ001538 culicoides_sonorensis,CSON009740 drosophila_melanogaster,FBgn0031450 glossina_morsitans,GMOY003119 lucilia_cuprina,FF38_05821 lutzomyia_longipalpis,LLOJ005882 musca_domestica,MDOA005074 pediculus_humanus,PHUM430260 phlebotomus_papatasi,PPAI010889 *rhodnius_prolixus,RPRC001853 rhodnius_prolixus,RPRC013322* sarcoptes_scabiei,SSCA006622 stomoxys_calcitrans,SCAU010252 tetranychus_urticae,tetur09g02340
FBgn0032362	spatzle 4	acyrthosiphon_pisum,ACYPI006811 aedes_aegypti,AAEL007897 anopheles_gambiae,AGAP007866 belgica_antarctica,IU25_03945 culex_quinquefasciatus,CPIJ015335 culicoides_sonorensis,CSON011800 drosophila_melanogaster,FBgn0032362 lucilia_cuprina,FF38_08270 lutzomyia_longipalpis,LLOJ009476 musca_domestica,MDOA015318 pediculus_humanus,PHUM190080 phlebotomus_papatasi,PPAI001890 rhodnius_prolixus,RPRC010774 sarcoptes_scabiei,SSCA008049 stomoxys_calcitrans,SCAU015808 tetranychus_urticae,tetur10g05070
FBgn0010269	Downstream of raf1	acyrthosiphon_pisum,ACYPI000770 aedes_aegypti,AAEL012723 anopheles_gambiae,AGAP001103 belgica_antarctica,IU25_00290 culex_quinquefasciatus,CPIJ015801 *culicoides_sonorensis,CSON001704 culicoides_sonorensis,CSON008017* drosophila_melanogaster,FBgn0010269 lucilia_cuprina,FF38_11249 lutzomyia_longipalpis,LLOJ010497 *musca_domestica,MDOA002881 musca_domestica,MDOA003086* pediculus_humanus,PHUM413460 *phlebotomus_papatasi,PPAI009597 phlebotomus_papatasi,PPAI009598* rhodnius_prolixus,RPRC002914 sarcoptes_scabiei,SSCA010469 stomoxys_calcitrans,SCAU007392 tetranychus_urticae,tetur17g02600
FBgn0014018	Relish	aedes_aegypti,AAEL007624 anopheles_gambiae,AGAP006747 belgica_antarctica,IU25_06473 culex_quinquefasciatus,CPIJ012236 culicoides_sonorensis,CSON014412 drosophila_melanogaster,FBgn0014018 glossina_morsitans,GMOY013090 lucilia_cuprina,FF38_04187 *lutzomyia_longipalpis,LLOJ002097 lutzomyia_longipalpis,LLOJ002098* musca_domestica,MDOA004895 pediculus_humanus,PHUM424590 phlebotomus_papatasi,PPAI012820 rhodnius_prolixus,RPRC017358 sarcoptes_scabiei,SSCA006430 stomoxys_calcitrans,SCAU011881 tetranychus_urticae,tetur07g00650
FBgn0031959	spatzle 3	*acyrthosiphon_pisum,ACYPI085929 acyrthosiphon_pisum,ACYPI55738 aedes_aegypti,AAEL008596 aedes_aegypti,AAEL014950* anopheles_gambiae,AGAP008360 culex_quinquefasciatus,CPIJ001752 culicoides_sonorensis,CSON007138 drosophila_melanogaster,FBgn0031959 glossina_morsitans,GMOY004908 lucilia_cuprina,FF38_14313 lutzomyia_longipalpis,LLOJ008474 musca_domestica,MDOA002454 pediculus_humanus,PHUM332090 phlebotomus_papatasi,PPAI001447 rhodnius_prolixus,RPRC001351 stomoxys_calcitrans,SCAU006695
FBgn0030774	spheroide	*aedes_aegypti,AAEL001674 aedes_aegypti,AAEL001690 aedes_aegypti,AAEL001693 aedes_aegypti,AAEL001701 aedes_aegypti,AAEL001703 aedes_aegypti,AAEL002347 aedes_aegypti,AAEL002360 aedes_aegypti,AAEL006594 aedes_aegypti,AAEL006598 aedes_aegypti,AAEL008567 aedes_aegypti,AAEL008769 aedes_aegypti,AAEL008780 aedes_aegypti,AAEL008781 aedes_aegypti,AAEL009244 aedes_aegypti,AAEL009680 aedes_aegypti,AAEL017457 anopheles_gambiae,AGAP005065 anopheles_gambiae,AGAP005194 anopheles_gambiae,AGAP005195 anopheles_gambiae,AGAP005196*

		anopheles_gambiae,AGAP005310 anopheles_gambiae,AGAP006416 anopheles_gambiae,AGAP006707 anopheles_gambiae,AGAP006709 anopheles_gambiae,AGAP006710 anopheles_gambiae,AGAP006711 anopheles_gambiae,AGAP007141 anopheles_gambiae,AGAP007142 anopheles_gambiae,AGAP009828 belgica_antarctica,IU25_00157 belgica_antarctica,IU25_08491 belgica_antarctica,IU25_08729 belgica_antarctica,IU25_10346 culex_quinquefasciatus,CPIJ002518 culex_quinquefasciatus,CPIJ003915 culex_quinquefasciatus,CPIJ006568 culex_quinquefasciatus,CPIJ007838 culex_quinquefasciatus,CPIJ008524 culex_quinquefasciatus,CPIJ015161 culex_quinquefasciatus,CPIJ015162 culex_quinquefasciatus,CPIJ015368 culex_quinquefasciatus,CPIJ015369 culex_quinquefasciatus,CPIJ018205 culex_quinquefasciatus,CPIJ019821 culicoides_sonorensis,CSON004605 culicoides_sonorensis,CSON005566 culicoides_sonorensis,CSON008302 culicoides_sonorensis,CSON009338 culicoides_sonorensis,CSON009345 culicoides_sonorensis,CSON009526 culicoides_sonorensis,CSON012078 culicoides_sonorensis,CSON013394 culicoides_sonorensis,CSON015385 drosophila_melanogaster,FBgn0011834 drosophila_melanogaster,FBgn0030773 drosophila_melanogaster,FBgn0030774 drosophila_melanogaster,FBgn0030775 drosophila_melanogaster,FBgn0030776 drosophila_melanogaster,FBgn0030777 drosophila_melanogaster,FBgn0031141 drosophila_melanogaster,FBgn0038479 drosophila_melanogaster,FBgn0038481 drosophila_melanogaster,FBgn0038482 drosophila_melanogaster,FBgn0038484 drosophila_melanogaster,FBgn0038485 drosophila_melanogaster,FBgn0051265 drosophila_melanogaster,FBgn0051266 drosophila_melanogaster,FBgn0051267 drosophila_melanogaster,FBgn0051269 drosophila_melanogaster,FBgn0052523 glossina_morsitans,GMOY000672 glossina_morsitans,GMOY005309 glossina_morsitans,GMOY005310 glossina_morsitans,GMOY007343 glossina_morsitans,GMOY009756 glossina_morsitans,GMOY009757 glossina_morsitans,GMOY009758 lucilia_cuprina,FF38_02942 lucilia_cuprina,FF38_04538 lucilia_cuprina,FF38_04539 lucilia_cuprina,FF38_04541 lucilia_cuprina,FF38_04542 lucilia_cuprina,FF38_04543 lucilia_cuprina,FF38_04544 lucilia_cuprina,FF38_04545 lutzomyia_longipalpis,LLOJ005456 musca_domestica,MDOA000120 musca_domestica,MDOA000550 musca_domestica,MDOA001049 musca_domestica,MDOA001474 musca_domestica,MDOA001659 musca_domestica,MDOA002307 musca_domestica,MDOA002596 musca_domestica,MDOA003794 musca_domestica,MDOA004350 musca_domestica,MDOA004980 musca_domestica,MDOA005077 musca_domestica,MDOA005142 musca_domestica,MDOA006308 musca_domestica,MDOA006368 musca_domestica,MDOA006477 musca_domestica,MDOA006952 musca_domestica,MDOA007007 musca_domestica,MDOA007098 musca_domestica,MDOA009055 musca_domestica,MDOA010508 musca_domestica,MDOA011439 musca_domestica,MDOA011698 musca_domestica,MDOA011745 musca_domestica,MDOA013659 musca_domestica,MDOA014381 musca_domestica,MDOA015416 pediculus_humanus,PHUM440020 pediculus_humanus,PHUM610330 pediculus_humanus,PHUM610340 pediculus_humanus,PHUM610350 phlebotomus_papatasi,PPAI005023 phlebotomus_papatasi,PPAI007211 phlebotomus_papatasi,PPAI009146

		stomoxys_calcitrans,SCAU000050 stomoxys_calcitrans,SCAU000166 stomoxys_calcitrans,SCAU001401 stomoxys_calcitrans,SCAU001952 stomoxys_calcitrans,SCAU002905 stomoxys_calcitrans,SCAU003549 stomoxys_calcitrans,SCAU003706 stomoxys_calcitrans,SCAU004275 stomoxys_calcitrans,SCAU005222 stomoxys_calcitrans,SCAU005294 stomoxys_calcitrans,SCAU005527 stomoxys_calcitrans,SCAU005630 stomoxys_calcitrans,SCAU006301 stomoxys_calcitrans,SCAU007038 stomoxys_calcitrans,SCAU007204 stomoxys_calcitrans,SCAU008307 stomoxys_calcitrans,SCAU009133 stomoxys_calcitrans,SCAU009271 stomoxys_calcitrans,SCAU009533 stomoxys_calcitrans,SCAU011282 stomoxys_calcitrans,SCAU012368 stomoxys_calcitrans,SCAU013659 stomoxys_calcitrans,SCAU013728 stomoxys_calcitrans,SCAU013996 stomoxys_calcitrans,SCAU014102 stomoxys_calcitrans,SCAU014299 stomoxys_calcitrans,SCAU014798 stomoxys_calcitrans,SCAU015556 stomoxys_calcitrans,SCAU015598
FBgn0039494	Gram-positive Specific Serine protease	*aedes_aegypti,AAEL003219 aedes_aegypti,AAEL003243 aedes_aegypti,AAEL003253 aedes_aegypti,AAEL003279 aedes_aegypti,AAEL012030 aedes_aegypti,AAEL012775 aedes_aegypti,AAEL014724 aedes_aegypti,AAEL017003 aedes_aegypti,AAEL017555* anopheles_gambiae,AGAP004855 *belgica_antarctica,IU25_06082 belgica_antarctica,IU25_08080 culex_quinquefasciatus,CPIJ001059 culex_quinquefasciatus,CPIJ001060 culex_quinquefasciatus,CPIJ001061 culex_quinquefasciatus,CPIJ001062 culex_quinquefasciatus,CPIJ003623 culex_quinquefasciatus,CPIJ003631 culex_quinquefasciatus,CPIJ009208 culex_quinquefasciatus,CPIJ011900* culicoides_sonorensis,CSON003275 *drosophila_melanogaster,FBgn0033439 drosophila_melanogaster,FBgn0039494 drosophila_melanogaster,FBgn0039495 drosophila_melanogaster,FBgn0260474 glossina_morsitans,GMOY002008 glossina_morsitans,GMOY003273 glossina_morsitans,GMOY003357 glossina_morsitans,GMOY005559 lucilia_cuprina,FF38_09029 lucilia_cuprina,FF38_12589* lutzomyia_longipalpis,LLOJ005979 *musca_domestica,MDOA002242 musca_domestica,MDOA006413 musca_domestica,MDOA009320 musca_domestica,MDOA010716* pediculus_humanus,PHUM546590 *phlebotomus_papatasi,PPAI004260 phlebotomus_papatasi,PPAI006008 stomoxys_calcitrans,SCAU007556 stomoxys_calcitrans,SCAU007885 stomoxys_calcitrans,SCAU010045 stomoxys_calcitrans,SCAU012474*
FBgn0028436	ECSIT	acyrthosiphon_pisum,ACYPI009692 aedes_aegypti,AAEL005075 anopheles_gambiae,AGAP005295 culex_quinquefasciatus,CPIJ003637 drosophila_melanogaster,FBgn0028436 *glossina_morsitans,GMOY006941 glossina_morsitans,GMOY007478* lucilia_cuprina,FF38_07862 lutzomyia_longipalpis,LLOJ002734 musca_domestica,MDOA014241 pediculus_humanus,PHUM075600 phlebotomus_papatasi,PPAI008375 rhodnius_prolixus,RPRC014899 sarcoptes_scabiei,SSCA002855 stomoxys_calcitrans,SCAU007416 tetranychus_urticae,tetur03g05570
FBgn0026760	Tehao	*acyrthosiphon_pisum,ACYPI000177 acyrthosiphon_pisum,ACYPI002340 acyrthosiphon_pisum,ACYPI004287 acyrthosiphon_pisum,ACYPI060117 aedes_aegypti,AAEL000057 aedes_aegypti,AAEL003507 aedes_aegypti,AAEL007613 aedes_aegypti,AAEL007619 aedes_aegypti,AAEL014337 anopheles_gambiae,AGAP000999 anopheles_gambiae,AGAP001002 anopheles_gambiae,AGAP001004 anopheles_gambiae,AGAP004832 anopheles_gambiae,AGAP010636 anopheles_gambiae,AGAP010669 anopheles_gambiae,AGAP013027 belgica_antarctica,IU25_00799 belgica_antarctica,IU25_12757 belgica_antarctica,IU25_13021* culex_quinquefasciatus,CPIJ008497 *culicoides_sonorensis,CSON001282 culicoides_sonorensis,CSON001790*

		culicoides_sonorensis,CSON007335 culicoides_sonorensis,CSON011712 drosophila_melanogaster,FBgn0026760 drosophila_melanogaster,FBgn0262473 glossina_morsitans,GMOY011790 lucilia_cuprina,FF38_02759 musca_domestica,MDOA005484 pediculus_humanus,PHUM529420 rhodnius_prolixus,RPRC009262 stomoxys_calcitrans,SCAU010787 tetranychus_urticae,tetur06g04050
FBgn0262473	Toll	*acyrthosiphon_pisum,ACYPI000177 acyrthosiphon_pisum,ACYPI002340 acyrthosiphon_pisum,ACYPI004287 acyrthosiphon_pisum,ACYPI060117 aedes_aegypti,AAEL000057 aedes_aegypti,AAEL003507 aedes_aegypti,AAEL007613 aedes_aegypti,AAEL007619 aedes_aegypti,AAEL014337 anopheles_gambiae,AGAP000999 anopheles_gambiae,AGAP001002 anopheles_gambiae,AGAP001004 anopheles_gambiae,AGAP004832 anopheles_gambiae,AGAP010636 anopheles_gambiae,AGAP010669 anopheles_gambiae,AGAP013027 belgica_antarctica,IU25_00799 belgica_antarctica,IU25_12757 belgica_antarctica,IU25_13021* culex_quinquefasciatus,CPIJ008497 *culicoides_sonorensis,CSON001282 culicoides_sonorensis,CSON001790 culicoides_sonorensis,CSON007335 culicoides_sonorensis,CSON011712 drosophila_melanogaster,FBgn0026760 drosophila_melanogaster,FBgn0262473* glossina_morsitans,GMOY011790 lucilia_cuprina,FF38_02759 musca_domestica,MDOA005484 pediculus_humanus,PHUM529420 rhodnius_prolixus,RPRC009262 stomoxys_calcitrans,SCAU010787 tetranychus_urticae,tetur06g04050
FBgn0041205	kenny	aedes_aegypti,AAEL012510 anopheles_gambiae,AGAP005933 culex_quinquefasciatus,CPIJ006917 culicoides_sonorensis,CSON000146 drosophila_melanogaster,FBgn0041205 glossina_morsitans,GMOY010939 lucilia_cuprina,FF38_11364 lutzomyia_longipalpis,LLOJ005445 musca_domestica,MDOA003395 *stomoxys_calcitrans,SCAU014575 stomoxys_calcitrans,SCAU015154*
FBgn0002926	nudel	acyrthosiphon_pisum,ACYPI009492 aedes_aegypti,AAEL016971 anopheles_gambiae,AGAP007280 belgica_antarctica,IU25_07649 culex_quinquefasciatus,CPIJ002112 *culicoides_sonorensis,CSON004712 culicoides_sonorensis,CSON008920 culicoides_sonorensis,CSON014457* drosophila_melanogaster,FBgn0002926 glossina_morsitans,GMOY007795 lucilia_cuprina,FF38_11201 lutzomyia_longipalpis,LLOJ003338 musca_domestica,MDOA013497 pediculus_humanus,PHUM269170 rhodnius_prolixus,RPRC000049 sarcoptes_scabiei,SSCA001804 stomoxys_calcitrans,SCAU007954
FBgn0001990	weckle	aedes_aegypti,AAEL013154 anopheles_gambiae,AGAP009010 culex_quinquefasciatus,CPIJ010643 *culicoides_sonorensis,CSON001842 culicoides_sonorensis,CSON005192 drosophila_melanogaster,FBgn0001990 drosophila_melanogaster,FBgn0032763 drosophila_melanogaster,FBgn0032814 drosophila_melanogaster,FBgn0037794* glossina_morsitans,GMOY006026 *lucilia_cuprina,FF38_12650 lucilia_cuprina,FF38_12654 lutzomyia_longipalpis,LLOJ007464 lutzomyia_longipalpis,LLOJ007465 lutzomyia_longipalpis,LLOJ007466 lutzomyia_longipalpis,LLOJ007467 lutzomyia_longipalpis,LLOJ007470 lutzomyia_longipalpis,LLOJ007471 lutzomyia_longipalpis,LLOJ007472 musca_domestica,MDOA000077 musca_domestica,MDOA011589* pediculus_humanus,PHUM617670 *phlebotomus_papatasi,PPAI000051 phlebotomus_papatasi,PPAI000054 phlebotomus_papatasi,PPAI000059 phlebotomus_papatasi,PPAI000060 phlebotomus_papatasi,PPAI000456 stomoxys_calcitrans,SCAU003558 stomoxys_calcitrans,SCAU006483*
FBgn0261988	G protein-coupled receptor kinase 2	acyrthosiphon_pisum,ACYPI006008 aedes_aegypti,AAEL006110 anopheles_gambiae,AGAP004117 belgica_antarctica,IU25_05001 culicoides_sonorensis,CSON003998 drosophila_melanogaster,FBgn0261988

		glossina_morsitans,GMOY012151 lucilia_cuprina,FF38_11078 lutzomyia_longipalpis,LLOJ000872 musca_domestica,MDOA012220 pediculus_humanus,PHUM066650 phlebotomus_papatasi,PPAI001534 rhodnius_prolixus,RPRC007441 stomoxys_calcitrans,SCAU002473 tetranychus_urticae,tetur35g00320
FBgn0030051	Serine Protease Immune Response Integrator	*drosophila_melanogaster,FBgn0030051 drosophila_melanogaster,FBgn0038113 glossina_morsitans,GMOY000467 glossina_morsitans,GMOY001223 glossina_morsitans,GMOY004708 glossina_morsitans,GMOY008710 glossina_morsitans,GMOY008962 glossina_morsitans,GMOY008963 glossina_morsitans,GMOY008965 glossina_morsitans,GMOY008967 glossina_morsitans,GMOY010768 lucilia_cuprina,FF38_03663 lucilia_cuprina,FF38_03864 lucilia_cuprina,FF38_04969 musca_domestica,MDOA001390 musca_domestica,MDOA001460 musca_domestica,MDOA003746 musca_domestica,MDOA005035 musca_domestica,MDOA006445 musca_domestica,MDOA011101* phlebotomus_papatasi,PPAI009157 *stomoxys_calcitrans,SCAU004689 stomoxys_calcitrans,SCAU006186 stomoxys_calcitrans,SCAU009394*
FBgn0030926	persephone	*aedes_aegypti,AAEL004518 aedes_aegypti,AAEL004524 aedes_aegypti,AAEL004540 aedes_aegypti,AAEL012711 aedes_aegypti,AAEL012712 aedes_aegypti,AAEL012713 anopheles_gambiae,AGAP000315 anopheles_gambiae,AGAP000571 anopheles_gambiae,AGAP000572 anopheles_gambiae,AGAP000573 anopheles_gambiae,AGAP004719 belgica_antarctica,IU25_04025 belgica_antarctica,IU25_05842 belgica_antarctica,IU25_08416 belgica_antarctica,IU25_08947 belgica_antarctica,IU25_11200 culex_quinquefasciatus,CPIJ014649 culex_quinquefasciatus,CPIJ014650 culex_quinquefasciatus,CPIJ014651 culex_quinquefasciatus,CPIJ014652 culex_quinquefasciatus,CPIJ014653 culex_quinquefasciatus,CPIJ014654 culex_quinquefasciatus,CPIJ014655 culex_quinquefasciatus,CPIJ014656 culicoides_sonorensis,CSON008961 culicoides_sonorensis,CSON008962 culicoides_sonorensis,CSON009227 culicoides_sonorensis,CSON010323 culicoides_sonorensis,CSON011404 culicoides_sonorensis,CSON015270 culicoides_sonorensis,CSON015271 drosophila_melanogaster,FBgn0030925 drosophila_melanogaster,FBgn0030926* glossina_morsitans,GMOY005029 musca_domestica,MDOA002879 musca_domestica,MDOA015135 phlebotomus_papatasi,PPAI005881 stomoxys_calcitrans,SCAU006099
FBgn0003495	spatzle	*acyrthosiphon_pisum,ACYPI001858 acyrthosiphon_pisum,ACYPI004362 acyrthosiphon_pisum,ACYPI005491 acyrthosiphon_pisum,ACYPI41073 acyrthosiphon_pisum,ACYPI52992 aedes_aegypti,AAEL000499 aedes_aegypti,AAEL013433 aedes_aegypti,AAEL013434 aedes_aegypti,AAEL013435 culex_quinquefasciatus,CPIJ006792 culex_quinquefasciatus,CPIJ014270 culicoides_sonorensis,CSON002624 culicoides_sonorensis,CSON002625 culicoides_sonorensis,CSON005603 culicoides_sonorensis,CSON005604 culicoides_sonorensis,CSON011604* drosophila_melanogaster,FBgn0003495 glossina_morsitans,GMOY003060 lucilia_cuprina,FF38_00435 lutzomyia_longipalpis,LLOJ000602 musca_domestica,MDOA008361 pediculus_humanus,PHUM596260 phlebotomus_papatasi,PPAI002308 rhodnius_prolixus,RPRC002634 stomoxys_calcitrans,SCAU006182
FBgn0002930	necrotic	culicoides_sonorensis,CSON010106 *drosophila_melanogaster,FBgn0002930 drosophila_melanogaster,FBgn0024293 drosophila_melanogaster,FBgn0024294 drosophila_melanogaster,FBgn0028986 drosophila_melanogaster,FBgn0028987 drosophila_melanogaster,FBgn0028988 drosophila_melanogaster,FBgn0032178*

		drosophila_melanogaster,FBgn0033113 drosophila_melanogaster,FBgn0033115 drosophila_melanogaster,FBgn0033574 drosophila_melanogaster,FBgn0034195 drosophila_melanogaster,FBgn0044011 drosophila_melanogaster,FBgn0051902 drosophila_melanogaster,FBgn0053121 drosophila_melanogaster,FBgn0083141 glossina_morsitans,GMOY002444 glossina_morsitans,GMOY003656 glossina_morsitans,GMOY003657 lucilia_cuprina,FF38_01691 lucilia_cuprina,FF38_01698 lucilia_cuprina,FF38_02388 lucilia_cuprina,FF38_02390 lucilia_cuprina,FF38_14047 lutzomyia_longipalpis,LLOJ009184 *musca_domestica,MDOA003786 musca_domestica,MDOA004205 musca_domestica,MDOA004881 musca_domestica,MDOA008894 musca_domestica,MDOA008906 musca_domestica,MDOA013545 stomoxys_calcitrans,SCAU002599 stomoxys_calcitrans,SCAU009057 stomoxys_calcitrans,SCAU010576 stomoxys_calcitrans,SCAU014443 stomoxys_calcitrans,SCAU015518*
FBgn0003450	snake	acyrthosiphon_pisum,ACYPI001993 aedes_aegypti,AAEL007597 anopheles_gambiae,AGAP004318 culex_quinquefasciatus,CPIJ002753 *culicoides_sonorensis,CSON005854 culicoides_sonorensis,CSON011813* drosophila_melanogaster,FBgn0003450 lucilia_cuprina,FF38_03655 musca_domestica,MDOA013532 pediculus_humanus,PHUM003150 stomoxys_calcitrans,SCAU002882
FBgn0035379	spatzle 5	acyrthosiphon_pisum,ACYPI002943 aedes_aegypti,AAEL001929 anopheles_gambiae,AGAP007177 culex_quinquefasciatus,CPIJ009906 culicoides_sonorensis,CSON014426 drosophila_melanogaster,FBgn0035379 glossina_morsitans,GMOY007815 lucilia_cuprina,FF38_12212 musca_domestica,MDOA012839 pediculus_humanus,PHUM601860 sarcoptes_scabiei,SSCA004651 stomoxys_calcitrans,SCAU015616 *tetranychus_urticae,tetur04g00590 tetranychus_urticae,tetur20g01990*
FBgn0030310	Peptidoglycan recognition protein SA	aedes_aegypti,AAEL009474 anopheles_gambiae,AGAP000536 belgica_antarctica,IU25_03404 culex_quinquefasciatus,CPIJ007162 drosophila_melanogaster,FBgn0030310 glossina_morsitans,GMOY009549 lucilia_cuprina,FF38_10195 *lutzomyia_longipalpis,LLOJ002444 lutzomyia_longipalpis,LLOJ004539 lutzomyia_longipalpis,LLOJ005642* musca_domestica,MDOA014683 pediculus_humanus,PHUM581030 *phlebotomus_papatasi,PPAI010203 phlebotomus_papatasi,PPAI010204* sarcoptes_scabiei,SSCA010190 *stomoxys_calcitrans,SCAU001267 stomoxys_calcitrans,SCAU015071 stomoxys_calcitrans,SCAU015258*
FBgn0265464	TNF-receptor-associated factor 6	*acyrthosiphon_pisum,ACYPI065115 acyrthosiphon_pisum,ACYPI072124 acyrthosiphon_pisum,ACYPI41089* anopheles_gambiae,AGAP003004 belgica_antarctica,IU25_01168 drosophila_melanogaster,FBgn0265464 glossina_morsitans,GMOY009472 lucilia_cuprina,FF38_06337 lutzomyia_longipalpis,LLOJ003524 musca_domestica,MDOA002419 pediculus_humanus,PHUM129280 phlebotomus_papatasi,PPAI010681 rhodnius_prolixus,RPRC006736 stomoxys_calcitrans,SCAU005776
FBgn0003882	tube	drosophila_melanogaster,FBgn0003882 glossina_morsitans,GMOY007350 lutzomyia_longipalpis,LLOJ005198 musca_domestica,MDOA011835 phlebotomus_papatasi,PPAI003151 stomoxys_calcitrans,SCAU003206
FBgn0000808	gastrulation-defective	aedes_aegypti,AAEL006123 belgica_antarctica,IU25_06559 culicoides_sonorensis,CSON009297 drosophila_melanogaster,FBgn0000808 lucilia_cuprina,FF38_01028 l*utzomyia_longipalpis,LLOJ001114 lutzomyia_longipalpis,LLOJ001115 lutzomyia_longipalpis,LLOJ001119 lutzomyia_longipalpis,LLOJ001120 lutzomyia_longipalpis,LLOJ002161 lutzomyia_longipalpis,LLOJ004336 lutzomyia_longipalpis,LLOJ006990* musca_domestica,MDOA011269 pediculus_humanus,PHUM609560

		phlebotomus_papatasi,PPAI003030 phlebotomus_papatasi,PPAI004659 phlebotomus_papatasi,PPAI004660 phlebotomus_papatasi,PPAI004661 phlebotomus_papatasi,PPAI004666 phlebotomus_papatasi,PPAI004667 phlebotomus_papatasi,PPAI005261 rhodnius_prolixus,RPRC001191 stomoxys_calcitrans,SCAU000651
FBgn0024222	immune response deficient 5	*aedes_aegypti,AAEL003245 aedes_aegypti,AAEL010548 aedes_aegypti,AAEL010553* anopheles_gambiae,AGAP009166 culex_quinquefasciatus,CPIJ015672 culicoides_sonorensis,CSON001226 drosophila_melanogaster,FBgn0024222 glossina_morsitans,GMOY007052 lucilia_cuprina,FF38_05363 lutzomyia_longipalpis,LLOJ000102 musca_domestica,MDOA001165 pediculus_humanus,PHUM605130 phlebotomus_papatasi,PPAI008439 sarcoptes_scabiei,SSCA010067 stomoxys_calcitrans,SCAU008653

b)

FlyBase gene ID	FlyBase Gene name	Compara family
FBgn0051410		aedes_aegypti,AAEL009553 aedes_aegypti,AAEL009555 aedes_aegypti,AAEL009556 aedes_aegypti,AAEL009557 aedes_aegypti,AAEL009760 aedes_aegypti,AAEL009953 aedes_aegypti,AAEL009954 aedes_aegypti,AAEL009956 aedes_aegypti,AAEL015135 aedes_aegypti,AAEL015136 aedes_aegypti,AAEL015138 aedes_aegypti,AAEL015139 aedes_aegypti,AAEL015140 aedes_aegypti,AAEL015516 anopheles_gambiae,AGAP002804 anopheles_gambiae,AGAP002847 anopheles_gambiae,AGAP002848 anopheles_gambiae,AGAP002849 anopheles_gambiae,AGAP002850 anopheles_gambiae,AGAP002851 anopheles_gambiae,AGAP007197 anopheles_gambiae,AGAP013388 belgica_antarctica,IU25_09972 culex_quinquefasciatus,CPIJ000782 culex_quinquefasciatus,CPIJ002737 culex_quinquefasciatus,CPIJ002745 culex_quinquefasciatus,CPIJ002746 culex_quinquefasciatus,CPIJ002747 culex_quinquefasciatus,CPIJ012180 culex_quinquefasciatus,CPIJ013180 culex_quinquefasciatus,CPIJ013182 culex_quinquefasciatus,CPIJ018326 culicoides_sonorensis,CSON000565 culicoides_sonorensis,CSON001914 culicoides_sonorensis,CSON003895 culicoides_sonorensis,CSON003925 culicoides_sonorensis,CSON004032 culicoides_sonorensis,CSON007608 culicoides_sonorensis,CSON008026 culicoides_sonorensis,CSON008571 culicoides_sonorensis,CSON013231 drosophila_melanogaster,FBgn0037782 drosophila_melanogaster,FBgn0037783 drosophila_melanogaster,FBgn0051410 glossina_morsitans,GMOY007339 lucilia_cuprina,FF38_03640 lucilia_cuprina,FF38_03645 lutzomyia_longipalpis,LLOJ000496 lutzomyia_longipalpis,LLOJ001852 lutzomyia_longipalpis,LLOJ001853 lutzomyia_longipalpis,LLOJ001854 lutzomyia_longipalpis,LLOJ001859 lutzomyia_longipalpis,LLOJ001860 musca_domestica,MDOA001478 musca_domestica,MDOA002245 musca_domestica,MDOA002326 musca_domestica,MDOA004776 musca_domestica,MDOA009576 musca_domestica,MDOA013713 musca_domestica,MDOA014505 musca_domestica,MDOA014594 phlebotomus_papatasi,PPAI004539 phlebotomus_papatasi,PPAI007804 phlebotomus_papatasi,PPAI010333 rhodnius_prolixus,RPRC011677 stomoxys_calcitrans,SCAU001972 stomoxys_calcitrans,SCAU011642 stomoxys_calcitrans,SCAU013599 stomoxys_calcitrans,SCAU016246

FBgn0038928		aedes_aegypti,AAEL001932 anopheles_gambiae,AGAP007173 culex_quinquefasciatus,CPIJ009900 drosophila_melanogaster,FBgn0038928 glossina_morsitans,GMOY007803 lucilia_cuprina,FF38_05986 musca_domestica,MDOA013513 stomoxys_calcitrans,SCAU000983
FBgn0086358		acyrthosiphon_pisum,ACYPI002796 belgica_antarctica,IU25_09152 culex_quinquefasciatus,CPIJ000820 culicoides_sonorensis,CSON004331 culicoides_sonorensis,CSON012364 drosophila_melanogaster,FBgn0086358 lucilia_cuprina,FF38_02190 musca_domestica,MDOA008134 pediculus_humanus,PHUM268290 stomoxys_calcitrans,SCAU015042
FBgn0031381		aedes_aegypti,AAEL006854 anopheles_gambiae,AGAP028108 anopheles_gambiae,AGAP028157 culex_quinquefasciatus,CPIJ004652 culicoides_sonorensis,CSON004270 culicoides_sonorensis,CSON004915 drosophila_melanogaster,FBgn0031381 glossina_morsitans,GMOY008129 lucilia_cuprina,FF38_00548 lutzomyia_longipalpis,LLOJ004428 musca_domestica,MDOA000311 stomoxys_calcitrans,SCAU007166
FBgn0035976		aedes_aegypti,AAEL003037 aedes_aegypti,AAEL014640 anopheles_gambiae,AGAP005203 culex_quinquefasciatus,CPIJ006559 culex_quinquefasciatus,CPIJ006560 culex_quinquefasciatus,CPIJ006561 culicoides_sonorensis,CSON003218 culicoides_sonorensis,CSON008584 drosophila_melanogaster,FBgn0035806 drosophila_melanogaster,FBgn0035976 drosophila_melanogaster,FBgn0035977 glossina_morsitans,GMOY006094 lucilia_cuprina,FF38_06093 lucilia_cuprina,FF38_06096 lutzomyia_longipalpis,LLOJ001985 musca_domestica,MDOA004742 musca_domestica,MDOA013494 musca_domestica,MDOA014677 phlebotomus_papatasi,PPAI007066 phlebotomus_papatasi,PPAI007689 stomoxys_calcitrans,SCAU004266 stomoxys_calcitrans,SCAU004566
FBgn0035977		aedes_aegypti,AAEL003037 aedes_aegypti,AAEL014640 anopheles_gambiae,AGAP005203 culex_quinquefasciatus,CPIJ006559 culex_quinquefasciatus,CPIJ006560 culex_quinquefasciatus,CPIJ006561 culicoides_sonorensis,CSON003218 culicoides_sonorensis,CSON008584 drosophila_melanogaster,FBgn0035806 drosophila_melanogaster,FBgn0035976 drosophila_melanogaster,FBgn0035977 glossina_morsitans,GMOY006094 lucilia_cuprina,FF38_06093 lucilia_cuprina,FF38_06096 lutzomyia_longipalpis,LLOJ001985 musca_domestica,MDOA004742 musca_domestica,MDOA013494 musca_domestica,MDOA014677 phlebotomus_papatasi,PPAI007066 phlebotomus_papatasi,PPAI007689 stomoxys_calcitrans,SCAU004266 stomoxys_calcitrans,SCAU004566
FBgn0015247		acyrthosiphon_pisum,ACYPI000445 acyrthosiphon_pisum,ACYPI004833 acyrthosiphon_pisum,ACYPI009069 acyrthosiphon_pisum,ACYPI009246 acyrthosiphon_pisum,ACYPI060207 acyrthosiphon_pisum,ACYPI060486 acyrthosiphon_pisum,ACYPI060860 acyrthosiphon_pisum,ACYPI061109 acyrthosiphon_pisum,ACYPI062047 acyrthosiphon_pisum,ACYPI063520 acyrthosiphon_pisum,ACYPI063937 acyrthosiphon_pisum,ACYPI064349 acyrthosiphon_pisum,ACYPI064544 acyrthosiphon_pisum,ACYPI064683 acyrthosiphon_pisum,ACYPI067471 acyrthosiphon_pisum,ACYPI068836 acyrthosiphon_pisum,ACYPI069969 acyrthosiphon_pisum,ACYPI069986 acyrthosiphon_pisum,ACYPI070061 acyrthosiphon_pisum,ACYPI071076 acyrthosiphon_pisum,ACYPI071478 acyrthosiphon_pisum,ACYPI071618 acyrthosiphon_pisum,ACYPI072406 acyrthosiphon_pisum,ACYPI073087 acyrthosiphon_pisum,ACYPI080425 acyrthosiphon_pisum,ACYPI080821

		acyrthosiphon_pisum,ACYPI081275 acyrthosiphon_pisum,ACYPI081358 acyrthosiphon_pisum,ACYPI081487 acyrthosiphon_pisum,ACYPI081511 acyrthosiphon_pisum,ACYPI082343 acyrthosiphon_pisum,ACYPI082506 acyrthosiphon_pisum,ACYPI082711 acyrthosiphon_pisum,ACYPI083692 acyrthosiphon_pisum,ACYPI083704 acyrthosiphon_pisum,ACYPI083959 acyrthosiphon_pisum,ACYPI084397 acyrthosiphon_pisum,ACYPI085320 acyrthosiphon_pisum,ACYPI086150 acyrthosiphon_pisum,ACYPI086274 acyrthosiphon_pisum,ACYPI086879 acyrthosiphon_pisum,ACYPI087791 acyrthosiphon_pisum,ACYPI088105 acyrthosiphon_pisum,ACYPI089058 acyrthosiphon_pisum,ACYPI089458 acyrthosiphon_pisum,ACYPI33497 acyrthosiphon_pisum,ACYPI40719 acyrthosiphon_pisum,ACYPI41749 acyrthosiphon_pisum,ACYPI47179 acyrthosiphon_pisum,ACYPI50284 aedes_aegypti,AAEL006633 aedes_aegypti,AAEL009074 anopheles_gambiae,AGAP007291 anopheles_gambiae,AGAP007292 anopheles_gambiae,AGAP007293 anopheles_gambiae,AGAP007294 anopheles_gambiae,AGAP011326 anopheles_gambiae,AGAP012677 anopheles_gambiae,AGAP013034 belgica_antarctica,IU25_02332 belgica_antarctica,IU25_06012 belgica_antarctica,IU25_06013 culex_quinquefasciatus,CPIJ002102 culex_quinquefasciatus,CPIJ019231 culicoides_sonorensis,CSON007331 culicoides_sonorensis,CSON010539 drosophila_melanogaster,FBgn0015247 drosophila_melanogaster,FBgn0260635 glossina_morsitans,GMOY002322 glossina_morsitans,GMOY003276 lucilia_cuprina,FF38_06733 lucilia_cuprina,FF38_12870 lutzomyia_longipalpis,LLOJ010489 musca_domestica,MDOA000675 musca_domestica,MDOA011063 pediculus_humanus,PHUM080100 pediculus_humanus,PHUM253740 phlebotomus_papatasi,PPAI002073 phlebotomus_papatasi,PPAI002074 phlebotomus_papatasi,PPAI003908 phlebotomus_papatasi,PPAI003909 phlebotomus_papatasi,PPAI005737 rhodnius_prolixus,RPRC001809 rhodnius_prolixus,RPRC007068 sarcoptes_scabiei,SSCA002366 stomoxys_calcitrans,SCAU003922 stomoxys_calcitrans,SCAU005544 tetranychus_urticae,tetur01g10290 tetranychus_urticae,tetur01g12100 tetranychus_urticae,tetur02g10150 tetranychus_urticae,tetur03g03360 tetranychus_urticae,tetur03g09850 tetranychus_urticae,tetur05g00590 tetranychus_urticae,tetur06g00960 tetranychus_urticae,tetur06g03410 tetranychus_urticae,tetur07g02900 tetranychus_urticae,tetur07g07740 tetranychus_urticae,tetur08g05960 tetranychus_urticae,tetur12g01630 tetranychus_urticae,tetur19g00030 tetranychus_urticae,tetur212g00020 tetranychus_urticae,tetur32g01500 tetranychus_urticae,tetur39g00770 tetranychus_urticae,tetur45g00100 tetranychus_urticae,tetur45g00130 tetranychus_urticae,tetur58g00080 tetranychus_urticae,tetur59g00020
FBgn0013983		aedes_aegypti,AAEL010083 anopheles_gambiae,AGAP004959 culex_quinquefasciatus,CPIJ010891 drosophila_melanogaster,FBgn0013983 glossina_morsitans,GMOY013006 lutzomyia_longipalpis,LLOJ010482 musca_domestica,MDOA006138 stomoxys_calcitrans,SCAU005739
FBgn0020381		aedes_aegypti,AAEL014148 aedes_aegypti,AAEL014658 anopheles_gambiae,AGAP011693 belgica_antarctica,IU25_03714 culex_quinquefasciatus,CPIJ008065 culex_quinquefasciatus,CPIJ008066 culex_quinquefasciatus,CPIJ008093 culex_quinquefasciatus,CPIJ016551 culex_quinquefasciatus,CPIJ017008 culicoides_sonorensis,CSON010543 culicoides_sonorensis,CSON010544 culicoides_sonorensis,CSON012690 drosophila_melanogaster,FBgn0020381 glossina_morsitans,GMOY007083

		lucilia_cuprina,FF38_04277 lutzomyia_longipalpis,LLOJ010485 musca_domestica,MDOA000407 pediculus_humanus,PHUM574530 phlebotomus_papatasi,PPAI000697 stomoxys_calcitrans,SCAU008187
FBgn0030695		aedes_aegypti,AAEL013112 culex_quinquefasciatus,CPIJ000672 drosophila_melanogaster,FBgn0030695 lucilia_cuprina,FF38_05391 lutzomyia_longipalpis,LLOJ003999 musca_domestica,MDOA013433 stomoxys_calcitrans,SCAU000990 stomoxys_calcitrans,SCAU005689
FBgn0014018		aedes_aegypti,AAEL007624 anopheles_gambiae,AGAP006747 belgica_antarctica,IU25_06473 culex_quinquefasciatus,CPIJ012236 culicoides_sonorensis,CSON014412 drosophila_melanogaster,FBgn0014018 glossina_morsitans,GMOY013090 lucilia_cuprina,FF38_04187 lutzomyia_longipalpis,LLOJ002097 lutzomyia_longipalpis,LLOJ002098 musca_domestica,MDOA004895 pediculus_humanus,PHUM424590 phlebotomus_papatasi,PPAI012820 rhodnius_prolixus,RPRC017358 sarcoptes_scabiei,SSCA006430 stomoxys_calcitrans,SCAU011881 tetranychus_urticae,tetur07g00650

c)

FlyBase gene ID	FlyBase Gene name	Compara family
FBgn0004107		aedes_aegypti,AAEL012339 aedes_aegypti,AAEL013329 anopheles_gambiae,AGAP004579 belgica_antarctica,IU25_01953 culex_quinquefasciatus,CPIJ011080 culicoides_sonorensis,CSON006415 drosophila_melanogaster,FBgn0004107 glossina_morsitans,GMOY008507 lucilia_cuprina,FF38_03798 lutzomyia_longipalpis,LLOJ009248 musca_domestica,MDOA004429 pediculus_humanus,PHUM190130 phlebotomus_papatasi,PPAI002174 phlebotomus_papatasi,PPAI003464 sarcoptes_scabiei,SSCA004145 stomoxys_calcitrans,SCAU014408 tetranychus_urticae,tetur06g06480
FBgn0016917		acyrthosiphon_pisum,ACYPI002351 acyrthosiphon_pisum,ACYPI005642 acyrthosiphon_pisum,ACYPI44613 aedes_aegypti,AAEL009692 anopheles_gambiae,AGAP000099 anopheles_gambiae,AGAP010423 belgica_antarctica,IU25_07727 belgica_antarctica,IU25_09609 culex_quinquefasciatus,CPIJ016469 culex_quinquefasciatus,CPIJ016470 culex_quinquefasciatus,CPIJ016471 culicoides_sonorensis,CSON006720 culicoides_sonorensis,CSON012361 culicoides_sonorensis,CSON013044 drosophila_melanogaster,FBgn0016917 glossina_morsitans,GMOY003392 glossina_morsitans,GMOY003393 glossina_morsitans,GMOY003394 glossina_morsitans,GMOY008510 lucilia_cuprina,FF38_03653 lutzomyia_longipalpis,LLOJ002980 lutzomyia_longipalpis,LLOJ007427 lutzomyia_longipalpis,LLOJ007428 lutzomyia_longipalpis,LLOJ007429 musca_domestica,MDOA006256 musca_domestica,MDOA009503 pediculus_humanus,PHUM335200 phlebotomus_papatasi,PPAI002926 phlebotomus_papatasi,PPAI003063 phlebotomus_papatasi,PPAI006937 rhodnius_prolixus,RPRC006502 sarcoptes_scabiei,SSCA008207 sarcoptes_scabiei,SSCA010326 stomoxys_calcitrans,SCAU011387 tetranychus_urticae,tetur17g02010 tetranychus_urticae,tetur36g00600
FBgn0010315		acyrthosiphon_pisum,ACYPI008338 belgica_antarctica,IU25_04528 culex_quinquefasciatus,CPIJ014520 drosophila_melanogaster,FBgn0010315 glossina_morsitans,GMOY007101 lucilia_cuprina,FF38_11234 lutzomyia_longipalpis,LLOJ000889 musca_domestica,MDOA006089 pediculus_humanus,PHUM503450 phlebotomus_papatasi,PPAI010406 rhodnius_prolixus,RPRC004503 stomoxys_calcitrans,SCAU016207
FBgn0016131		acyrthosiphon_pisum,ACYPI005353 aedes_aegypti,AAEL001407 anopheles_gambiae,AGAP005817 belgica_antarctica,IU25_10627

		culex_quinquefasciatus,CPIJ015895 drosophila_melanogaster,FBgn0016131 glossina_morsitans,GMOY005226 lucilia_cuprina,FF38_02007 lutzomyia_longipalpis,LLOJ003732 musca_domestica,MDOA009412 pediculus_humanus,PHUM065530 rhodnius_prolixus,RPRC001940 stomoxys_calcitrans,SCAU009670 tetranychus_urticae,tetur14g03700
FBgn0004956		culicoides_sonorensis,CSON004570 drosophila_melanogaster,FBgn0004956 drosophila_melanogaster,FBgn0030904 drosophila_melanogaster,FBgn0053542 glossina_morsitans,GMOY003974 glossina_morsitans,GMOY003976 glossina_morsitans,GMOY004620 lucilia_cuprina,FF38_03109 lucilia_cuprina,FF38_03111 lucilia_cuprina,FF38_03112 musca_domestica,MDOA004218 musca_domestica,MDOA004259 musca_domestica,MDOA012067 stomoxys_calcitrans,SCAU012598 stomoxys_calcitrans,SCAU012996 stomoxys_calcitrans,SCAU016514
FBgn0053542		culicoides_sonorensis,CSON004570 drosophila_melanogaster,FBgn0004956 drosophila_melanogaster,FBgn0030904 drosophila_melanogaster,FBgn0053542 glossina_morsitans,GMOY003974 glossina_morsitans,GMOY003976 glossina_morsitans,GMOY004620 lucilia_cuprina,FF38_03109 lucilia_cuprina,FF38_03111 lucilia_cuprina,FF38_03112 musca_domestica,MDOA004218 musca_domestica,MDOA004259 musca_domestica,MDOA012067 stomoxys_calcitrans,SCAU012598 stomoxys_calcitrans,SCAU012996 stomoxys_calcitrans,SCAU016514
FBgn0043903		acyrthosiphon_pisum,ACYPI082699 acyrthosiphon_pisum,ACYPI21995 acyrthosiphon_pisum,ACYPI21996 acyrthosiphon_pisum,ACYPI24864 acyrthosiphon_pisum,ACYPI40957 acyrthosiphon_pisum,ACYPI40958 acyrthosiphon_pisum,ACYPI56790 aedes_aegypti,AAEL000309 aedes_aegypti,AAEL012471 anopheles_gambiae,AGAP010083 belgica_antarctica,IU25_05199 belgica_antarctica,IU25_05200 culex_quinquefasciatus,CPIJ000838 culex_quinquefasciatus,CPIJ000839 culex_quinquefasciatus,CPIJ004128 culex_quinquefasciatus,CPIJ017416 culicoides_sonorensis,CSON004107 culicoides_sonorensis,CSON004944 culicoides_sonorensis,CSON004949 drosophila_melanogaster,FBgn0043903 glossina_morsitans,GMOY005024 glossina_morsitans,GMOY006582 glossina_morsitans,GMOY007646 lucilia_cuprina,FF38_02338 lucilia_cuprina,FF38_02341 lucilia_cuprina,FF38_03766 lucilia_cuprina,FF38_05364 lucilia_cuprina,FF38_13801 lutzomyia_longipalpis,LLOJ002449 musca_domestica,MDOA005916 musca_domestica,MDOA013858 musca_domestica,MDOA014974 pediculus_humanus,PHUM374950 phlebotomus_papatasi,PPAI001065 rhodnius_prolixus,RPRC003343 stomoxys_calcitrans,SCAU002518 stomoxys_calcitrans,SCAU008280 stomoxys_calcitrans,SCAU011284
FBgn0027363		acyrthosiphon_pisum,ACYPI008100 aedes_aegypti,AAEL002240 anopheles_gambiae,AGAP008494 culex_quinquefasciatus,CPIJ003516 culicoides_sonorensis,CSON006278 drosophila_melanogaster,FBgn0027363 glossina_morsitans,GMOY002316 lucilia_cuprina,FF38_14423 lutzomyia_longipalpis,LLOJ006372 musca_domestica,MDOA005586 pediculus_humanus,PHUM584200 phlebotomus_papatasi,PPAI003122 rhodnius_prolixus,RPRC015396 sarcoptes_scabiei,SSCA007045 stomoxys_calcitrans,SCAU003350 tetranychus_urticae,tetur05g08180
FBgn0041184		acyrthosiphon_pisum,ACYPI003795 aedes_aegypti,AAEL000393 anopheles_gambiae,AGAP011042 belgica_antarctica,IU25_08607

		culex_quinquefasciatus,CPIJ003380 culicoides_sonorensis,CSON012903 drosophila_melanogaster,FBgn0041184 glossina_morsitans,GMOY007838 lutzomyia_longipalpis,LLOJ009450 musca_domestica,MDOA008192 pediculus_humanus,PHUM164470 rhodnius_prolixus,RPRC002037 sarcoptes_scabiei,SSCA002433 stomoxys_calcitrans,SCAU009634
FBgn0010382		acyrthosiphon_pisum,ACYPI000629 aedes_aegypti,AAEL009057 anopheles_gambiae,AGAP008191 belgica_antarctica,IU25_09316 culex_quinquefasciatus,CPIJ000483 culicoides_sonorensis,CSON012774 drosophila_melanogaster,FBgn0010382 glossina_morsitans,GMOY011264 lucilia_cuprina,FF38_06868 lutzomyia_longipalpis,LLOJ003487 musca_domestica,MDOA004042 pediculus_humanus,PHUM377500 phlebotomus_papatasi,PPAI003313 rhodnius_prolixus,RPRC007105 sarcoptes_scabiei,SSCA010613 stomoxys_calcitrans,SCAU003616 tetranychus_urticae,tetur21g02080
FBgn0262114		acyrthosiphon_pisum,ACYPI009438 aedes_aegypti,AAEL008645 anopheles_gambiae,AGAP005063 belgica_antarctica,IU25_00055 belgica_antarctica,IU25_00349 culex_quinquefasciatus,CPIJ000068 drosophila_melanogaster,FBgn0262114 glossina_morsitans,GMOY009172 lucilia_cuprina,FF38_03213 lutzomyia_longipalpis,LLOJ008534 musca_domestica,MDOA004470 pediculus_humanus,PHUM378980 phlebotomus_papatasi,PPAI000722 rhodnius_prolixus,RPRC000877 rhodnius_prolixus,RPRC005695 rhodnius_prolixus,RPRC009324 sarcoptes_scabiei,SSCA005554 stomoxys_calcitrans,SCAU006114 tetranychus_urticae,tetur13g03850
FBgn0004864		acyrthosiphon_pisum,ACYPI066985 aedes_aegypti,AAEL012553 anopheles_gambiae,AGAP008354 culicoides_sonorensis,CSON002696 culicoides_sonorensis,CSON003569 culicoides_sonorensis,CSON007500 culicoides_sonorensis,CSON014132 drosophila_melanogaster,FBgn0004864 glossina_morsitans,GMOY005985 lucilia_cuprina,FF38_05372 lutzomyia_longipalpis,LLOJ007872 musca_domestica,MDOA010997 pediculus_humanus,PHUM202560 phlebotomus_papatasi,PPAI004680 rhodnius_prolixus,RPRC012987 sarcoptes_scabiei,SSCA002039 sarcoptes_scabiei,SSCA004374 sarcoptes_scabiei,SSCA007120 stomoxys_calcitrans,SCAU016080 tetranychus_urticae,tetur02g05790
FBgn0030904		culicoides_sonorensis,CSON004570 drosophila_melanogaster,FBgn0004956 drosophila_melanogaster,FBgn0030904 drosophila_melanogaster,FBgn0053542 glossina_morsitans,GMOY003974 glossina_morsitans,GMOY003976 glossina_morsitans,GMOY004620 lucilia_cuprina,FF38_03109 lucilia_cuprina,FF38_03111 lucilia_cuprina,FF38_03112 musca_domestica,MDOA004218 musca_domestica,MDOA004259 musca_domestica,MDOA012067 stomoxys_calcitrans,SCAU012598 stomoxys_calcitrans,SCAU012996 stomoxys_calcitrans,SCAU016514

Table S9. Immune related genes identified in *C. sonorensis* using BLASTp top hits with the gene copies from *A. aegypti* and *Cx. quinquefasciatus* available in the ImmunoDB database (http://cegg.unige.ch/Insecta/immunodb) as query. a) Anti-Microbial Peptides; b) Toll receptors; c) Toll path; d) jak/Stat path; e). Imd path. * - Top BLASTp hits different found when *A. aegypti* and *Cx. quinquefasciatus* were used as query.

a)

A. aegypti ID	*Cx. quinquefasciatus* ID	Gene name	Gene subfamily	*C. sonorensis*
AMP14	n.a.	Att	Attacin	CSON009409
AMP5		CECD	Cecropin	CSON002659
AMP6		CECE	Cecropin	CSON002659
AMP7		CECN	Cecropin	CSON002659
AMP8		CECF	Cecropin	CSON002659
AMP9		CECA	Cecropin	CSON002659
AMP10		CECI	Cecropin	CSON002659
AMP11		CECJ	Cecropin	CSON002659
AMP12		CECB	Cecropin	CSON002659
AMP13		CECG	Cecropin	CSON002659
AMP15		CECH	Cecropin	CSON002659
	AMP2	CECA1	Cecropin	CSON002659
	AMP3	CECA2	Cecropin	CSON002659
	AMP4	CECB1	Cecropin	CSON002659
	AMP5	CECB2	Cecropin	CSON002659
AMP1		DEFD	Defensin	CSON013028
AMP2		DEFC	Defensin	CSON013028
AMP3	AMP6	DEFA	Defensin	CSON013028
AMP4		DEFE	Defensin	CSON013028
AMP16	AMP1	DPT	Diptericin	CSON003561
AMP18	AMP7	GAM	Gambicin	No hit
AMP17		GRRP	Holotricin	No hit

b)

A. aegypti ID	*Cx. quinquefasciatus* ID	Gene name	Gene subfamily	*C. sonorensis*
TOLL5	TOLL10	TOLL10	Toll	CSON003960
TOLL4	TOLL11	TOLL11	Toll	CSON003960
TOLL3	TOLL1	TOLL1A	Toll	CSON007335 / CSON011712*
TOLL8	TOLL2	TOLL1B	Toll	CSON007335
TOLL6		TOLL4	Toll	CSON011712
TOLL7		TOLL5A	Toll	CSON007335
TOLL9		TOLL5B	Toll	No hits
TOLL12	TOLL6	TOLL6	Toll	CSON000010
TOLL10	TOLL7	TOLL7	Toll	CSON003737
TOLL1	TOLL8	TOLL8	Toll	CSON008073
TOLL11	TOLL3	TOLL9A	Toll	CSON008073 / CSON002070*
TOLL2	TOLL4	TOLL9B	Toll	CSON002070 / CSON006589*

	TOLL5		Toll	No hit
	TOLL9		Toll	No hit

c)

***A. aegypti* ID**	***Cx. quinquefasciatus* ID**	**Gene name**	**Gene subfamily**	***C. sonorensis***
TOLLPATH4	TOLLPATH5	CACT	CACT	CSON002111
TOLLPATH3	TOLLPATH4	MYD88	MYD88	CSON001312
	TOLLPATH3		MYD88	No hit
	TOLLPATH6		PELLE	No hit
TOLLPATH2	TOLLPATH2	PELLE	PELLE	CSON012655
TOLLPATH5	TOLLPATH7	TRAF6	TRAF6	CSON008794
TOLLPATH1	TOLLPATH1	TUBE	TUBE	CSON013585

d)

***A. aegypti* ID**	***Cx. quinquefasciatus* ID**	**Gene name**	**Gene subfamily**	***C. sonorensis***
JAKSTAT1	JAKSTAT1	DOME	DOME	CSON004944
JAKSTAT2	JAKSTAT2	HOP	HOP	CSON002696
JAKSTAT3	JAKSTAT3	STAT1A	STAT	CSON013044
	JAKSTAT4	STAT1B	STAT	CSON012361
	JAKSTAT5	STAT1C	STAT	CSON013044

e)

***A. aegypti* ID**	***Cx. quinquefasciatus* ID**	**Gene name**	**Gene subfamily**	***C. sonorensis***
IMDPATH2	IMDPATH2	TAK1	TAK1	CSON008729
IMDPATH3	IMDPATH3	IKK2	IKKg	CSON000146
IMDPATH4	IMDPATH4	IKK1A	IKKb	CSON001226
IMDPATH7	IMDPATH7	PIRK	PIRK	CSON010320
IMDPATH10	IMDPATH6	TAB2	TAB2	CSON004331

Table S10. Details of the BLASTp results using *A. aegypti* (a) and *Cx. quinquefasciatus* (b) as query to identify the *C. sonorensis* genes of the immune pathways.

a)

Query	Target	evalue	% identity	qstart	sstart	qend	send
Aaeg:TOLL12	CSON000010-1_12385:20136_culicoides_sonorensis_1004:1:76358	0	64.73	37	29	1355	1382
Aaeg:IMDPATH3	CSON000146-1_188504:193471_culicoides_sonorensis_101:1:286939	2.00E-72	33.47	3	14	489	470
Aaeg:IMDPATH4	CSON001226-1_213213:218118_culicoides_sonorensis_118:1:280338	0	45.48	8	4	790	744
Aaeg:IMDPATH6	CSON001226-1_213213:218118_culicoides_sonorensis_118:1:280338	6.00E-132	52.04	7	4	351	370
Aaeg:TOLLPATH3	CSON001312-1_215650:225787_culicoides_sonorensis_11:1:572898	1.00E-51	36.01	17	10	315	311
Aaeg:TOLL2	CSON002070-1_13325:17028_culicoides_sonorensis_1342:1:30232	3.00E-148	38.79	20	202	727	887
Aaeg:TOLLPATH4	CSON002111-1_133327:143976_culicoides_sonorensis_134:1:259523	1.00E-64	39.73	26	22	362	362
Aaeg:AMP7	CSON002659-1_3775:5747_culicoides_sonorensis_1449:1:12322	1.00E-19	64.52	1	1	62	58
Aaeg:AMP13	CSON002659-1_3775:5747_culicoides_sonorensis_1449:1:12322	4.00E-15	59.32	1	1	59	59
Aaeg:AMP9	CSON002659-1_3775:5747_culicoides_sonorensis_1449:1:12322	9.00E-16	59.32	1	1	59	59
Aaeg:AMP12	CSON002659-1_3775:5747_culicoides_sonorensis_1449:1:12322	5.00E-14	53.45	1	1	58	58
Aaeg:AMP6	CSON002659-1_3775:5747_culicoides_sonorensis_1449:1:12322	3.00E-13	51.67	1	1	60	59
Aaeg:AMP15	CSON002659-1_3775:5747_culicoides_sonorensis_1449:1:12322	6.00E-10	50	1	1	58	57
Aaeg:AMP5	CSON002659-1_3775:5747_culicoides_sonorensis_1449:1:12322	3.00E-13	49.15	1	1	59	59
Aaeg:AMP8	CSON002659-1_3775:5747_culicoides_sonorensis_1449:1:12322	7.00E-14	49.15	1	1	59	59
Aaeg:AMP10	CSON002659-1_3775:5747_culicoides_sonorensis_1449:1:12322	1.00E-09	38.71	1	1	62	62
Aaeg:AMP11	CSON002659-1_3775:5747_culicoides_sonorensis_1449:1:12322	5.00E-09	37.1	1	1	62	62
Aaeg:JAKSTAT2	CSON002696-1_21520:28776_culicoides_sonorensis_1450:1:36815	0	36.46	9	10	1132	1114
Aaeg:AMP16	CSON003561-1_30911:32061_culicoides_sonorensis_1664:1:44836	7.00E-21	53.52	35	9	105	79
Aaeg:TOLL10	CSON003737-1_32120:35979_culicoides_sonorensis_1710:1:41620	0	65.65	7	11	1283	1248
Aaeg:TOLL5	CSON003960-1_18040:25340_culicoides_sonorensis_175:1:228222	0	70.08	7	9	1307	1322
Aaeg:TOLL4	CSON003960-1_18040:25340_culicoides_sonorensis_175:1:228222	0	58.18	22	34	1289	1317
Aaeg:IMDPATH10	CSON004331-1_16949:18301_culicoides_sonorensis_1854:1:39857	7.00E-44	37.14	35	32	357	347

Aaeg:JAKSTAT1	CSON004944-1_14194:18968_culicoides_sonorensis_2012:1:45851	1.00E-176	34.79	82	23	1143	1148
Aaeg:TOLL11	CSON006589-1_14598:18241_culicoides_sonorensis_2472:1:22432	3.00E-62	30.24	91	209	672	886
Aaeg:TOLL3	CSON007335-1_9709:17284_culicoides_sonorensis_2797:1:17286	0	40.2	47	31	962	939
Aaeg:TOLL8	CSON007335-1_9709:17284_culicoides_sonorensis_2797:1:17286	5.00E-180	34.88	56	63	911	939
Aaeg:TOLL7	CSON007335-1_9709:17284_culicoides_sonorensis_2797:1:17286	1.00E-150	32.91	258	10	1165	939
Aaeg:TOLL1	CSON008073-1_142312:146241_culicoides_sonorensis_304:1:168790	0	73.3	1	1	1277	1286
Aaeg:IMDPATH2	CSON008729-1_60042:77119_culicoides_sonorensis_332:1:164081	4.00E-127	63.54	5	11	281	286
Aaeg:IMDPATH2	CSON008729-1_60042:77119_culicoides_sonorensis_332:1:164081	1.00E-11	29.77	362	458	492	581
Aaeg:TOLLPATH5	CSON008794-1_1:2081_culicoides_sonorensis_3374:1:12964	1.00E-12	27.59	291	207	455	380
Aaeg:AMP14	CSON009409-1_3728:4668_culicoides_sonorensis_3732:1:7574	7.00E-12	31.9	85	53	237	211
Aaeg:AMP14	CSON009409-1_3728:4668_culicoides_sonorensis_3732:1:7574	3.00E-11	29.08	127	35	263	172
Aaeg:TOLL6	CSON011712-1_1:1755_culicoides_sonorensis_5210:1:2896	2.00E-74	29.15	2	2	538	585
Aaeg:TOLLPATH2	CSON012655-1_65248:67773_culicoides_sonorensis_608:1:113328	7.00E-60	32.05	75	83	484	465
Aaeg:AMP1	CSON013028-1_35501:36647_culicoides_sonorensis_641:1:95367	3.00E-14	41.3	1	1	91	87
Aaeg:AMP2	CSON013028-1_35501:36647_culicoides_sonorensis_641:1:95367	3.00E-13	39.78	1	1	92	87
Aaeg:AMP3	CSON013028-1_35501:36647_culicoides_sonorensis_641:1:95367	2.00E-14	39.13	1	1	91	87
Aaeg:AMP4	CSON013028-1_35501:36647_culicoides_sonorensis_641:1:95367	3.00E-10	35	7	4	105	93
Aaeg:JAKSTAT3	CSON013044-1_72376:95260_culicoides_sonorensis_642:1:111517	0	63.49	1	1	736	740
Aaeg:JAKSTAT3	CSON013044-1_72376:95260_culicoides_sonorensis_642:1:111517	1.00E-56	26.69	29	739	732	1377
Aaeg:TOLLPATH1	CSON013585-1_15343:17080_culicoides_sonorensis_689:1:100925	3.00E-40	35.43	374	227	591	445

b)

Query	Target	evalue	% identity	qstart	sstart	qend	send
Cpip:TOLL6	CSON000010-1_12385:20136_culicoides_sonorensis_1004:1:76358	0	65.1	40	35	1357	1378
Cpip:IMDPATH3	CSON000146-1_188504:193471_culicoides_sonorensis_101:1:286939	6.00E-67	31.82	4	14	494	470
Cpip:IMDPATH4	CSON001226-1_213213:218118_culicoides_sonorensis_118:1:280338	0	47.23	7	4	797	743

Cpip:TOLLPATH4	CSON001312-1_215650:225787_culicoides_sonorensis_11:1:572898	2.00E-45	30.86	13	7	408	380
Cpip:TOLL3	CSON002070-1_13325:17028_culicoides_sonorensis_1342:1:30232	3.00E-96	32.43	15	210	595	886
Cpip:TOLLPATH5	CSON002111-1_133327:143976_culicoides_sonorensis_134:1:259523	6.00E-66	40.98	24	17	365	359
Cpip:AMP3	CSON002659-1_3775:5747_culicoides_sonorensis_1449:1:12322	4.00E-18	62.3	1	1	61	58
Cpip:AMP4	CSON002659-1_3775:5747_culicoides_sonorensis_1449:1:12322	1.00E-13	53.45	1	1	58	58
Cpip:AMP2	CSON002659-1_3775:5747_culicoides_sonorensis_1449:1:12322	2.00E-11	47.46	1	1	59	59
Cpip:AMP5	CSON002659-1_3775:5747_culicoides_sonorensis_1449:1:12322	1.00E-10	46.55	1	1	58	58
Cpip:JAKSTAT2	CSON002696-1_21520:28776_culicoides_sonorensis_1450:1:36815	0	34.33	13	4	1148	1112
Cpip:AMP1	CSON003561-1_30911:32061_culicoides_sonorensis_1664:1:44836	2.00E-21	52.78	29	8	100	79
Cpip:TOLL7	CSON003737-1_32120:35979_culicoides_sonorensis_1710:1:41620	0	66.93	72	4	1353	1248
Cpip:TOLL10	CSON003960-1_18040:25340_culicoides_sonorensis_175:1:228222	1.00E-164	69.73	83	10	449	375
Cpip:TOLL10	CSON003960-1_18040:25340_culicoides_sonorensis_175:1:228222	4.00E-101	62.21	425	1061	672	1322
Cpip:TOLL10	CSON003960-1_18040:25340_culicoides_sonorensis_175:1:228222	2.00E-62	67.95	680	1167	828	1322
Cpip:TOLL11	CSON003960-1_18040:25340_culicoides_sonorensis_175:1:228222	0	56.79	26	3	1302	1290
Cpip:TOLL10	CSON003960-1_18040:25340_culicoides_sonorensis_175:1:228222	2.00E-10	30.27	235	344	419	513
Cpip:TOLL10	CSON003960-1_18040:25340_culicoides_sonorensis_175:1:228222	5.00E-11	28.15	183	346	409	576
Cpip:TOLL10	CSON003960-1_18040:25340_culicoides_sonorensis_175:1:228222	7.00E-08	28.03	288	789	411	920
Cpip:IMDPATH6	CSON004331-1_16949:18301_culicoides_sonorensis_1854:1:39857	3.00E-43	38.37	38	35	354	347
Cpip:JAKSTAT1	CSON004944-1_14194:18968_culicoides_sonorensis_2012:1:45851	2.00E-175	35.34	72	21	1139	1147
Cpip:TOLL4	CSON006589-1_14598:18241_culicoides_sonorensis_2472:1:22432	1.00E-104	34.23	1	169	660	771
Cpip:TOLL2	CSON007335-1_9709:17284_culicoides_sonorensis_2797:1:17286	0	37.7	43	32	948	939
Cpip:TOLL8	CSON008073-1_142312:146241_culicoides_sonorensis_304:1:168790	0	73.93	1	1	1267	1288
Cpip:IMDPATH2	CSON008729-1_60042:77119_culicoides_sonorensis_332:1:164081	5.00E-125	56.36	17	15	344	336
Cpip:TOLLPATH7	CSON008794-1_1:2081_culicoides_sonorensis_3374:1:12964	1.00E-09	26.63	58	213	214	380
Cpip:IMDPATH7	CSON010320-1_1561:2350_culicoides_sonorensis_4170:1:6723	1.00E-22	44	28	36	126	135
Cpip:TOLL1	CSON011712-1_1:1755_culicoides_sonorensis_5210:1:2896	1.00E-46	28.42	33	224	384	585
Cpip:JAKSTAT4	CSON012361-1_1:1501_culicoides_sonorensis_5791:1:1501	1.00E-97	41.8	192	4	603	432

Cpip:TOLLPATH2	CSON012655-1_65248:67773_culicoides_sonorensis_608:1:113328	2.00E-79	34.84	36	53	485	465
Cpip:AMP6	CSON013028-1_35501:36647_culicoides_sonorensis_641:1:95367	9.00E-14	73.68	55	50	92	87
Cpip:JAKSTAT3	CSON013044-1_72376:95260_culicoides_sonorensis_642:1:111517	0	62.72	1	1	729	728
Cpip:JAKSTAT3	CSON013044-1_72376:95260_culicoides_sonorensis_642:1:111517	8.00E-55	28.93	177	852	678	1336
Cpip:JAKSTAT5	CSON013044-1_72376:95260_culicoides_sonorensis_642:1:111517	4.00E-116	36.6	14	22	609	702
Cpip:JAKSTAT5	CSON013044-1_72376:95260_culicoides_sonorensis_642:1:111517	8.00E-51	28.24	113	844	599	1340
Cpip:TOLLPATH1	CSON013585-1_15343:17080_culicoides_sonorensis_689:1:100925	7.00E-38	36.61	381	227	597	445

Table S11. Read mapping and estimate of assembly size, by read mapping using BWA, according to the method of Schell et al. [36].

**Number of libraries**	4
**Number of trimmed reads**	541,819,928
**Number of mapped nucleotides**	38,092,763,217
**% of reads mapped**	98
**% mapped nucleotides**	68.21
**Peak frequency base coverage**	125
**Estimated genome size (Mb)**	304.74

Table S12. BUSCO analysis using the Insecta data set (version 3.0.2) against the assembled genome sequence of six species, including *Culicoides sonorensis*.

Species	**Complete BUSCOs**	**Single copy, complete**	**Duplicate, complete**	**Fragmented**	**Missing**
Ae. aegytpi	97.0%	89.3%	7.3%	1.1%	1.9%
An. gambiae	99.4%	97.6%	1.8%	0.1%	0.5%
B. antarctica	97.4%	96.6%	0.8%	1.3%	1.3%
C. quinquefasciatus	96.8%	91.9%	4.9%	0.2%	2.4%
C. sonorensis	**97.1%**	**66.3%**	**30.8%**	**0.9%**	**2.0%**
D. melanogaster	99.7%	99.0%	0.7%	0.2%	0.1%

Table S13. Number of distinct InterPro and GO terms annotated to annotated proteins of *C. sonorensis* and six other Diptera species.

	C. sonorensis	***Ae. aegypti***	***An. gambiae***	***B. antarctica***	***C. quinquefasciatus***	***D. melanogaster***	***L. cuprinea***
InterPro terms	6461	6977	6984	6461	6861	6609	6771
GO terms	1952	2591	4214	1968	8230	8230	2110
GO slim terms	137	140	142	137	136	144	138

Table S14. Tandemly repeated gene arrays in *C. sonorensis* versus other insect species. Longest gene arrays in *C. sonorensis* with matching InterPro domain architecture and maximum 100,000 nucleotide between successive members. The longest array with the same domain signature from 3 other insect species is given for comparison.

**InterPro domains present**	***Culicoides sonorensis* gene array (gene IDs)**	**Number of genes in array in longest array containing this domain signature**			
		C. sonorensis	***Ae. aegypti***	***An. gambiae***	***D. melanogaster***
IPR002557	10 genes: CSON015602, CSON015608, CSON015610-CSON015617	10	-	-	2
	9 genes: CSON009194-CSON009200, CSON009202				
IPR011021, IPR011022, IPR014756	9 genes: CSON013877, CSON013878, CSON01388, CSON013881, CSON013886, CSON013888, CSON013890, CSON013891, CSON013893	9	10	5	10
	8 genes: CSON004652-CSON004656, CSON004661, CSON004662, CSON004664				
IPR007588	8 genes: CSON002384-CSON002391	8	12	-	20
