# Supplementary material for: The genome of the biting midge Culicoides sonorensis and gene expression analyses of vector competence for bluetongue virus
Source: BMC Genomics. 2018 Aug 22;19:624. doi: 10.1186/s12864-018-5014-1 (PMC6106943; doi:10.1186/s12864-018-5014-1)
Supplement: Supplementary file 4 — Figure S1. Distribution of Cq values from RT-qPCR used to detect BTV-1 virus infection in the two virus-feeding experiments (in blue and orange) of C. sonorensis. The vertical, dashed line corresponds to a Cq value of 27 used to differentiate vector competent from refractory females. Figure S2. Classification and functional distribution of the genes differentially expressed between vector competent and refractory C. sonorensis per the Gene Ontology level 6. Blue: Molecular Function; Green: Cellular Component; Pink: Biological Process. Figure S3. Number of genes in the Toll (a), Imd (b) and Jak/Stat (c) pathways that have been duplicated or lost in different species of Diptera as identified using the Ensembl Compara pipeline. (DOCX 158 kb) [file 12864_2018_5014_MOESM4_ESM.docx]

**Figure S1.** Distribution of C_q_ values from RT-qPCR used to detect BTV-1 virus infection in the two virus-feeding experiments (in blue and orange) of *C. sonorensis*. The vertical, dashed line corresponds to a C_q_ value of 27 used to separate vector competent from refractory females.


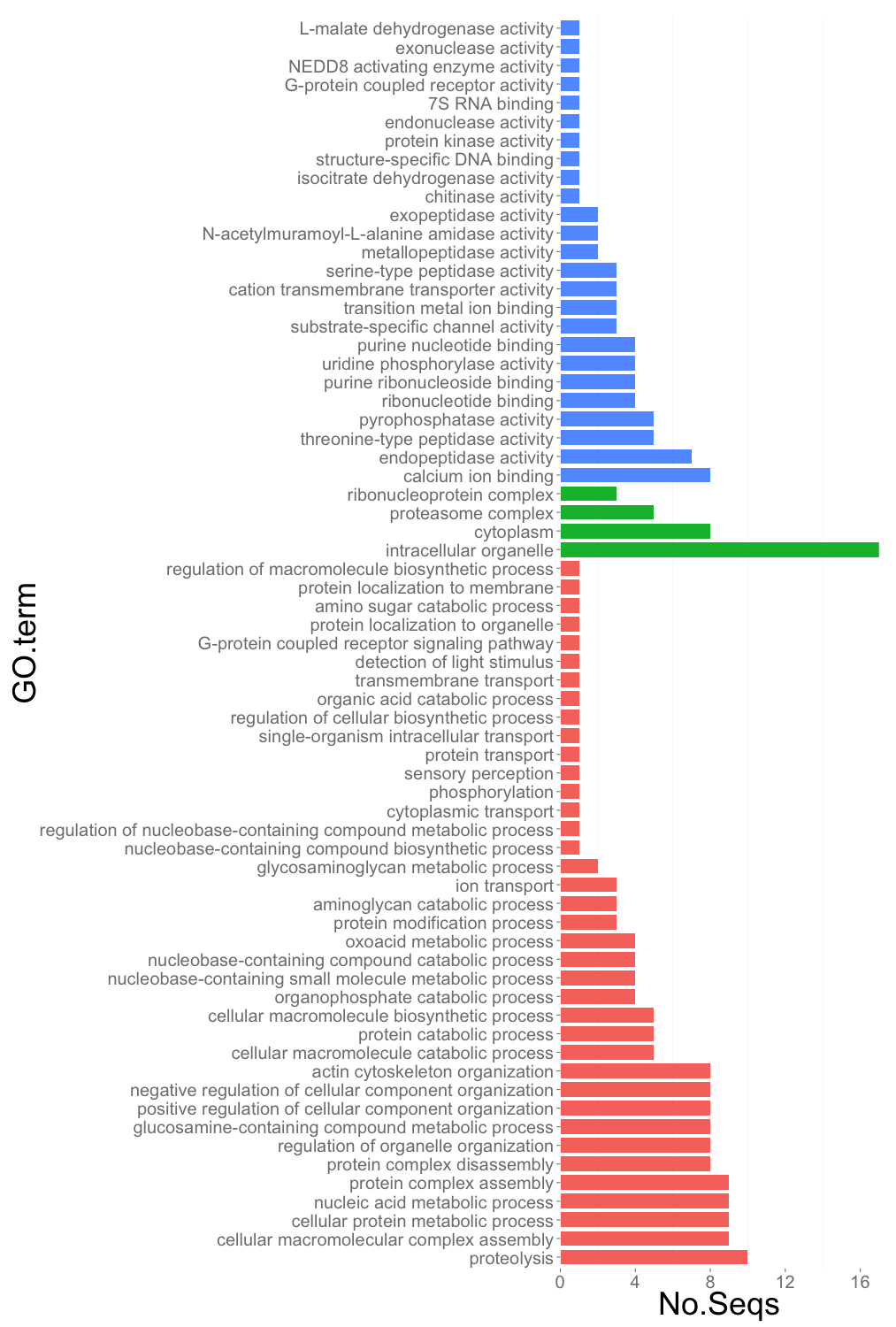
 **Figure S2.** Classification and functional distribution of the genes differentially expressed between vector competent and refractory *C. sonorensis* per the Gene Ontology level 6. Blue: Molecular Function; Green: Cellular Component; Pink: Biological Process.

a)

b)

c)

**Figure S3.** Number of genes in the Toll (a), Imd (b) and Jak/Stat (c) pathways that have been duplicated or lost in different species of Diptera as identified using the Ensembl Compara pipeline.
